# Fractalkine signaling regulates oligodendroglial cell genesis from SVZ precursor cells

**DOI:** 10.1016/j.stemcr.2021.06.010

**Published:** 2021-07-15

**Authors:** Adrianne E.S. Watson, Monique M.A. de Almeida, Nicole L. Dittmann, Yutong Li, Pouria Torabi, Tim Footz, Gisella Vetere, Danny Galleguillos, Simonetta Sipione, Astrid E. Cardona, Anastassia Voronova

**Affiliations:** 1 Department of Medical Genetics, Faculty of Medicine and Dentistry, University of Alberta, 8-39 Medical Sciences Building, Edmonton, AB T6G 2H7, Canada; 2 Women and Children's Health Research Institute, 5-083 Edmonton Clinic Health Academy, University of Alberta, 11405 87 Avenue NW Edmonton, AB T6G 1C9, Canada; 3 Neuroscience and Mental Health Institute, Faculty of Medicine and Dentistry, University of Alberta, Edmonton, AB T6G 2E1, Canada; 4 Team Cerebral Codes and Circuits Connectivity (C4), Plasticité du cerveau, ESPCI Paris, CNRS, PSL University, 75005 Paris, France; 5 Neurosciences and Mental Health Program, Hospital for Sick Children, Toronto, ON M5G 1L7, Canada; 6 Department of Pharmacology, Faculty of Medicine and Dentistry, University of Alberta, Edmonton, AB T6G 2H7, Canada; 7 Department of Biology, University of Texas at San Antonio, San Antonio, TX 78249, USA; 8 Multiple Sclerosis Centre and Department of Cell Biology, Faculty of Medicine and Dentistry, University of Alberta, Edmonton, AB T6G 2H7, Canada

**Keywords:** neural stem cell, SVZ, oligodendrocyte, myelin, Regeneration, OPC, oligodendrocyte precursor, chemokine, fractalkine, CX3CR1

## Abstract

Neural and oligodendrocyte precursor cells (NPCs and OPCs) in the subventricular zone (SVZ) of the brain contribute to oligodendrogenesis throughout life, in part due to direct regulation by chemokines. The role of the chemokine fractalkine is well established in microglia; however, the effect of fractalkine on SVZ precursor cells is unknown. We show that murine SVZ NPCs and OPCs express the fractalkine receptor (CX3CR1) and bind fractalkine. Exogenous fractalkine directly enhances OPC and oligodendrocyte genesis from SVZ NPCs *in vitro*. Infusion of fractalkine into the lateral ventricle of adult NPC lineage-tracing mice leads to increased newborn OPC and oligodendrocyte formation *in vivo*. We also show that OPCs secrete fractalkine and that inhibition of endogenous fractalkine signaling reduces oligodendrocyte formation *in vitro*. Finally, we show that fractalkine signaling regulates oligodendrogenesis in cerebellar slices *ex vivo*. In summary, we demonstrate a novel role for fractalkine signaling in regulating oligodendrocyte genesis from postnatal CNS precursor cells.

## Highlights

• Postnatal and adult murine SVZ precursor cells express *Cx3cr1* and bind fractalkine
• Fractalkine enhances OPC and oligodendrocyte formation from SVZ NPCs
• Inhibition of fractalkine signaling reduces SVZ NPC and OPC oligodendrogenesis
• Fractalkine signaling regulates oligodendrogenesis in *ex vivo* cerebellar slices

## Graphical abstract

**Figure:**
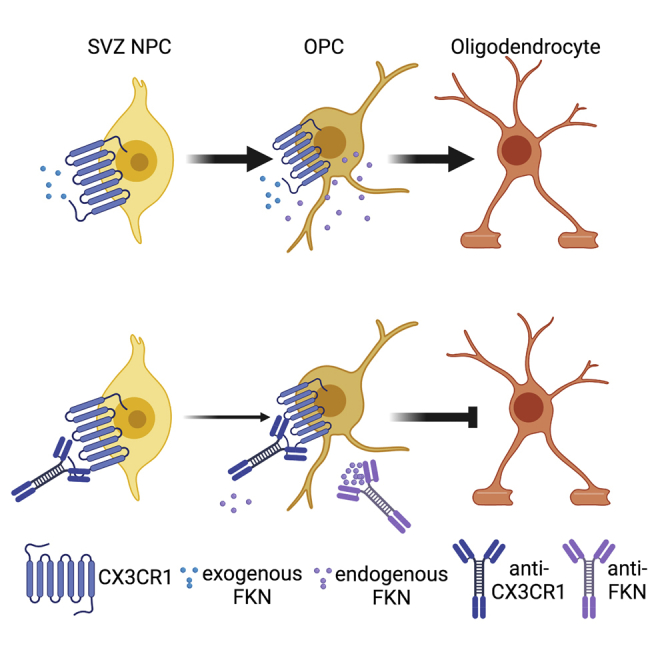

## Teaser

Watson et al. demonstrate that murine SVZ NPCs and OPCs express *Cx3cr1* and bind fractalkine. Exogenous fractalkine increases oligodendrogenesis from SVZ NPCs and OPCs. They also show that OPCs secrete fractalkine and that inhibition of endogenous fractalkine signaling reduces SVZ oligodendrocyte formation. Finally, they show that fractalkine signaling is an important regulator of oligodendrocyte genesis in other brain areas, such as the cerebellum.

## Introduction

Myelin, which is formed by oligodendrocytes in the central nervous system (CNS), plays an essential role in brain homeostasis by protecting nerve axons and propagating neuronal signal transduction. However, the role of oligodendrocytes and myelin extends beyond axonal support. A decrease in adult oligodendrocyte and/or new myelin genesis impairs motor skill learning, axon function, and memory formation (McKenzie et al., 2014; Steadman et al., 2019; Xiao et al., 2016). Increase in *de novo* oligodendrocyte formation and myelination can rescue motor dysfunctions (Schneider et al., 2016), spatial memory decline (Wang et al., 2020), fear memory consolidation (Pan et al., 2020), and abnormal social behaviors (Barak et al., 2019).

Resident neural precursor cells (NPCs) in the adult brain can be recruited for increased oligodendrogenesis and myelination (Murphy and Franklin, 2017). Notably, subventricular zone (SVZ) NPCs have the potential to generate oligodendrocytes, as well as astrocytes and neurons, throughout life (Maki et al., 2013; Obernier and Alvarez-Buylla, 2019). In normal homeostatic conditions, a subset of SVZ NPCs generates oligodendrocyte precursor cells (OPCs) and oligodendrocytes that populate the corpus callosum, striatum, and fimbria fornix (Menn et al., 2006). The recruitment of SVZ NPCs to form oligodendrocytes is drastically increased upon a demyelinating injury (Menn et al., 2006; Xing et al., 2014).

Intriguingly, NPCs and OPCs express chemokine receptors in the developing and adult CNS (reviewed in Watson et al., 2020). While the original defined role of chemokines is chemotaxis, they also directly participate in NPC and OPC proliferation, survival, migration, and differentiation. For example, CXCL1 and CXCL12 increase NPC/OPC proliferation and oligodendrocyte differentiation, whereas CXCL10 decreases OPC differentiation (Watson et al., 2020).

We have previously shown that developmental cortical oligodendrogenesis can be instructed by the chemokine fractalkine (CX3CL1, herein referred to as FKN) (Voronova et al., 2017). FKN secreted by cortical inhibitory neurons acts on CX3CR1 (FKN receptor) expressed in murine embryonic cortical progenitors and instructs them to differentiate into oligodendrocytes (Voronova et al., 2017). Whether FKN-CX3CR1 signaling can enhance recruitment of SVZ precursors for oligodendrocyte formation in the adult CNS remains to be addressed.

We demonstrate that postnatal and adult SVZ NPCs and OPCs express *Cx3cr1* and bind FKN. Exogenous FKN increases oligodendrogenesis from SVZ precursor cells *in vitro* and *in vivo*. We also show that OPCs secrete FKN and that inhibition of endogenous FKN signaling impairs oligodendrocyte genesis. Together, our data demonstrate FKN signaling as an important regulator of oligodendrocyte formation from precursor cells in the SVZ niche. Finally, we also show that the role of FKN signaling in oligodendrocyte genesis is preserved in the cerebellum.

## Results

### SVZ NPCs and OPCs express *Cx3cr1* and bind FKN

**Figure 1:**
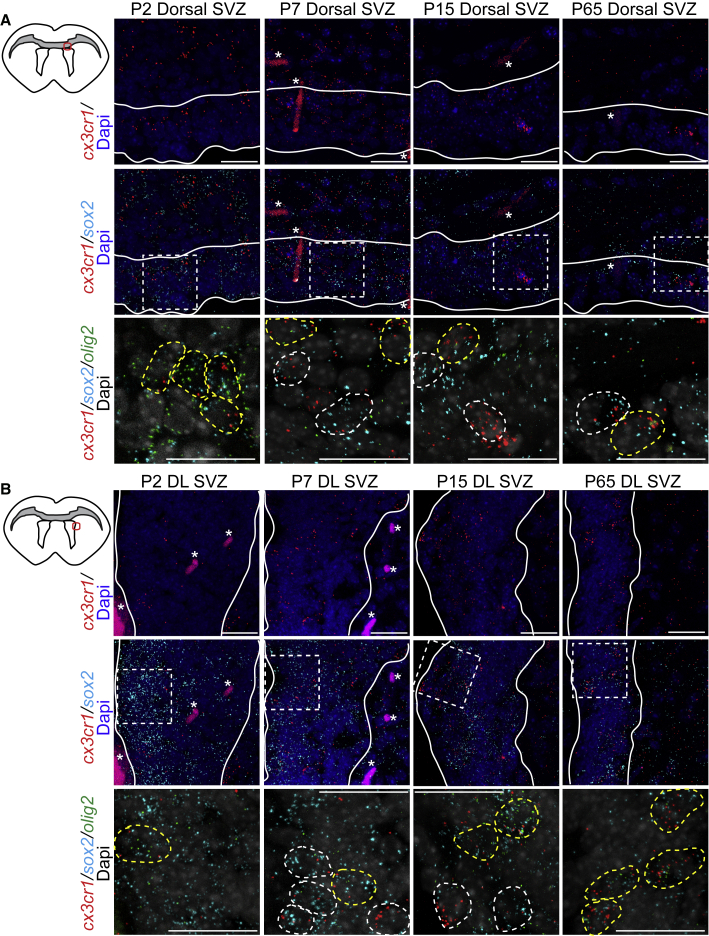
SVZ NPCs and OPCs express *Cx3cr1 in vivo* (A and B) Top left: approximate location of dorsal (A) and dorsolateral (DL) (B) SVZ (red circle) images shown here. Top row: RNAscope analysis of dorsal (A) and DL (B) SVZ from P2, P7, P15, and P65 SVZ for *Cx3cr1* (red) mRNA. Middle row: double-label RNAscope analysis for *Cx3cr1* (red) and *Sox2* (cyan) mRNAs. Hatched boxes indicate section shown at higher magnification in bottom row. Bottom row: triple-label RNAscope analysis for *Cx3cr1* (red), *Sox2* (cyan), and *Olig2* (green). White dashed circles indicate *Cx3cr1*^+^*Sox2*^+^ and yellow dashed circles *Cx3cr1*^+^*Sox2*^+^*Olig2*^+^ cells. Asterisks indicate blood vessels. n = 2–3 for each age. Scale bars, 20 μm. See also Figure S1.

In addition to microglia, which express high level of CX3CR1 (Limatola and Ransohoff, 2014), neurons and NPCs also express CX3CR1, albeit at a lower level (Ji et al., 2004; Meucci et al., 2000; Mizrak et al., 2019; Voronova et al., 2017; Wang et al., 2018). First, RNAscope analysis using probes against *Cx3cr1*, *Sox2*, and *Olig2* mRNA confirmed that *Cx3cr1* mRNA was expressed in *Sox2*^+^*Olig2*^−^ NPCs and *Sox2*^+^*Olig2*^+^ OPCs in the dorsal and dorsolateral SVZ of postnatal day (P) 2, P7, P15, and P65 mice (Figure 1) and the ventrolateral SVZ of P7, P15, P65, but not P2, mice (Figures S1A and S1B).

**Figure 2:**
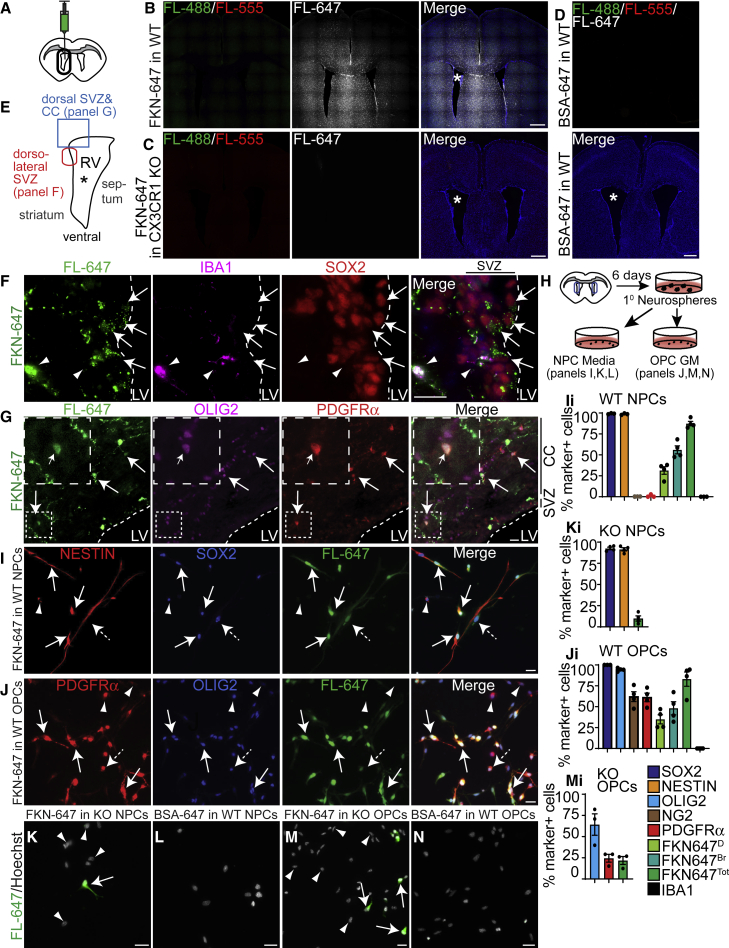
SVZ NPCs and OPCs bind FKN *in vivo* and *in vitro* (A) WT or CX3CR1^KO^ mice were injected once with FKN-647 and/or BSA-647 into the right lateral ventricle (RV, asterisk). (B–D) Coronal section of WT brain infused with FKN-647 (B) or BSA-647 (D), or CX3CR1^KO^ brain infused with FKN-647 (C). Fluorescence in all channels was captured using identical settings. (E) Approximate location of images in (F) and (G). (F) Dorsolateral SVZ of FKN-647 infused WT brain immunostained for IBA1 (purple) and SOX2 (red). Arrows indicate FKN-647^+^SOX2^+^ and arrowheads FKN-647^+^SOX2^−^IBA1^+^ cells. (G) Dorsal SVZ and corpus callosum (CC) of FKN-647 infused WT brain immunostained for OLIG2 (purple) and PDGFRα (red). Arrows indicate FKN-647^+^OLIG2^+^PDGFRα^+^ cells. Fluorescence (FL) in far-red channel (FL-647) in (F) and (G) is pseudo-colored in green. Dashed lines indicate SVZ boundary. LV, lateral ventricle. (H) Schematic of microglia-free SVZ NPC and OPC cultures. (I) 1-DIV WT NPCs incubated with FKN-647 (green) and immunostained for NESTIN (red) and SOX2 (blue). Arrows indicate NESTIN^+^SOX2^+^FKN-647^Bright (Br)+^, dashed arrow NESTIN^+^SOX2^+^FKN-647^Dim (D)+^, and arrowhead NESTIN^+^SOX2^+^FKN-647^−^ cells. (J) 2-DIV WT OPCs incubated with FKN-647 (green) and immunostained for PDGFRα (red) and OLIG2 (blue). Arrows indicate PDGFRα^+^OLIG2^+^FKN-647^Br+^, dashed arrow PDGFRα^+^OLIG2^+^FKN-647^D+^, and arrowhead PDGFRα^+^OLIG2^+^FKN-647^−^ cells. (K and M) 1-DIV NPCs (K) or 2-DIV OPCs (M) from CX3CR1^KO^ SVZ incubated with FKN-647 (green). Arrows indicate FKN-647^+^ cells, arrowheads FKN-647^−^ cells. (L and N) 1-DIV NPCs (L) or 2-DIV OPCs (N) from WT SVZ incubated with BSA-647 (green). Fluorescence in far-red channel (FL-647) was captured using identical settings in (I) to (N). (Ii–Mi) Quantification of (I), (K), (J), and (M). Error bars represent SEM, n = 3–4 experiments. Scale bars, 20 μm (A, F, G, I–N) and 500 μm (B–D). See also Figure S2.

To find out whether FKN can bind SVZ precursors *in vivo*, we injected FKN conjugated to fluorophore Alexa Fluor 647 (FKN-647) into the lateral ventricle of adult wild-type (WT) mice, or mice with a constitutive knockout of CX3CR1 (CX3CR1^KO^) (Cardona et al., 2018) (Figure 2A). As an additional control, WT mice were also injected with BSA-647. Analysis of fluorescent signal in the far-red channel demonstrated widespread diffusion of FKN-647 throughout the brain parenchyma in WT mice (Figure 2B), but not CX3CR1^KO^ mice (Figure 2C), indicating that FKN-647 bound specifically to cells that express CX3CR1. Furthermore, there was no detectable signal in BSA-647-infused WT brain (Figure 2D). Immunostaining confirmed that a fluorescent signal in the far-red channel of FKN-647-infused brains was detected in SOX2^+^IBA1^−^ SVZ NPCs (Figures 2E and 2F) and was localized on the surface of SVZ NPCs (Figures S2A–S2D). Quantification revealed that ∼40% of SOX2^+^GFAP^+^ and ∼40% of total SOX2^+^ NPCs were positive for FKN-647, with no difference between dorsal, dorsolateral, and ventrolateral regions of the SVZ (data not shown). This is in line with a previous report which showed that *Cx3cr1* is enriched in activated NPCs but not quiescent NPCs (SVZ astrocytes), ependymal cells, or neuroblasts (Mizrak et al., 2019) (Figure S2E). Fluorescent signal was also evident in PDGFRα^+^OLIG2^+^ OPCs in the dorsal SVZ and adjacent corpus callosum (Figures 2E and 2G). Similar sections in BSA-647-infused WT and FKN-647-infused CX3CR1^KO^ mice imaged under identical settings did not yield a positive signal (data not shown). FKN-647 also associated with IBA1^+^ microglia (Figures 2F and S2F), validating this approach, as microglia are known to express high levels of CX3CR1.

To elucidate the role of FKN signaling specifically in SVZ precursor cells, we generated microglia-free NPC and OPC cultures (Figure 2H). Cells isolated from either WT or CX3CR1^KO^ P7 SVZ were cultured as primary neurospheres, which were then dissociated and incubated as adherent cells either for 1 day *in vitro* (DIV) in NPC medium containing fibroblast growth factor (FGF) and epidermal growth factor (EGF), or for 2 DIV in OPC growth medium (GM) containing FGF and platelet-derived growth factor AA (PDGF-AA) (Figure 2H). WT NPC cultures were nearly 100% positive for NPC markers SOX2 and NESTIN (Figures 2I and 2Ii), but not OPC markers NG2 or PDGFRα (Figure 2Ii). WT OPC cultures were composed predominantly of OPC cells, as they were 100% SOX2^+^, 94.32% ± 0.94% OLIG2^+^, 62.03% ± 5.91% NG2^+^, and 61.12% ± 5.14% PDGFRα^+^ (Figures 2J and 2Ji). Importantly, none of the cells were positive for the microglial marker IBA1 (Figures S2G–S2I). CX3CR1^KO^ NPC cultures displayed high levels of SOX2^+^ and NESTIN^+^ cells (92.5% ± 1.5% and 91.5% ± 1.9%, respectively), whereas CX3CR1^KO^ OPC cultures had reduced proportions of OLIG2^+^ and PDGFRα^+^ cells (64.2% ± 12.7% and 13.9% ± 4.6%, respectively) (Figures 2Ki and 2Mi). This supports and expands our previous work, in which CX3CR1^KO^ mice showed reduced OPCs in the neonatal cortex (Voronova et al., 2017).

In these cultures, both WT NPCs and OPCs exhibited robust binding of FKN-647 (Figures 2I and 2J) but not BSA-647 (Figures 2L and 2N). 87.40% ± 2.66% NPCs bound FKN-647, which could be further divided into two groups: FKN-647^BRIGHT^ (56.03% ± 4.80%) and FKN-647^DIM^ (31.37% ± 4.52%) cells (Figures 2I and 2Ii). OPC cultures showed 48.13% ± 7.84% FKN-647^BRIGHT^ and 34.89% ± 5.42% FKN-647^DIM^ OPCs yielding overall 83.02% ± 8.53% FKN-647^+^ OPCs (Figures 2J and 2Ji). The distinct levels of FKN-647 fluorescence may represent varying levels of FKN receptor expression in these precursor cells, reflecting the heterogeneity of these cell groups (Marques et al., 2018; Mizrak et al., 2019). Importantly, CX3CR1^KO^ NPCs and OPCs displayed greatly reduced FKN-647 binding (9.9% ± 3.0% and 21.8% ± 4.7%, respectively) (Figures 2K, 2Ki, 2M, and 2Mi). Residual FKN-647 binding in cultured cells may reflect expression of other receptors that can bind FKN, such as integrins (Fujita et al., 2012), non-specific binding, or binding via a yet unidentified receptor.

Together, these results demonstrate that postnatal SVZ NPCs and OPCs express *Cx3cr1* and bind FKN. These data support and extend previous reports showing that postnatal NPCs and OPCs express *Cx3cr1* as determined via RNA sequencing (Marques et al., 2018; Mizrak et al., 2019; reviewed in Watson et al., 2020).

### FKN increases oligodendrocyte genesis from SVZ NPCs in culture

**Figure 3:**
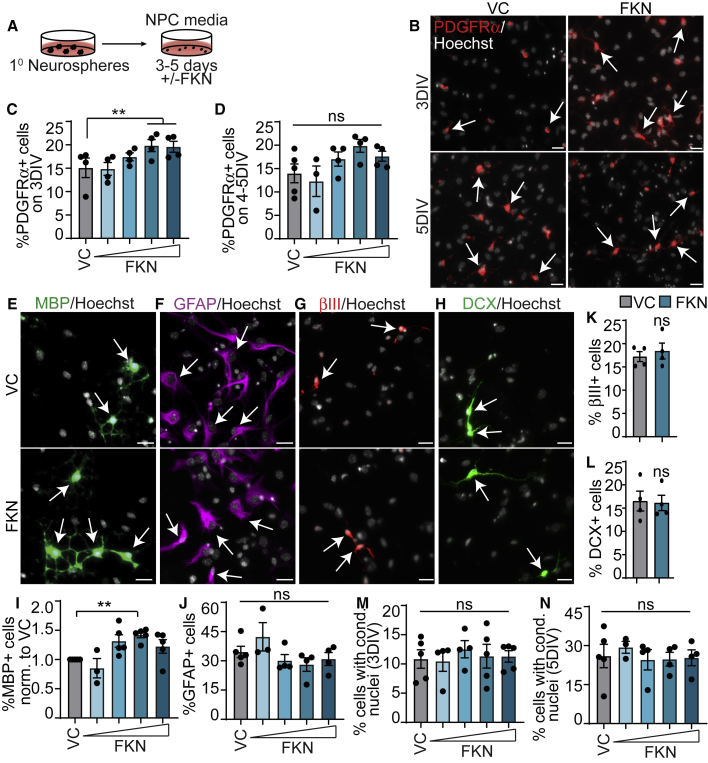
FKN increases oligodendrogenesis from SVZ NPCs *in vitro* (A) Schematic: P7 SVZ primary neurosphere cells were cultured as adherent NPCs for 3–5 DIV with 25–500 ng/mL FKN or VC. Representative images show cells incubated with 250 ng/mL FKN. (B) 3-DIV or 5-DIV NPCs cultured with VC (left) or FKN (right) and immunostained for PDGFRα (red). (C and D) Quantification of (B). (E–H) 4- to 5-DIV NPCs cultured with VC (top) or FKN (bottom) and immunostained for MBP (green, E), GFAP (purple, F), βIII (red, G), and DCX (green, H). (I–N) Quantification of (E) to (H). Results were normalized to VC in (I). In all images arrows indicate marker-positive cells. Cells were counterstained with Hoechst (gray). Graphs were analyzed with one-way ANOVA followed by Dunnett's multiple comparisons test, except (K) and (L), analyzed with paired t test. Error bars represent SEM, n = 3–5 experiments. ns, not significant. Scale bars, 20 μm.

To test whether FKN can mediate oligodendrocyte genesis from NPCs, we cultured WT SVZ NPC monolayers for 3–5 days with varying concentrations of FKN (Figure 3A). Immunostaining at 3 DIV revealed an FKN-mediated significant ∼38% increase in PDGFRα^+^ OPCs (Figures 3B and 3C) in a concentration-dependent manner when compared with vehicle control (VC, PBS). By 4–5 days, there was a trending increase in PDGFRα^+^ cells in the presence of FKN, though not significant (Figures 3B and 3D). However, there was a statistically significant ∼40% increase in MBP^+^ oligodendrocytes (Figures 3E and 3I) on 5DIV in a concentration-dependent manner in the presence of FKN. The greatest effect was seen with the intermediate FKN concentration (250 ng/mL).

As NPCs can produce astrocytes and neurons in addition to oligodendrocytes, we examined whether FKN may bias precursor cells to produce oligodendroglia at the expense of these cell types. Immunostaining revealed no change in GFAP^+^ astrocytes (Figures 3F and 3J), DCX^+^ neuroblasts (Figures 3H and 3L), or βIII^+^ neurons (Figures 3G and 3K) in cultures incubated with the most effective concentration of FKN (250 ng/mL). Since FKN has been shown to reduce neuronal cell death in stroke and synucleinopathy mouse models (Cipriani et al., 2011; Nash et al., 2015), we then considered that FKN may have an effect on cell death. However, FKN did not affect the number of cells with condensed nuclei at either time point (Figures 3M and 3N). Together, this indicates that FKN enhances the production of OPCs and oligodendrocytes from SVZ NPCs *in vitro*.

### FKN does not affect NPC or OPC proliferation

**Figure 4:**
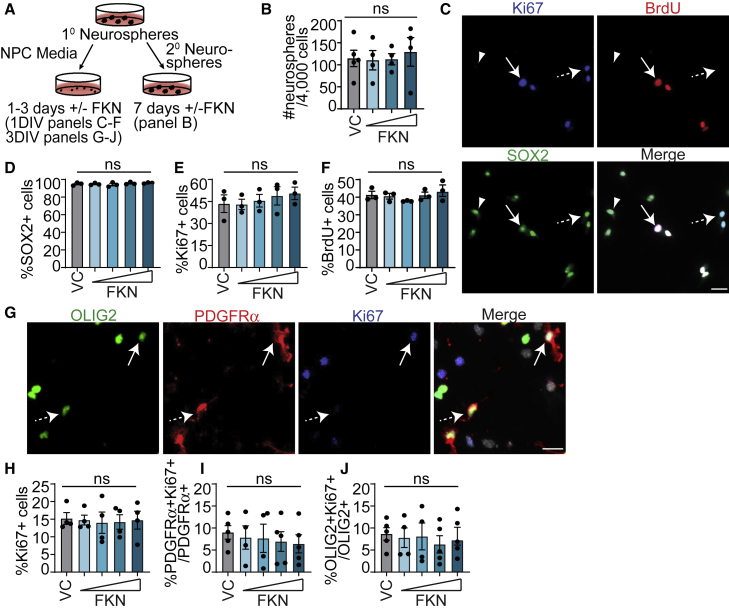
FKN does not affect NPC or OPC proliferation (A) Schematic: P7 SVZ primary neurosphere cells were cultured as adherent NPCs for 1 DIV (C–F) or 3 DIV (G–J), or as secondary neurospheres for 7 DIV (B) with 25–500 ng/mL FKN or VC. (B) Number of secondary neurospheres generated from 4,000 primary neurosphere cells. (C) 1-DIV NPCs in VC and immunostained for Ki67 (blue), BrdU (red), and SOX2 (green). Arrow indicates a Ki67^+^SOX2^+^BrdU^+^ cell, dashed arrow Ki67^+^SOX2^+^BrdU^−^ cell, and arrowhead SOX2^+^Ki67^−^BrdU^−^ cell. (D–F) Quantification of (C). (G) 3-DIV NPCs in VC and immunostained for OLIG2 (green), PDGFRα (red), and SOX2 (blue). Arrow indicates an OLIG2^+^PDGFRα^+^Ki67^+^ cell and dashed arrow OLIG2^+^PDGFRα^+^Ki67^−^ cell. (H–J) Quantification of (G). In all images cells were counterstained with Hoechst (gray). Error bars represent SEM, n = 4–5 experiments. All graphs were analyzed with one-way ANOVA followed by Dunnett's multiple comparisons test. ns, not significant. Scale bars, 20 μm.

We then asked whether FKN achieves the observed increase in the proportion of oligodendroglial cells by modulating NPC and/or OPC proliferation. First, exogenous FKN did not affect the number of secondary neurospheres formed when compared with VC (PBS, Figures 4A and 4B). Adherent 1-DIV NPCs similarly did not show differences in the proportion of Ki67^+^ (Figure 4E), bromodeoxyuridine (BrdU^+^) (Figure 4F), or SOX2^+^ cells (Figure 4D) in FKN versus VC.

**Figure 5:**
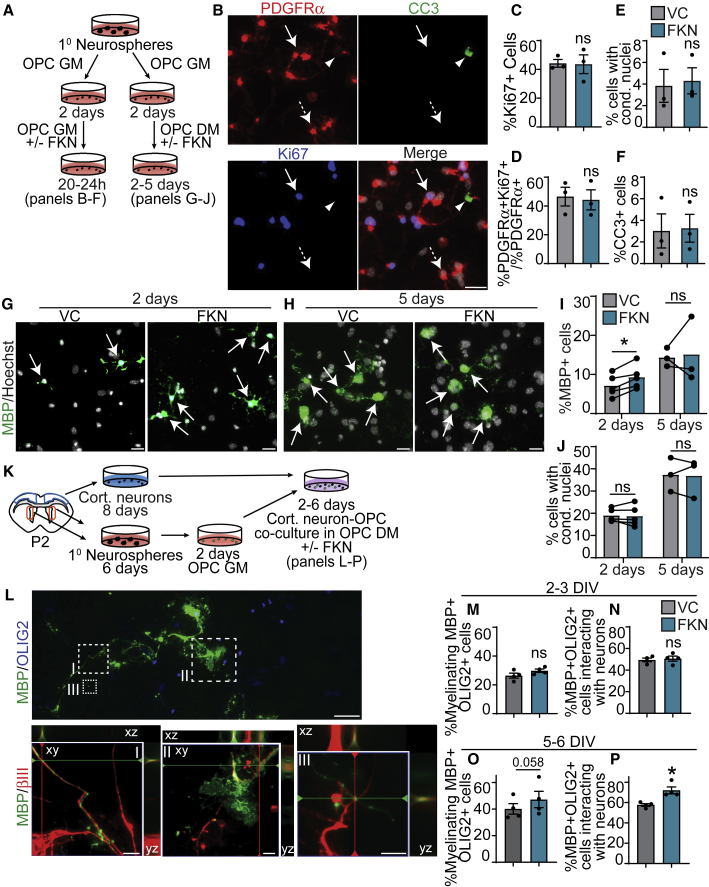
FKN accelerates SVZ OPC differentiation (A) Schematic: P7 SVZ primary neurosphere cells were cultured in OPC GM for 2 DIV, followed by either treatment with 250 ng/mL FKN or VC in OPC GM for 20–24 h (left, B–F) or in OPC differentiation medium (DM) for 2–5 days (right, G–J). (B) Representative images from OPCs in GM supplemented with VC, immunostained for PDGFRα (red), cleaved caspase-3 (CC3, green), and Ki67 (blue). Arrow indicates PDGFRα^+^Ki67^+^CC3^−^ cell, dashed arrow PDGFRα^+^Ki67^−^CC3^−^ cell, and arrowhead PDGFRα^+^Ki67^−^CC3^+^ cell. (C–F) Quantification of (B). (G and H) OPCs cultured in DM with VC (left) or FKN (right) for 2 DIV (G) or 5 DIV (H), immunostained for MBP (green) and counterstained with Hoechst (gray). Arrows indicate MBP^+^ cells. (I and J) Quantification of (G) and (H). (K) Schematic: cortical neurons and SVZ OPCs derived from P1-2 CD1 pups were co-cultured for 2–6 days in OPC DM with FKN/VC. (L) Cortical neuron-OPC co-culture, immunostained for MBP (green), OLIG2 (blue), and βIII (red). Top: overlay of MBP and OLIG2. Hatched boxes are shown at higher magnification in bottom row. Bottom: magnified image of hatched boxes represent an orthogonal slice through a z-stack. (M–P) Quantification of myelinating (M and O) or interacting (N and P) MBP^+^OLIG2^+^ expressed as percentage of total number of MBP^+^OLIG2^+^ cells after 2–3 DIV (M and N) and 5–6 DIV (O and P) of co-culture. Error bars represent SEM, n = 3–5 experiments. All graphs were analyzed with paired t test. ns, not significant. Scale bars, 20 μm (B and G), 50 μm (L) and 10 μm (L, insets).

To determine whether FKN could mediate OPC proliferation, we analyzed NPC monolayer cultures at 3 DIV, when we detected 10%–20% PDGFRα^+^ OPCs (Figure 3C). FKN had no effect on the proportion of Ki67^+^ cells (Figures 4G and 4H) or the proliferative index of PDGFRα^+^ or OLIG2^+^ cells when compared with VC (Figures 4I and 4J). To corroborate these results, we generated SVZ OPCs, which were then incubated with VC or 250 ng/mL FKN in OPC GM for 20–24 h (Figure 5A). There was no change in the proportion of Ki67^+^ cells or the proliferative index of PDGFRα^+^ cells (Figures 5B–5D). In addition, there was no effect on cells with condensed nuclei or CC3^+^ cells (Figures 5E and 5F).

This suggests that the pro-oligodendrogenic effect of FKN is due to enhanced NPC commitment and/or OPC differentiation, rather than modulation of proliferation or cell death.

### FKN accelerates oligodendrocyte differentiation from OPCs *in vitro*

To corroborate the effect of FKN on OPC differentiation using an independent platform, we subjected SVZ OPCs to medium supplemented with thyroid hormone T3 (differentiation medium [DM]) and FKN or VC. OPCs incubated with FKN showed a statistically significant 31.52% ± 11.77% increase in MBP^+^ cells at 2 DIV but not 5 DIV (Figures 5G–5I). The proportion of cells with condensed nuclei was unaffected at either time point (Figure 5J). Thus, exogenous FKN is sufficient to accelerate OPC differentiation into oligodendrocytes.

To assess whether FKN can increase the number of myelinating oligodendrocytes, we co-cultured SVZ OPCs with cortical neurons in DM with FKN or VC (Figure 5K). MBP^+^OLIG2^+^ oligodendrocytes were scored based on their interaction with βIII^+^ neurons (Figure 5L). Interacting oligodendrocytes were defined as a sum of “contacting” (Figure 5L, inset III) and “myelinating” cells (Figure 5L, insets I–II). By 2–3 DIV, there was no difference in either myelinating or interacting oligodendrocytes (Figures 5M and 5N). However, after 5–6 DIV, FKN-treated cultures showed a trending increase in myelinating oligodendrocytes that did not reach statistical significance and a statistically significant 16.65% ± 4.40% increase in the proportion of oligodendrocytes interacting with axons (Figures 5O and 5P). Therefore, FKN increases the proportion of oligodendrocytes that interact with cortical neurons, which may have implications for myelination.

### FKN enhances oligodendroglial lineage cell formation from SVZ NPCs *in vivo*

**Figure 6:**
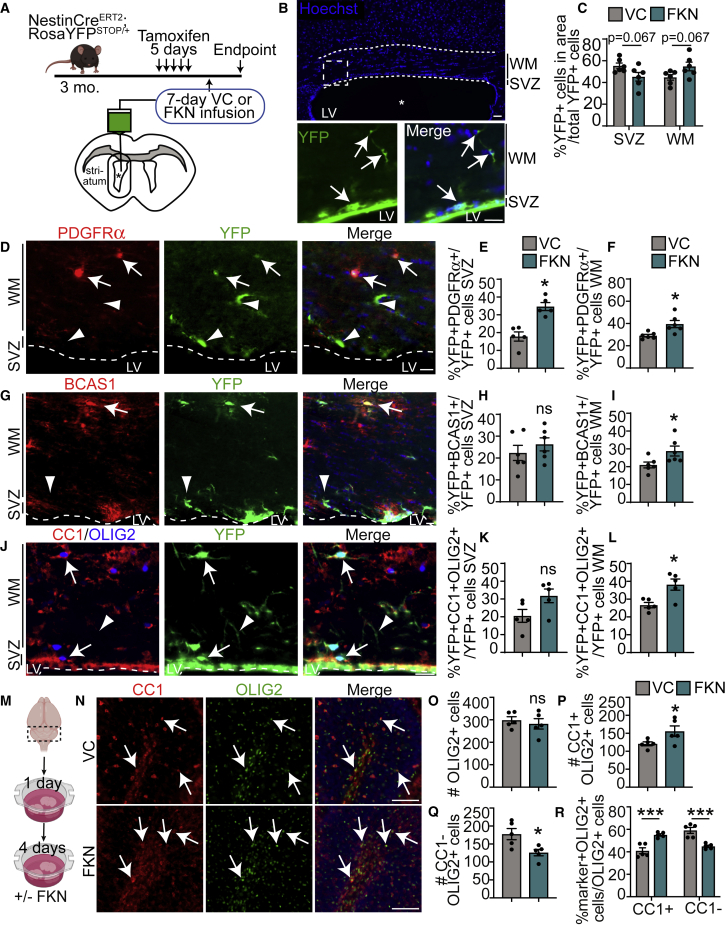
FKN enhances oligodendrogenesis from SVZ NPCs *in vivo* and from cerebellar OPCs *ex vivo* (A) Schematic: NestinCre^ERT2^;RosaYFP^STOP/+^ were injected with tamoxifen and infused with FKN or VC into the lateral ventricle (asterisk) for 7 days via osmotic mini-pump. Images from corpus callosum lining dorsally to the infused ventricles were analyzed in (B) to (L). (B) Representative image of VC-infused ventricle. Dashed borders indicate an area analyzed in (C) to (L) and encompass SVZ and white matter (WM). Bottom: hatched box from top shown at higher magnification. Arrows indicate YFP^+^ cells. (C) Analysis of YFP^+^ cell distribution in (B). (D, G, and J) Dorsal SVZ and WM in FKN-infused ventricle and immunostained for YFP (green) and PDGFRα (red, D), BCAS1 (red, G), or CC1 (red, J) and OLIG2 (blue, J). Arrows indicate marker-positive YFP^+^ cells, and arrowheads marker-negative YFP^+^ cells. Dashed line indicates SVZ boundary. LV, lateral ventricle. (E and F) Quantification of (D). (H and I). Quantification of (G). (K and L) Quantification of (J). n = 5–6 mice from three litters. (M) Schematic: cerebellar slices from P10–P11 CD1 mice were cultured with FKN or VC. (N) Cerebellar slices incubated with VC (top) or FKN (bottom) and immunostained with CC1 (red) and OLIG2 (green). Arrows indicate CC1^+^OLIG2^+^ cells. (O–R) Quantification of (N). n = 5–6 biological replicates. Data were analyzed using unpaired t test in (D) to (L), paired t test in (O) to (Q), and multiple t test in (C) and (R). Error bars represent SEM. ns, not significant. In all images tissue was counterstained with Hoechst (blue) to visualize nuclei. Scale bars, 50 μm (B), 20 μm (D, G, and J), and 100 μm (N). See also Figure S3.

To find out whether FKN promotes oligodendrogenesis *in vivo*, we infused FKN into the lateral ventricle of 3-month-old NestinCre^ERT2^;RosaYFP^STOP/+^ NPC lineage-tracing mice. NPCs and their progeny were labeled with YFP expression via tamoxifen injections, followed by intracerebral-ventricular (ICV) infusion of VC (BSA) or FKN (Figure 6A). We analyzed the corpus callosum area above the ventricle, which was further divided into the SVZ and white matter (WM) regions (Xing et al., 2014). FKN treatment caused a slight, but not significant, shift of YFP^+^ cells from the dorsal SVZ to the WM (Figure 6C).

To assess whether infusion of FKN caused a shift in SVZ NPC differentiation, we quantified the proportion of YFP^+^ cells that co-expressed OPC marker PDGFRα, early oligodendrocyte marker BCAS1 (Fard et al., 2017; Ishimoto et al., 2017), or mature oligodendrocyte marker CC1 (Bhat et al., 1996). FKN infusion led to a ∼2-fold increase in the proportion of YFP^+^PDGFRα^+^ OPCs in the SVZ and a ∼1.4-fold increase in the WM (Figures 6D–6F) when compared with VC. FKN had no effect on the proportion of newly formed YFP^+^BCAS1^+^ oligodendrocytes in the SVZ, but caused a significant ∼1.4-fold increase in *de novo* early oligodendrocytes in the WM (Figures 6G–6I). Similarly, FKN-infused animals showed a slight but non-significant increase in YFP^+^CC1^+^OLIG2^+^ cells in the SVZ and a statistically significant ∼1.4-fold increase in YFP^+^CC1^+^OLIG2^+^ oligodendrocytes in the WM (Figures 6J–6L). These data validate our *in vitro* findings and provide further evidence that FKN enhances the production of oligodendroglial lineage cells from SVZ NPCs.

### FKN increases *ex vivo* cerebellar OPC differentiation

To determine whether FKN can modulate oligodendrocyte formation in other brain areas, we incubated cerebellar slices with FKN or VC (PBS) (Figure 6M). While the postnatal cerebellum does not host NPCs, cerebellar slice cultures provide an *ex vivo* assay to assess developmental OPC differentiation and myelination (Shen and Yuen, 2020). To assess OPC differentiation, we analyzed deep cerebellar WM (Figure S3B, yellow box) immunostained with anti-OLIG2 and anti-CC1 (Figure 6N). FKN did not lead to changes in the total number of OLIG2^+^ cells (Figure 6O). However, there was a 28.55% ± 9.74% increase in CC1^+^OLIG2^+^ oligodendrocytes and a 26.62% ± 8.66% decrease in CC1^−^OLIG2^+^ OPCs and immature oligodendrocytes (Figures 6P and 6Q). To assess changes in OPC fates, we normalized data to the total number of OLIG2^+^ cells. This analysis showed that FKN increased the proportion of CC1^+^OLIG2^+^ cells, and decreased the proportion of CC1^−^OLIG2^+^ cells (Figure 6R). FKN treatment did not affect the relative proportion of GFAP signal (Figures S3B, S3D, and S3F), in line with our SVZ precursor *in vitro* data.

Next, we assessed the proportion of myelinated axons by analyzing the intersections of MBP^+^NF^+^ (neurofilament) to total NF^+^ axons with ten equidistant grids, as previously described (de Almeida et al., 2021) (Figure S3). In FKN-treated slices, there was a significant ∼20% increase in the proportion of myelinated axons compared with VC (Figures S3B, S3C, and S3E).

### Inhibition of FKN signaling reduces oligodendrocyte differentiation

**Figure 7:**
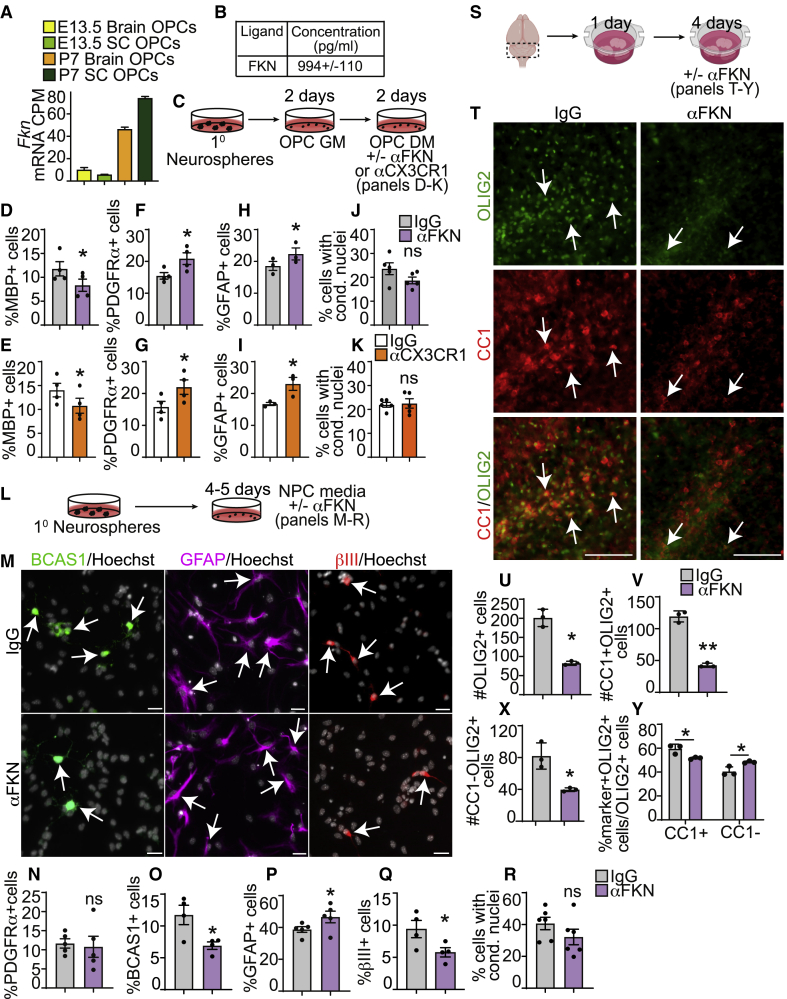
Inhibition of FKN signaling reduces oligodendrogenesis *in vitro* and *ex vivo* (A) *Fkn* mRNA expression in purified E13.5 and P7 murine OPCs extracted from RNA-seq analysis using Ki.se/en/mbb/oligointernode (Marques et al., 2018, GEO: GSE95093). (B) ELISA analysis of FKN in medium conditioned for 2–3 days by P7 SVZ OPCs, in pg/mL. n = 3 independent preparations. (C) Schematic: P7 SVZ primary neurosphere cells were cultured as OPCs in OPC GM for 2 DIV, followed by 2 DIV in OPC DM with function-blocking antibodies αFKN, αCX3CR1, or non-specific IgG. (D–K) Quantification of MBP^+^ (D and E), PDGFRα^+^ (F and G), or GFAP^+^ (H and I) cells, or cells with condensed nuclei (J and K) and cultured with αFKN (D, F, H, and J) or αCX3CR1 (E, G, I, and K). (L) Schematic: P7 SVZ primary neurosphere cells were cultured as adherent NPCs for 4–5 DIV with αFKN or IgG. (M). NPCs cultured with IgG (top) or αFKN (bottom) and immunostained with BCAS1 (green), GFAP (purple), or βIII (red), and counterstained with Hoechst (gray). Arrows indicate marker-positive cells. (N) Quantification of PDGFRα^+^ cells. (O–R) Quantification of (M). (S) Schematic: cerebellar slices from P10–P11 CD1 mice were cultured with αFKN or IgG. (T) Cerebellar slices cultured with IgG (left) and αFKN (right) and immunostained for OLIG2 (green) and CC1 (red). Arrows indicate CC1^+^OLIG2^+^ oligodendrocytes. (U–Y) Quantification of (T). All graphs were analyzed with paired t test except for (Y), which was analyzed with multiple t test. Data are mean ± SEM, n = 4–6. Scale bars, 20 μm (M) and 100 μm (T). See also Figure S4.

RNA-seq data from purified OPCs (Marques et al., 2018) show that P7 brain and spinal cord OPCs robustly express *Fkn* mRNA when compared with embryonic day 13.5 (E13.5) OPCs (Figure 7A). Enzyme-linked immunosorbent assay (ELISA) revealed that SVZ OPC-conditioned medium contained 994 ± 110 pg/mL FKN (Figure 7B).

To discover whether endogenous FKN regulates OPC fates, we cultured OPCs with a function-blocking antibodies specific to FKN (anti-FKN) or CX3CR1 (anti-CX3CR1), which are known to block FKN signaling *in vivo* and *in vitro* (Cipriani et al., 2011; Voronova et al., 2017). SVZ OPCs cultured in DM with anti-FKN or anti-CX3CR1 (Figure 7C) showed a statistically significant ∼25%–30% decrease in MBP^+^ oligodendrocytes (Figures 7D and 7E) and a concomitant 35%–40% increase in PDGFRα^+^ OPCs (Figures 7F and 7G) compared with isotype-matched immunoglobulin G (IgG). OPCs are known to differentiate into astrocytes (Suzuki et al., 2017). Our data indicated that anti-FKN and anti-CX3CR1 caused a 20%–37% increase in GFAP^+^ astrocytes when compared with IgG (Figures 7H and 7I).

We then asked whether the differences in OPC differentiation were due to changes in OPC proliferation. Analysis of OPCs in GM containing anti-FKN or anti-CX3CR1 (Figure S4A) did not support changes in the proportion of PDGFRα^+^ or Ki67^+^ cells or in the proliferative index of PDGFRα^+^ cells (Figures S4B–S4G). The observed effect on MBP^+^ and GFAP^+^ cell formation also appeared to not be due to changes in cell death, as the proportion of cells with condensed nuclei or CC3^+^ cells was unchanged (Figures 7J–7K and S4H–S4K).

To confirm the effect of FKN signaling inhibition on oligodendrocyte differentiation, we cultured P7 SVZ NPCs in NPC medium supplemented with anti-FKN or IgG for 4–5 days (Figure 7L). Anti-FKN treatment showed a statistically significant 48.57% ± 8.43% decrease in BCAS1^+^ oligodendrocytes (Figures 7M and 7O), 19.56% ± 4.38% increase in GFAP^+^ astrocytes (Figures 7M and 7P), 37.33% ± 5.13% decrease in βIII^+^ neurons (Figures 7M and 7Q), and no change in PDGFRα^+^ OPCs (Figure 7N) or cells with condensed nuclei (Figure 7R) when compared with IgG. Taken together, these results suggest that (1) OPCs are a source of FKN, (2) FKN signaling is an important regulator of SVZ precursor cell fate, and (3) inhibition of FKN signaling reduces oligodendrocyte and neuronal differentiation and possibly increases astrocyte formation.

### FKN signaling inhibition reduces *ex vivo* cerebellar oligodendrocyte genesis

Given that anti-FKN resulted in decreased oligodendrocyte formation from SVZ precursors *in vitro*, we then asked whether inhibition of FKN signaling could also impede oligodendrocyte genesis from precursors in other brain areas, such as the cerebellum. We treated cerebellar slices with anti-FKN or IgG for 4 days (Figures 7S and S4L). Anti-FKN treatment led to a statistically significant 58.53% ± 4.32% decrease in total OLIG2^+^ cells, a 64.04% ± 3.22% decrease in CC1^+^OLIG2^+^ oligodendrocytes, and a 49.99% ± 7.02% decrease in CC1^−^OLIG2^+^ cells (Figures 7T–7X). To find out whether anti-FKN changed the fate of OPCs, we presented the data as a proportion of total OLIG2^+^ cells. Similarly to our *in vitro* data, anti-FKN treatment resulted in a modest, but statistically significant 12.87% ± 3.76% decrease in the proportion of CC1^+^OLIG2^+^ cells and a 20.04% ± 6.81% increase in CC1^−^OLIG2^+^ cells (Figure 7Y). Additionally, there was a slight but non-significant increase in GFAP signal (Figures S4N and S4P). Finally, anti-FKN led to a ∼1.6-fold decrease in the proportion of myelinated MBP^+^NF^+^ axons (Figures S4M and S4O). Together, these data indicate that FKN signaling is important for appropriate cerebellar oligodendrocyte formation and potentially, myelination.

## Discussion

Our data demonstrate a novel role for FKN signaling in postnatal oligodendrogenesis from SVZ precursor cells, and suggest that FKN is a candidate molecule for engagement of CNS precursor cells for oligodendrocyte production and, possibly, remyelination.

The adult mammalian brain contains two stem cell niches, the SVZ and subgranular zone (SGZ). NPCs in both niches produce neurons and astrocytes throughout life. However, typically only SVZ NPCs are capable of producing oligodendrocytes (Obernier and Alvarez-Buylla, 2019). Intriguingly, infusion of exogenous FKN into the hippocampus increases SGZ neurogenesis in aged rats (Bachstetter et al., 2011) and rescues neurogenesis and hippocampal-dependent memory deficits in the BDNF^Met/Met^ mouse model of schizophrenia (Wang et al., 2014), implicating FKN's role in the activation and/or differentiation of NPCs. Within the SVZ, overexpression of soluble FKN in cells lining the lateral ventricle rescues cognitive function in an rTg450 tauopathy mouse model (Finneran et al., 2019). Our results extend these studies and show that infusion of FKN into the lateral ventricle increases SVZ oligodendrogenesis.

Previous reports suggest FKN can activate and enhance the survival of SVZ NPCs. Adult rodent SVZ NPCs respond to FKN *in vitro* via increased production of primary neurospheres (Silva-Vargas et al., 2016). Our results show that FKN does not alter the formation of secondary neurospheres from SVZ NPCs isolated from young rodents. This discrepancy may be attributed to analysis of secondary versus primary neurospheres or the age of the animals. Furthermore, human fetal NPCs express CX3CR1 and show increased cell survival in response to FKN when growth factors are depleted (Krathwohl and Kaiser, 2004). Our results support and extend these studies by demonstrating that SVZ NPCs and OPCs express CX3CR1 as well as bind and respond to FKN by increasing oligodendroglial cell formation without changes in proliferation or survival.

Our FKN-647 data suggest that CX3CR1 protein may be differentially expressed and localized to the surface of various CNS cells. For example, FKN-647 outlines the soma and processes in IBA1^+^ microglia but appears as puncta in SOX2^+^ NPCs. The pattern of FKN-647 binding to adult SVZ NPCs *in vivo* resembles EGF receptor immunostaining (Belenguer et al., 2021). Our *in vitro* data, however, show that FKN-647 outlines P7 SVZ NPC and OPC cell soma and processes. It is possible that cultured cells versus cells within tissue architecture, or young versus adult SVZ precursor cells, display different levels and patterns of CX3CR1 expression. Moreover, Mizrak et al. (2019) showed that activated (proliferating) SVZ NPCs have enriched *Cx3cr1* mRNA expression. Cultured NPCs are activated and highly proliferating, so they may display increased CX3CR1 expression. Together, our FKN-647 results align with the RNAscope data and previous reports (Ji et al., 2004; Meucci et al., 2000; Mizrak et al., 2019; Voronova et al., 2017; Wang et al., 2018) and confirm that SVZ NPCs and OPCs express *Cx3cr1* and bind FKN ligand.

Surprisingly, our results indicate that OPCs express and secrete FKN at a level comparable with neurons (Voronova et al., 2017), which were previously thought to be the primary source of FKN in the brain (Luo et al., 2019). This is similar to neuroligin-3, which is secreted by both neurons and OPCs (Venkatesh et al., 2017). When endogenous FKN is sequestered, SVZ OPCs and NPCs demonstrate decreased oligodendrogenesis and increased astrogliogenesis. These results support and extend our previous report demonstrating that inhibition of FKN signaling in embryonic cortical precursors results in decreased formation of oligodendrocytes (Voronova et al., 2017). Moreover, our data indicate that the CX3CR1 signaling pathway plays a direct role in postnatal SVZ NPC and OPC fate decisions. Intriguingly, CX3CR1^KO^ mice show hippocampal-dependent behavioral deficits and decreased neurogenesis (Xiao et al., 2015; Zhan et al., 2014). Our results similarly suggest that inhibition of FKN signaling reduces SVZ NPC neurogenesis, at least *in vitro*. Whether FKN-CX3CR1 signaling plays a direct role in SGZ NPC neurogenesis and/or hippocampal OPC oligodendrogenesis remains to be addressed.

While our *in vitro* experiments indicate that FKN directly regulates SVZ NPCs and OPCs for enhanced oligodendrocyte production, our *ex vivo* and *in vivo* results do not rule out the contribution of indirect actions via FKN signaling in microglia, which express high levels of CX3CR1 (Limatola and Ransohoff, 2014). Microglia play an important role during de- and remyelination; for example, suppression of microglial activation by minocycline does not affect SVZ oligodendrogenesis in a healthy SVZ niche, but drastically decreases OLIG2^+^ cells in a focally demyelinated SVZ (Naruse et al., 2018). Intriguingly, mice with a constitutive knockout of CX3CR1 exhibit more severe demyelination and neurological defects when subjected to cuprizone or experimental autoimmune encephalomyelitis (Garcia et al., 2013; Lampron et al., 2015). This was attributed to deficient phagocytosis of myelin debris by microglia and reduced OPC recruitment and proliferation in demyelinated lesions (Garcia et al., 2013; Lampron et al., 2015). It is tempting to speculate that at least some of these effects could be attributed to the effect of FKN on precursor cells.

Our work raises several questions in the field. Previous reports showed that pretreatment with soluble FKN prior to injury leads to neuroprotection in rodent models of stroke and Parkinson's disease as well as in an *ex vivo* model of demyelination (Cipriani et al., 2011; O'Sullivan and Dev, 2017; Pabon et al., 2011). It will be important to determine whether applying FKN after a demyelinating injury can elicit enhanced oligodendrogenesis and remyelination. A second key question involves the importance of OPC-secreted FKN in microglial function. It was recently shown that OPCs contribute to neuroinflammation and microglial activation in lipopolysaccharide-injected mice via CX3CR1 expressed on microglia (Zhang et al., 2019). Whether OPC-secreted FKN can modulate the CX3CR1 signaling pathway in microglia remains to be addressed. The third key question is whether CX3CR1 pathogenic variants detected in autism spectrum disorder, schizophrenia, and multiple sclerosis (reviewed in Watson et al., 2020) contribute to disease pathogenesis via aberrant FKN-CX3CR1 signaling in precursor cells.

In summary, we have discovered a new role for FKN signaling in regulating postnatal and adult SVZ oligodendrogenesis. Our findings support future investigations to test the ability of FKN to recruit CNS precursor cells for enhanced oligodendrocyte genesis in injured or diseased CNS, as well as the contribution of CX3CR1 variants to the development and progression of myelin-related deficits in neurodegenerative and neurodevelopmental disorders.

## Experimental procedures

### Contact for reagent and resource sharing

Further information and requests for resources and reagents should be directed to and fulfilled by the lead contact, Anastassia Voronova (voronova@ualberta.ca).

### Growth factors and function-blocking antibodies

Murine soluble FKN (R&D Systems) was added at 100, 250, and 500 ng/mL to secondary neurosphere assay; at 25, 100, 250, and 500 ng/mL to NPC monolayer cultures; and at 250 ng/mL to OPCs or cerebellar slice cultures. *In vivo*, 200 ng/day of FKN for multi-day infusion or 0.25–0.5 μg of FKN-647 (Almac) or BSA-647 (Thermo Fisher) for one-time delivery was infused into right lateral ventricle via ICV surgery. Anti-FKN (Torrey Pines Biolabs) and IgG (Jackson ImmunoResearch) controls were used at 20 μg/mL, and anti-CX3CR1 (Torrey Pines Biolabs) and IgG controls were used at 40 μg/mL.

### Experimental model and subject details

#### Mice

Animal use protocols were approved by the Research Ethics Office at the University of Alberta in accordance with the Canadian Council of Animal Care Policies. NestinCre^ERT2^ was obtained from Imayoshi et al. (2006), RosaYFP^STOP^ and C57BL/6J from Jackson Laboratories, CD1 from Charles River, and CX3CR1^KO^ from Cardona et al. (2018). In CX3CR1^KO^ mice, human CX3CR1^I249/M280^ RNA is expressed in murine Cx3cr1 locus, but is not translated until bred with Cre-recombinase-expressing mice (Cardona et al., 2018). Mice from both sexes were used for all experiments.

#### FKN infusion *in vivo* experiments

Two- to three-month-old WT or CX3CR1^KO^ mice were ICV-injected with FKN-647 or BSA-647 using coordinates listed below and euthanized 3 h post injection. Three-month-old NestinCre^ERT2^;RosaYFP^STOP/+^ mice were injected with tamoxifen (Sigma) for 5 days. Seventy-two hours later, ICV surgery was performed using coordinates −1.000 mediolateral, −0.300 anterior-posterior, −2.500 dorsoventral relative to bregma. FKN was infused for 7 days using osmotic mini-pumps (Alzet, 1007D).

### Primary cultures

#### Neurospheres

SVZ was microdissected from P1–P2 or P7 CD1 pups and cultured at clonal density as primary neurospheres for 6DIV and secondary neurospheres for 7 DIV as described in Storer et al. (2018).

#### NPC monolayer cultures

Dissociated primary neurosphere cells were cultured for 1–5 DIV on coverslips coated with poly-D-lysine (Sigma) and laminin (VWR) in medium supplemented with 2% B27 (Invitrogen), 10 ng/mL FGF, and 20 ng/mL EGF (Peprotech). BrdU (3 μg/mL; Sigma) was added for 2 h before fixation.

#### OPC cultures

Dissociated primary neurosphere cells were seeded as described above in medium supplemented with 2% B27, 10 ng/mL FGF, and 10 ng/mL PDGF-AA (R&D) and cultured for 2–3 DIV. Medium conditioned by OPCs was collected and subjected to CX3CL1 ELISA (RayBiotech) (n = 3 preparations). Differentiation was induced by replacing with medium supplemented with 2% B27 and 40 ng/mL 3,3′,5-triiodo-L-thyronine (T3; Sigma).

#### OPC-cortical neuron co-cultures

Cells isolated from P1–P2 cortices were seeded in Neurobasal Plus medium supplemented with 2% B27 plus (Invitrogen). Cortical neurons were enriched by wiping out proliferating cells with arabinocytoside C (AraC; Sigma) incubation for 48 h. Neurons were cultured without AraC for an additional 4 days, at which point SVZ OPCs were seeded onto neurons and cultured for 2–6 DIV in the presence of 40 ng/mL T3 and FKN or VC.

#### Organotypic cerebellar cultures

Cerebellar slices (300 μm) isolated from P10–P11 CD1 pups were cultured using an interface method (de Almeida et al., 2021) whereby FKN/VC or anti-FKN/IgG were added at 1 DIV for 4 days.

### Reagents and immunostaining

#### Immunocytochemistry and immunohistochemistry

Cell cultures were fixed with 4% paraformaldehyde (PFA) and subjected to FKN-647 binding assay without cell permeabilization and/or immunocytochemistry (ICC) with cell permeabilization. Mice over 21 days of age were transcardially perfused with Hank’s balanced salt solution followed by 4% PFA. Mice under 21 days of age were euthanized with CO_2_, and dissected brains were fixed in 4% PFA for 16–24 h. Cryosections (18 μm) were rehydrated and subjected to immunohistochemistry (IHC). Cultured cerebellar slices were fixed with 4% PFA for 1 h, followed by permeabilization and IHC. Detailed ICC and IHC protocols as well as primary and secondary antibodies are listed in supplemental information.

#### RNAscope

RNAscope was performed using cryosections from P2, P7, P15, and P65 CD1 brains as described in Voronova et al. (2017) with probes targeting murine *Cx3cr1*, *Olig2*, *Sox2* mRNA, or negative control probe purchased from ACD according to the manufacturer's instructions. Cells with three or more RNAscope dots were considered to be positive for marker expression.

#### Microscopy

RNAscope sections were imaged with a Zeiss LSM700 confocal microscope with z-stacks and an optical slice thickness of 0.2–0.5 μm. All other images were captured using a Zeiss Axio Imager M2 fluorescence microscope. Image acquisition was performed with Zen software (Zeiss). Cultured cells and *in vivo* images were imaged in single plane or with z-stacks and optical slice thickness of 0.5–1 μm.

### Quantification and statistical analysis

*In vitro* and *ex vivo* data are from at least three independent biological experiments. At least 500–2,000 cells per treatment and biological experiment were counted. For *in vivo* experiments, 5–8 anatomically matched sections per brain were analyzed from six mice across three independent litters unless indicated otherwise. All data are presented as mean ± SEM. For two-group comparisons, two-tailed paired (*in vitro* and *ex vivo* datasets) or unpaired Student's or multiple t tests (*in vivo* datasets) were used to assess statistical significance between means. For three or more group comparisons, one-way ANOVA was followed by Dunnett's multiple comparisons test. A p value of <0.05 was considered significant (^∗^p < 0.05, ^∗∗^p < 0.01, ^∗∗∗^p < 0.001 in figures).

## Author contributions

Conceptualization, A.E.S.W. and A.V.; Methodology, A.E.S.W., M.M.d.A., T.F., A.V., G.V., D.G., and A.E.C.; Formal analysis, A.E.S.W., Y.L., M.M.d.A., N.L.D., P.T., and A.V.; Investigation, A.E.S.W., Y.L., M.M.d.A., N.L.D., P.T., T.F., D.G., and A.V.; Resources, A.V. and S.S.; Writing – original draft, A.E.S.W. and A.V.; Writing – review & editing, A.E.S.W. and A.V.; Supervision, S.S. and A.V.; Funding acquisition, A.V.

## Declaration of interests

The authors declare no competing interests.

## Supplemental information

Document S1. Supplemental experimental procedures and Figures S1–S4

Document S2. Article plus supplemental information
