# Supplementary material for: Fractalkine signaling regulates oligodendroglial cell genesis from SVZ precursor cells
Source: Stem Cell Reports. 2021 Jul 15;16(8):1968–84. doi: 10.1016/j.stemcr.2021.06.010 (PMC8365111; doi:10.1016/j.stemcr.2021.06.010)
Supplement: Document S1. Supplemental experimental procedures and Figures S1–S4 [file mmc1.pdf]

Stem Cell Reports, Volume *16*

Supplemental Information

Fractalkine signaling regulates oligodendroglial cell genesis from SVZ precursor cells

Adrianne E.S. Watson, Monique M.A. de Almeida, Nicole L. Dittmann, Yutong Li, Pouria Torabi, Tim Footz, Gisella Vetere, Danny Galleguillos, Simonetta Sipione, Astrid E. Cardona, and Anastassia Voronova

Fig. S1, related to Fig. 1

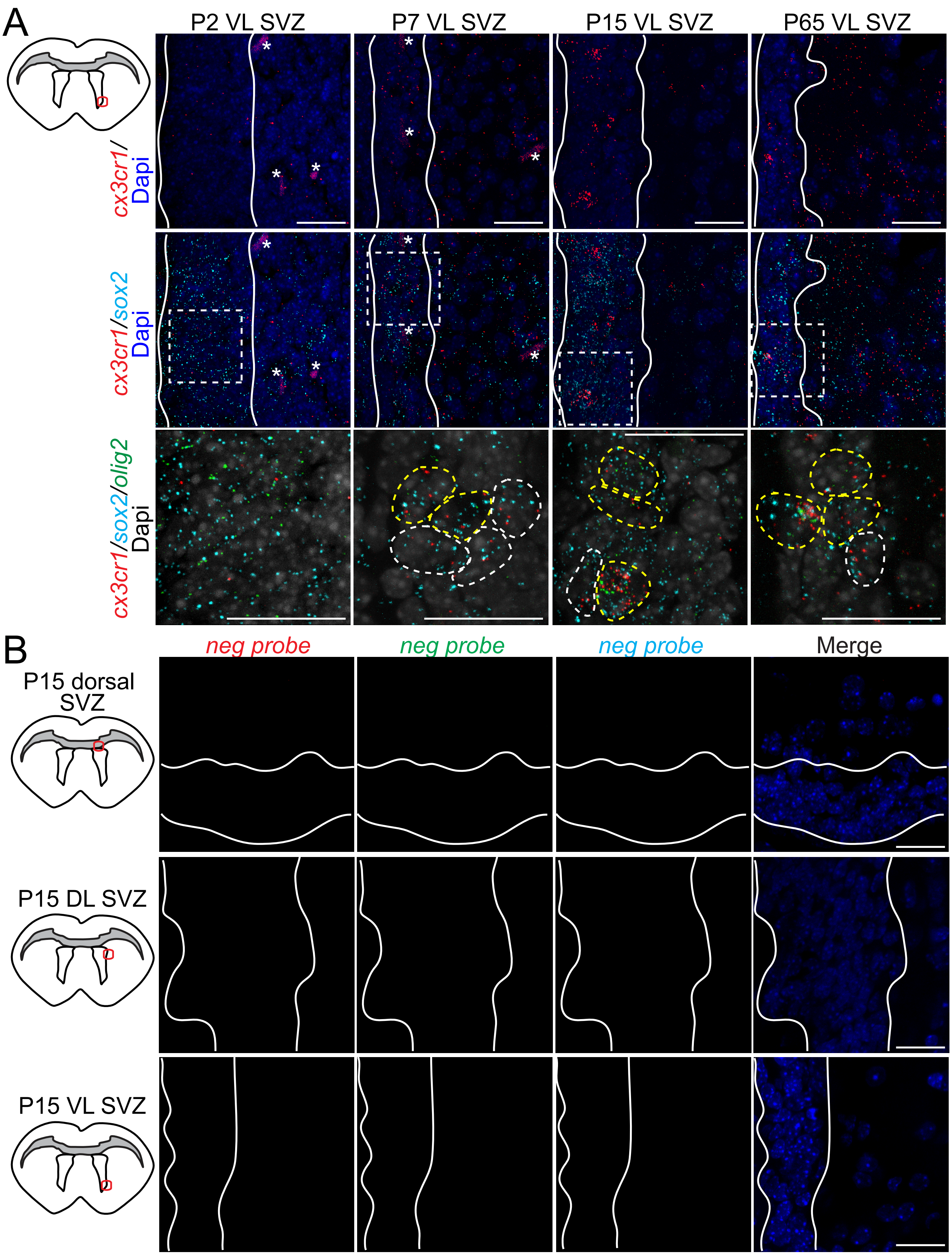

Figure S1. NPCs and OPCs express *Cx3cr1* mRNA in the ventrolateral SVZ. Related to Fig. 1. A. Top left: Schematic of coronal brain section, approximate location of ventrolateral (VL) SVZ indicated by red circle. Top row: RNA Scope analysis of VL SVZ from P2, P7, P15, and P65 for *Cx3cr1* (red) mRNA. Middle row: RNA Scope analysis of VL SVZ from P2, P7, P15, and P65 (left-right) for *Cx3cr1* (red) and *Sox2* (cyan) mRNAs. Hatched boxes indicate section shown in higher magnification in bottom row. Bottom row: Higher magnification of hatched boxes in middle rows. RNA scope analysis of VL SVZ from P2, P7, P15, and P65 for *Cx3cr1* (red), *Sox2* (cyan), and *Olig2* (green) mRNAs. White dashed circles indicate $Cx3cr1^{+}SOX2^{+}$ cells, and yellow dashed circles $Cx3cr1^{+}Sox2^{+}Olig2^{+}$ cells. **B**. RNA Scope analysis of P15 dorsal (top), dorsolateral (DL, middle), and ventrolateral (VL, bottom) SVZ from P15 mice with negative probe. Left: schematic of coronal brain section with approximate location captured, indicated by red circle. For all images cells were counterstained with Dapi (blue or grey). Solid lines indicate SVZ boundaries. Asterisks denote blood vessels. Scale bars are 20μm. A cell was considered positive for *Cx3cr1*, *Sox2* and/or *Olig2* if it contained at least 3 RNA Scope signal dots for respective mRNA. n=2-3 for each age.

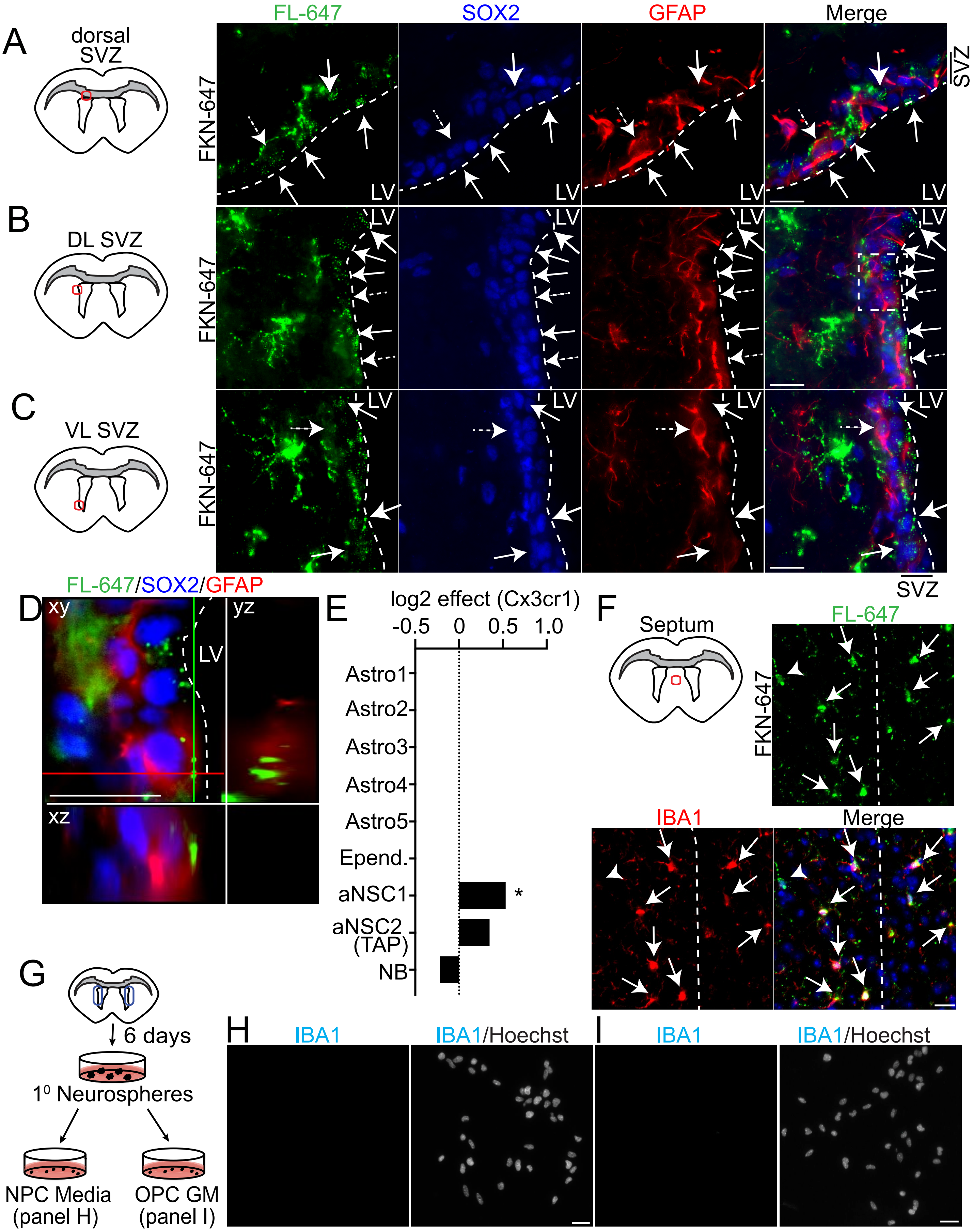
FL-647
SOX2
GFAP
Merge
A
dorsal SVZ
FKN-647
LV
SVZ
B
DL SVZ
C
VL SVZ
D
FL-647/SOX2/GFAP
xy
yz
xz
E
log2 effect (Cx3cr1)
-0.5
0
0.5
1.0
Astro1
Astro2
Astro3
Astro4
Astro5
Epend.
aNSC1
*
aNSC2 (TAP)
NB
F
Septum
IBA1
G
6 days
1⁰ Neurospheres
NPC Media (panel H)
OPC GM (panel I)
H
IBA1
IBA1/Hoechst
I

Figure S2. FKN binds SVZ NPCs *in vivo*. Related to Fig. 2. A-C. 2-3-month old WT mice were injected once with FKN-647 into the right lateral ventricle via ICV surgery and euthanized 3-hours post injection. **A-C.** Left: schematic of coronal section showing approximate location of dorsal **(A)**, dorsolateral (DL) **(B)** and ventrolateral (VL) **(C)** SVZ is indicated by red circle. Sections were immunostained with NPC markers SOX2 (blue, second column) and GFAP (red, third column). Fluorescence in far-red channel (FL-647) is pseudo-colored in green (left column). Solid arrows indicate FKN-647^{+}SOX2^{+}GFAP^{+} cells. Dashed arrows indicate FKN-647^{+} SOX2^{+}GFAP^{-} cells. Dashed line indicates SVZ boundary and LV indicates lateral ventricle. Hatched box in "merge" panel in **B** is shown at higher magnification in **D**. **D.** Orthogonal slice through Z-stack of FKN-647^{+}SOX2^{+}GFAP^{+} cells in DL SVZ demonstrating FKN-647 signal is detected on the surface of NPCs. On xy projection (left), green line indicates location of yz plane projection (right). Red line indicates location of xz projection (bottom). Dashed line indicates SVZ boundary and LV indicates lateral ventricle. **E.** *Cx3cr1* mRNA expression enrichment in SVZ cell subclusters. Data was extracted from single cell RNA sequencing binomial specific analysis ([1] and Table S2 within). * FDR <0.05. Astro = non-proliferating quiescent SVZ neural stem cell (NSC), Epend. = ependymal, aNSC = proliferating activated NSC, TAP = transit amplifying progenitor, NB = neuroblast. **F.** Top left corner: schematic of coronal section showing approximate location of septum is indicated by red circle. Top right corner and bottom row: representative image of FKN-647 infused WT mouse immunostained with IBA1 (red). Fluorescence in far-red channel (FL-647) is pseudo-colored in green. Solid arrows indicate FKN-647^{+}IBA1^{+} cells. Arrowhead indicates FKN-647^{+}IBA1^{-} cell. Sections were counterstained with Hoechst 33258 (blue). **G.** Schematic illustration of SVZ NPC and OPC cultures. **H-I.** Neurosphere cells were cultured in NPC media for 1 DIV **(H)** or OPC GM for 2 DIV **(I)**, immunostained for IBA1 (cyan), and counterstained with Hoechst 33258 (grey). Scale bars are 20 μm.

Fig. S3
related to Fig. 6

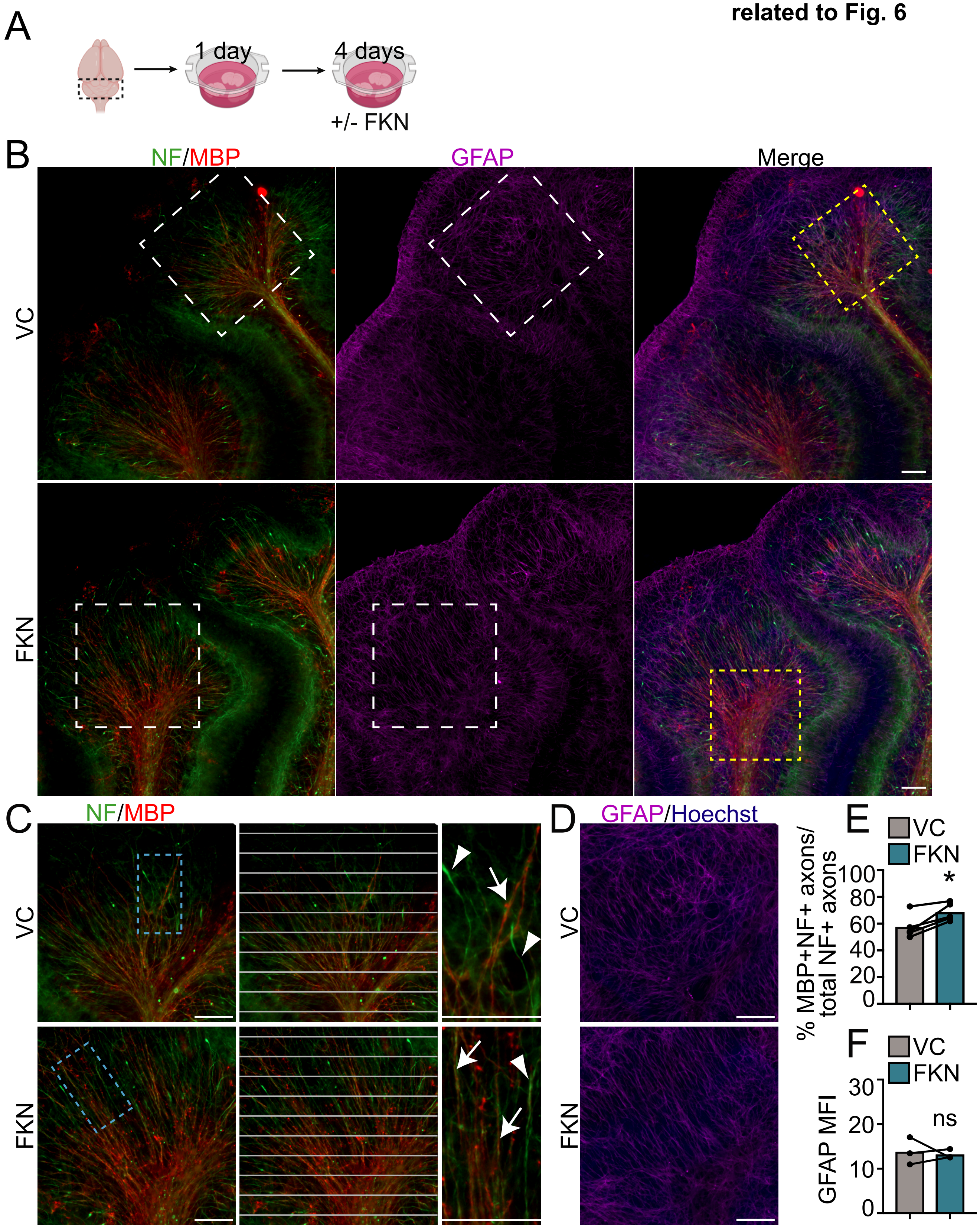

Figure S3: FKN increases proportion of myelinated axons in cerebellar *ex vivo* slices. Related to Fig. 6. A. Schematic: cerebellar slices from P10-11 CD1 mice were allowed to adhere in culture for 1 day, followed by 4-day incubation with 250 ng/ml FKN or VC (PBS). **B.** Representative image of cerebellar slice cultured with VC (top) or FKN (bottom) and immunostained for neurofilament (NF, green), MBP (red), and GFAP (purple). Nuclei were visualized with Hoechst (blue in Merge panels). Hatched white boxes are shown in higher magnification in **C.** Hatched yellow boxes in Merge panels are representative of deep white cerebellar white matter analyzed in Fig. 6M-R. **C**. Higher magnification of cerebellar slice cultured with VC (top) or FKN (bottom) and immunostained for NF (green) and MBP (red). Hatched blue boxes in left column are shown at higher magnification on right. Middle column: lines indicate grid used to quantify MBP^+NF^+ and total NF^+ fibre intersections with each grid (used to calculate proportion of myelinated axons relative to total axons). Right column: higher magnification of left column. Arrows indicate MBP^+NF^+ axons, and arrowheads indicate MBP^-NF^+ axons. **D.** Higher magnification of cerebellar slice cultured with VC (top) or FKN (bottom) and immunostained for GFAP (purple) and counterstained with Hoechst 33258 (blue). **E.** Quantification of **C**. *$p<0.05$. n=5 biological replicates with at least 2 slices analyzed per replicate. At least 600 NF intersections were counted for each replicate. **F.** Quantification of mean fluorescent intensity (MFI) in **D.** ns=not significant. n=3 biological replicates with at least 3 slices analyzed per replicate. All scale bars: 100μm. Graphs were analyzed with student's paired t-test.

Fig. S4
related to Fig. 7

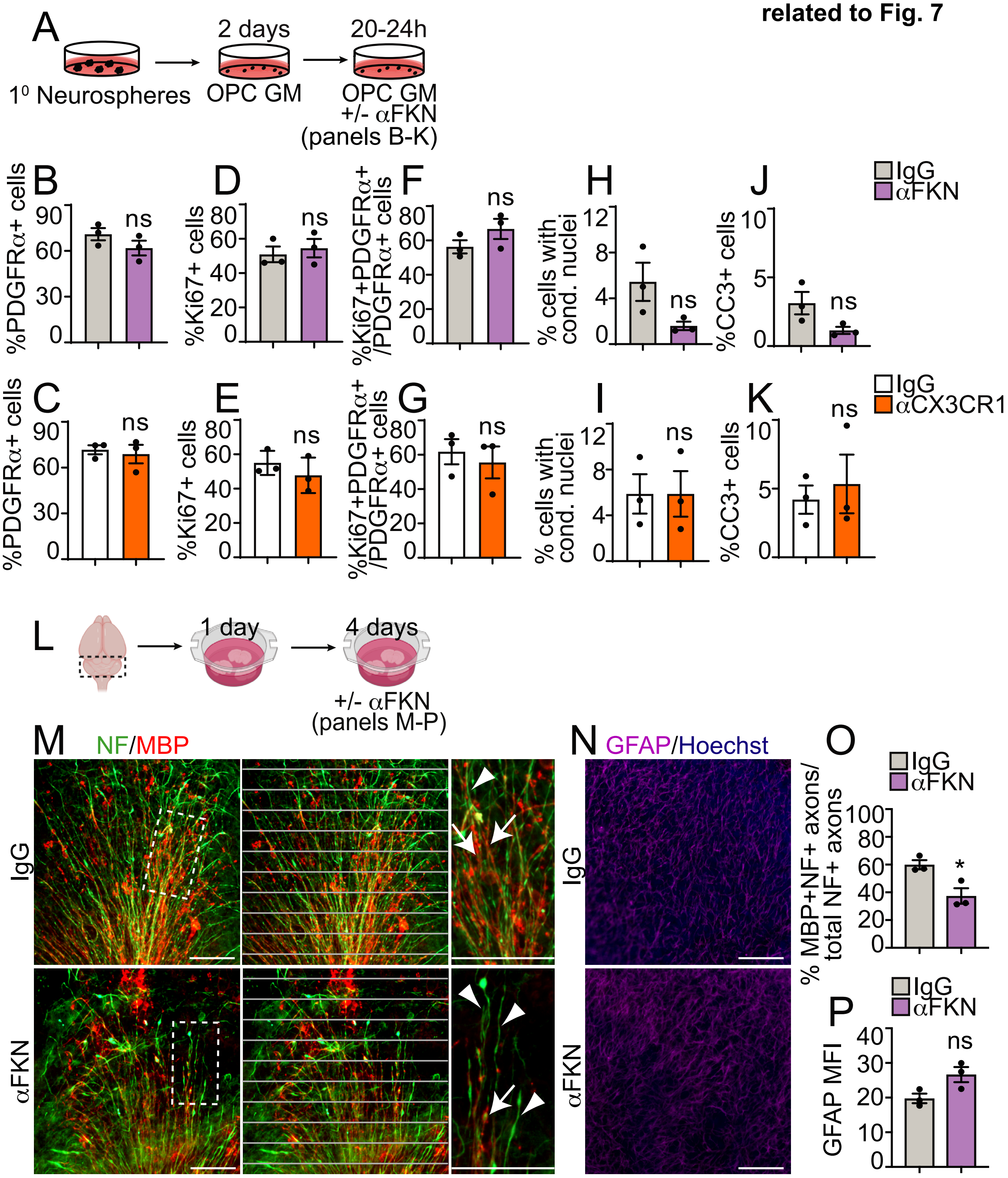

Figure S4. Inhibition of FKN signaling does not affect OPC proliferation, but reduces proportion of myelinated axons in cerebellar *ex vivo* slices. Related to Fig. 7. A. Schematic: P7 SVZ primary neurosphere cells were cultured as monolayers in OPC GM for 2DIV, followed by 20-24 hours in OPC GM with αFKN (20 μg/ml), αCX3CR1 (40 μg/ml) or IgG (20 or 40 μg/ml). **B-K.** Quantification of PDGFRα$^+$ cells **(B-C)**, Ki67$^+$ cells **(D-E)**, proliferation index of PDGFRα$^+$ cells **(F-G)**, cells with condensed nuclei **(H-I)**, and CC3$^+$ cells **(J-K)** cultured with αFKN (**D, D, F, H, J**) or αCX3CR1 (**C, E, G, I, K**). Marker$^+$ cells were expressed as % of healthy Hoechst$^+$ cells. Proliferation index is expressed as %Ki67$^+$marker$^+$ cells relative to total marker$^+$ cells. Cells with condensed nuclei expressed as % of total Hoechst$^+$ cells **L.** Schematic: cerebellar slices from P10-11 CD1 mice were allowed to adhere in culture for 1 day, followed by a 4-day incubation with αFKN or IgG (20 μg/ml). **M.** Representative image of cerebellar slice cultured with IgG (top) or αFKN (bottom) and immunostained for NF (green) and MBP (red). Hatched boxes in left column are shown in higher magnification on right. Middle column: lines indicate grid used to quantify MBP$^+$NF$^+$ and total NF$^+$ fibre intersections with each grid (used to calculate proportion of myelinated axons relative to total axons). Right column: higher magnification of left column. Arrows indicate MBP$^+$NF$^+$ axons, and arrowheads indicate MBP$^-$NF$^+$ axons. **N.** Representative image of cerebellar slice cultured with VC (top) or FKN (bottom) immunostained for GFAP (purple) and counterstained with Hoechst 33258 (blue). **O.** Quantification of **M**. **P.** Quantification of mean fluorescent intensity (MFI) in **N.** All scale bars: 100 μm. Graphs were analyzed with student's paired t-test. * $p<0.05$, ns=not significant. n=3 biological replicates.

Supplemental Experimental Procedures

MATERIALS AND METHODS

Growth factors and function-blocking antibodies: Murine soluble FKN (CX3CL1) was obtained from R&D Systems (cat #458-MF), FKN-647 from Almac (cat #CAF-51-A-01, #CAF-14-A-03). BSA-647 was obtained from ThermoFisher (cat# A34785). Cy5-Streptavidin was obtained from Jackson ImmunoResearch (cat# 016-170-084). For cell and slice culture experiments, FKN was reconstituted in sterile 1X PBS at a concentration of 100 μg/mL. FKN was added at 100, 250 and 500 ng/ml to secondary neurosphere assay; at 25, 100, 250 and 500 ng/ml to NPC monolayer cultures (please see "Primary cell and *ex vivo* cerebellar slice cultures" section below). FKN was added at 250 ng/ml to OPCs cultured in OPC growth (GM) or differentiation (DM) media or to cerebellar slice cultures. For FKN intracerebral ventricular (ICV) surgery, FKN was reconstituted at 16.7 ng/mL in sterile 0.2% bovine serum albumin (BSA, Jackson ImmunoResearch) in 1x PBS. FKN-647 was reconstituted at 0.5 mg/mL in 0.1% BSA in 1X PBS. Function-blocking antibody specific for FKN (cat #TP233) and CX3CR1 (cat #TP501) was obtained from Torrey Pines Biolabs. Non-specific rabbit IgG was obtained from Jackson ImmunoResearch. Anti-FKN and IgG control were used at 20 μg/ml and anti-CX3CR1 and IgG control were used at 40 μg/ml as per (2). Anti-FKN and anti-CX3CR1 are validated to block FKN signalling as per (2-5).

Experimental Model And Subject Details

Mice: Animal use protocols were approved by the Research Ethics Office at the University of Alberta in accordance with the Canadian Council of Animal Care Policies. Mice from both sexes were used for all cell culture and *in vivo* experiments. NestinCre^{ERT2} (C57BL/6-Tg(Nes-cre/ERT2)) was obtained from (6). RosaYFP^{STOP} (B6.129X1-Gt(ROSA)26Sortm1(EYFP)Cos/J; RRID:IMSR_JAX:006148) and wild-type (WT) C57BL/6J mice were obtained from Jackson Laboratories. CX3CR1 knockout (KO) mice, in which human CX3CR1 with I249/M280 mutation was knocked into mouse CX3CR1 locus (hCX3CR1$^{I249/M280fl/fl}$) were obtained from (7). In these mice, hCX3CR1$^{I249/M280}$ RNA is expressed in murine Cx3cr1 locus, but is not translated until bred with Cre-recombinase mice (7). For primary cell culture experiments, CX3CR1^{KO} (7) or CD1 mice purchased from Charles River Laboratory were used for all primary cell culture experiments (developmental age: postnatal day [P] 1-2, 7, slice culture [P10-11]). For RNA scope, P2, P7, P15. and P65 mice were used. For NPC lineage tracing experiments and FKN-647 infusion, 2-3-month old animals were used.

For genotyping, the following primers were used: NestinCre^{ERT2}: TTCCGCTGGGTCACTGTCGCCGCTAC (5'-3', forward), TAATCGCGAACATCTTCAGGTTCTGC (5'-3', reverse), RosaYFP^{STOP}: AAAGTCGCTCTGAGTTGTTAT (5'-3', common), GGAGCGGGAGAAATGGATATG (5'-3', wildtype), GGAGCGGGAGAAATGGATATG, (5'-3', mutant), CX3CR1^{KO}: TACCTGGCCATCGTCCTGGCCGCCA (5'-3', forward), ACGAGACTAGTGAGACGTGCTACTT (5'-3', reverse); CX3CR1^{WT}: GTCTTCACGTTCGGTCTGGT (5'-3', forward), CCCAGACACTCGTTGTCCTT (5'-3', reverse).

FKN infusion *in vivo* experiments: For FKN-647 infusion experiments (Fig. 2A-G, Fig. S2A-D, F), 2-3-month old wild-type C57/BL6J or CX3CR1^{KO} mice were used. For FKN mini-pump infusion experiments (Fig. 6A-L), NestinCre^{ERT2}-positive males were bred with RosaYFP$^{STOP/STOP}$-positive females. Resulting Cre-positive, RosaYFP$^{STOP/+}$ 3-month old progeny were injected with 3 mg tamoxifen dissolved in 10% ethanol (Commercial Alcohols) and 90% sunflower seed oil (Sigma) daily for 5 days. 72h after the last tamoxifen injection, ICV surgery was performed. For ICV surgery, mice were anesthetized via inhalation of isoflurane and placed in a stereotaxic frame. Syringe needle (for one-time injection) or cannulas (Alzet, for multi-day infusion) were positioned after craniotomy using the following coordinates relative to bregma: -1.000 medio-lateral, -0.300 anterior-posterior, -2.500 dorso-ventral for infusion into right ventricle. For one-time injection, 0.5-1 μl of FKN-647 or matched volume and equimolar amount of BSA-647 or Cy5-Streptavidin were injected once over a 10-20 min period. For multi-day infusion, 7-day osmotic mini-pumps (Alzet, 1007D) were connected containing VC or 16.7 ng/ml FKN. Overall, 200 ng of FKN was delivered over 24h for 7 days. Cannula was secured to the skull with Loctite 454. After 3h (with one-time injection) or 7 days (with mini-pump infusions), mice were anesthetized with 102 mg/kg of body weight Euthansol (Western Drug Distribution Center Limited, WDDC). Mice were then perfused transcardially with Hank's Balanced Salt Solution (HBSS, Gibco), followed by 4% paraformaldehyde in 1X PBS (PFA, Acros Organics). Brains were post-fixed in 4% PFA for additional 24 hours, after which brains

were cryopreserved in 30% sucrose (Fisher) in 1X PBS for 72 hours. Brains were embedded in optimal cutting temperature (O.C.T) compound (Thermo Scientific™ Shandon™ Cryomatrix™) and flash frozen for downstream assays.

Primary cell and ex vivo cerebellar slice cultures:

Primary Neurospheres: Subventricular zone (SVZ) tissue was microdissected from P1-2 or P7 CD1 pups of either sex. Primary neurospheres were cultured as described in (8) at 10 cells/µL. Briefly, cells were cultured in Serum-Free Media (SFM; Dulbecco's Modified Eagle Medium (low glucose) (DMEM, Gibco), Ham's F-12 Nutrient Mixture (F12, Gibco), 0.6% glucose (Sigma), 0.1125% Sodium Bicarbonate ($NaHCO_3$, Gibco), 5 mM N-2-hydroxyethylpiperazine-N-2-ethane sulfonic acid (HEPES, Gibco), 100 µg/mL L-glutamine (Lonza), 1% Penicillin-Streptomycin (Pen/Strep, Lonza) supplemented with 2% B27 supplement (Life Technologies), 10 ng/ml Fibroblast Growth Factor (FGF, Peprotech), 20 ng/ml Epidermal Growth Factor (EGF, Peprotech), and 2 µg/ml heparin sodium salt (Sigma) (herein referred to as **Neurosphere Media**). After 6 days, neurospheres were collected, dissociated, and plated for downstream assays as outlined below.

Secondary Neurospheres: Dissociated primary neurosphere cells cultured from P7 SVZ were seeded at clonal density of 2 cells/µL in Neurosphere Media for 7 days. Each sphere containing at least 50 cells was counted on 7DIV (days *in vitro*) as per (9).

NPC Monolayer Cultures: Dissociated primary neurosphere cells were seeded at 39,500 cells/cm^2 on plates coated with 40 µg/ml Poly-D-Lysine (Sigma) and 4 µg/ml laminin (Corning) in SFM supplemented with 2% B27, 10 ng/ml FGF and 20 ng/ml EGF and cultured for 1-5DIV. For proliferation assays, BrdU (Bromodeoxyuridine [5-bromo-2'-deoxyuridine], Sigma) was added at 3 µg/ml for 2h before fixing the cultures.

OPC Cultures: Dissociated primary neurosphere cells were seeded at 39,500-47,000 cells/cm^2 on plates coated with Poly-D-Lysine and laminin in SFM supplemented with 2% B27, 10 ng/ml FGF and 10 ng/ml Platelet Derived Growth Factor AA (PDGF-AA, R&D) (herein referred to **OPC growth media [GM]**) and cultured for 2-3DIV. To induce differentiation, media was changed on 2-3 DIV to SFM supplemented with 2% B27 and 40 ng/ml 3,3',5-Triiodo-L-thyronine (T3, Sigma) (herein referred to **OPC differentiation media [DM]**) and cells were cultured for additional 2-5 days (10).

OPC-Cortical Neuron Co-Cultures:

Cortical Neuron Cultures: Pia layer was removed from P1-P2 brains and cortical tissue from P1-P2 pups was microdissected in Earle's Balanced Salt Solution (EBSS, Gibco) and digested with papain (Worthington) in EBSS. Reaction was stopped with fetal bovine serum (FBS, Invitrogen). Tissue was washed and mechanically dissociated in 10% FBS in DMEM (high glucose, Gibco) with 1% Pen/Strep. Dissociated neurons were seeded at 105,000 cells/cm^2 on plates coated with 40 µg/ml Poly-D-Lysine and 4 µg/ml laminin. 2-4h after plating, media was exchanged to Neurobasal Plus (Invitrogen) with 100 µg/mL L-glutamine, 1% Pen/Strep supplemented with 2% B27 plus supplement (Life Technologies, **Neuron Media I**). After 2 days, wipeout of proliferating, non-neuronal cells was performed by adding fresh **Neuron Media I** with 3 μM Arabinocytoside C (AraC, Sigma) for 48h. AraC and dead cells were washed out with 3 changes of Neurobasal (Invitrogen) with 100 µg/mL L-glutamine, 1% Pen/Strep supplemented with 2% B27 supplement (**Neuron Media II**). Neurons were cultured for additional 4 days, with **Neuron Media II** exchanged every 48h. At this timepoint, cultures contained neurons (50%) and astrocytes (50%) with no microglia or oligodendrocytes (data not shown).

Co-Cultures: OPC cultures were generated from SVZ primary neurospheres as described above from the same P1-P2 pups that were used to generate cortical neuron cultures. On day of co-culture, SVZ OPCs were lifted with 0.025% trypsin (Invitrogen), washed, and resuspended in **Neuron Media II** with 40 ng/ml T3. OPCs were added to neurons at 105,000 cells/cm^2 in the presence of 250 ng/ml FKN or VC (PBS). OPCs were co-cultured with neurons for 2-6 days, where 50% media was replaced every 48h.

Conditioned medium preparation/ELISA: OPCs were cultured in GM as above for 2-3DIV. Conditioned medium was collected and centrifuged at 500g for 7 min to remove dead cells and debris. FKN protein detection was performed via enzyme-linked immunosorbent assay (ELISA) kit (RayBiotech) following

manufacturer's directions. ELISA measurements were performed via technical duplicates from 3 independent preparations of OPC conditioned medium.

Organotypic cerebellar cultures: Developmental myelination was analyzed by using an *ex vivo* organotypic cerebellar slice culture model as previously described (11-13). Briefly, cerebellum from P10-11 CD1 mice was dissected into HBSS ice-cold solution containing 10 mM glucose, 10 mM $MgCl_2$ (Fisher Scientific), 2 mM $CaCl_2$ (Sigma) and 0.1% Pen/Strep. Cerebellar parasagittal slices were cut at 300 μm thickness by using an oscillating tissue slicer (OTS-5000, EMS). Slices were then transferred to a membrane insert (Millipore, 30 mm diameter, pore size 0.4 μm) and cultured using an interface method, with 1 ml of serum-based medium composed of 50% Dulbecco's Modified Eagle Medium with Glutamax-1 (DMEM+Glutamax, Gibco), 24% Earle's Balanced Salt Solution containing calcium, magnesium and phenol red (EBSS, Gibco), D-glucose (0.13 mg/mL, Sigma), 1% Pen/Strep, and 25% horse serum (Gibco). Slices were maintained at 37°C, and kept under 95% O_2 and 5% CO_2 for one day. At this timepoint, medium was removed and replenished with medium containing PBS or IgG (control medium) and 250 ng/mL FKN or 20 μg/mL function blocking antibody specific for murine FKN (please see "Growth factors and function-blocking antibodies" section). ½ media re-supplemented with PBS, IgG, FKN or anti-FKN was exchanged every 2 days. Slices were fixed in 4% PFA after 96h of treatment.

<u>Reagents and Immunostaining:</u>

Immunocytochemistry (ICC): With the exception of cell cultures used for FKN-647 binding assay, cell cultures were fixed with 4% PFA (ACROS Organics) for 10 minutes at room temperature, permeabilized with 0.2% NP-40 (Sigma) for 5 min, and blocked with 6% donkey serum (Jackson ImmunoResearch) in 0.5% BSA in 1X PBS (ICC blocking buffer). Primary antibodies listed in "Antibodies" section were added in ½ ICC blocking buffer and ½ PBS for 2 hours at room temperature or overnight at 4°C. Appropriate secondary antibodies listed in "Antibodies" section were added in 1X PBS for 1 hour at room temperature. For BrdU immunostaining, after incubation with the fluorescently labelled secondary antibodies, cells were washed 3 times with 1X PBS, post-fixed for 10 min with 4% PFA for 10 min at room temperature, washed with 1X PBS, incubated with 1M hydrochloric acid (HCl, Fisher) for 10 min at 4°C and 2M HCl for 20 min at room temperature. After extensive washes with 1X PBS, slides were blocked with 5% donkey serum, 1M glycine (Sigma), and 1% Triton X-100 (Bio Basic) in 1X PBS for 30 minutes at room temperature, then incubated with antibodies specific for BrdU (please see "Antibodies" section) in 1X PBS for 1h at room temperature or overnight at 4°C followed by appropriate secondary antibody for 1h at room temperature. To visualize nuclei, cultures were counter-stained with Hoechst 33258 (Riodel-De Haen Ag) for 2 min. Slides were mounted with Fluoromount G (ThermoFisher).

For FKN-647 *in vitro* binding assay, cell cultures were fixed with 4% PFA for 10 min, and blocked with ICC blocking buffer without permeabilization. FKN-647 or Streptavidin-Cy5 (Jackson ImmunoResearch) at equimolar amounts in ½ ICC block and ½ 1X PBS were added to cell cultures for 2 hours at room temperature or 4°C overnight. Cell cultures were mounted with Fluoromount G or processed for additional antibody staining. In this case, FKN-647 or Cy5-streptavidin incubated cultures were washed with 1X PBS, post-fixed for 10 min with 4% PFA for 10 min at room temperature, washed with 1X PBS, permeabilized and blocked as described above, and incubated with primary antibodies listed in "Antibodies" section Appropriate secondary antibodies conjugated to Alexa555/Cy3 or Alexa488/FITC were used to visualize the signal.

Immunohistochemistry (IHC): Mice over 21 days of age were transcardially perfused with HBSS (Gibco) followed by 4% PFA (Acros Organics) and post-fixed in 4% PFA for additional 24h at 4°C. Mice under 21 days of age were euthanized with CO_2 and dissected brains were fixed in 4% PFA for 16-24h. After 3 changes of 30% sucrose (Fisher) in 1X PBS each lasting 24h, brains were flash-frozen and sectioned at 18 μm. Sections were rehydrated in 1X PBS for 10 min, then permeabilized and blocked with 5% BSA and 0.3% Triton-X100 in 1X PBS for 1 hour at room temperature. Tissue sections were incubated overnight at 4°C with appropriate primary antibodies listed in "Antibodies" section diluted in 5% BSA in 1X PBS. For primary antibodies raised in mouse, a mouse on mouse (MOM) kit was used according to manufacturer's instructions (VectorLabs). Appropriate secondary antibodies diluted in 1X PBS listed in "Antibodies" section were added to tissue sections for 1h at room temperature. For nuclei visualization, tissue sections were incubated with Hoechst 33258 diluted in 1X PBS for 2 minutes at room temperature. Sections were mounted with Fluoromount G.

Immunohistochemistry of organotypic cerebellar slice cultures: Cultured cerebellar slices were quickly washed once with 1X PBS and then fixed with 4% PFA for 1h, followed by 3x washes in 1X PBS and incubation overnight in 1% Triton X-100 in 1X PBS (PBS-T) at 4°C. Next, slices were incubated in a blocking buffer containing 10% BSA, 0.1% Triton X-100 in 1X PBS for 3 h. Primary antibodies listed in "Antibodies section" were added in 6% normal donkey serum, 0.5% BSA and 1% triton X-100 in 1X PBS overnight at 4°C. After that, slices were washed three times in 1X PBS, and then blocking solution consisting of 6% normal donkey serum, 0.5% BSA and 0.1% triton X-100 was added for 1h, followed by addition of secondary antibodies and Hoechst diluted in PBS-T 1% for 3h, at room temperature. Slices were then washed 3 times for 10 min each and mounted with Fluoromount G.

Antibodies: Mouse anti-APC/CC1 (1:300, Calbiochem), mouse anti-βIII (BioLegend, 1:1000, ICC, RRID:AB_10063408), rabbit anti-βIII (BioLegend, 1:2000, RRID: AB_2564645), mouse anti-BCAS1 (Santa Cruz, 1:500, ICC and IHC, RRID: AB_10839529), sheep anti-BrdU (Abcam, 1:1000, ICC, RRID:AB_302659), rabbit anti-CC3, active form (Millipore, 1:250, ICC, RRID: AB_91556), rabbit anti-CX3CL1 (Torrey Pines Biolabs, 20μg/mL, function-blocking, RRID: AB_10891146), rabbit anti-CX3CR1 (Torrey Pines Biolabs, 40 μg/ml, function-blocking, RRID: AB_10892355), rabbit anti-DCX (Abcam, 1:400, ICC, RRID:AB_732011), rabbit anti-GFAP (Dako, 1:1000, ICC and IHC, RRID: AB_10013382), rat anti-GFAP (Thermo Fisher, 1;1000, ICC, RRID:AB_2532994), chicken anti-eGFP (Abcam, 1:1000, IHC, RRID: AB_300798), goat anti-IBA1 (Novus Bio, 1:300 for IHC, 1:1000 for ICC, RRID:AB_521594), rabbit anti-IBA1 (Wako, 1:1000, RRID: AB_839504); rabbit anti-IgG (Jackson, 20 – 40 μg/mL, function-blocking, RRID:AB_97852), mouse anti-Ki67 (BD Pharmingen, 1:300, ICC, RRID: AB_396287), rat anti-MBP (a. a. 82-87) (Millipore, 1:500, ICC and IHC, RRID: AB_94975), mouse anti-Nestin (Abcam, 1:300, ICC, RRID: AB_11211837), chicken anti-NF (light chain, BioTechne, 1:1000, IHC, RRID:AB_1556331), rabbit anti-NG2 (Chondoritin Sulfate Proteoglycan) (Millipore, 1:250, ICC, RRID: AB_91789), rabbit anti-OLIG2 (Millipore, 1:1000 for IHC, 1:2000 for ICC, immunostaining, RRID: AB_570666), mouse anti-OLIG2 (Millipore, 1:1000 for IHC, 1:500 for ICC, RRID: AB_10807410), goat anti-PDGFRα (R&D Systems, 1:400 for IHC, 1:300 for ICC, RRID: AB_2236897), fungus phalloidin-Alexa488 (ThermoFisher, 1:1000, ICC), rabbit anti-SOX2 (Cell Signalling, 1:2000, ICC, RRID: AB_2194037), goat anti-SOX2 (R&D Systems, 1:1000, IHC, RRID: AB_355110). Fluorescently labeled highly cross-absorbed secondary antibodies were purchased from Jackson ImmunoResearch and used at 1:1000 dilution. If MOM kit was used, Cy3-, DTAF-, or Cy5 conjugated streptavidin (Jackson ImmunoResearch) were used at 1:1000 dilution.

RNA Scope: RNA Scope was performed using brain cryosections from P2, P7, P15 and P65 CD1 mice as described in (2) with probes targeting murine *Cx3cr1*, *Olig22*, *Sox2* mRNA or negative control probe purchased from Advanced Cell Diagnostics according to the manufacturer's instructions. Briefly, brains were cryopreserved and sectioned at 18 μm as described in the IHC section. Sections were dehydrated with ethanol and rehydrated in 1X PBS with protease provided by RNA Scope kit and diluted 1:5 in 1xPBS for 10 min at 37°C. Sections were then washed with wash buffer provided by the RNA Scope kit and incubated with probes for 2h at 37°C. Sections were then washed and incubated with signal amplification solutions as per the manufacturer's protocol. Sections were counter-stained with DAPI (provided in the RNA scope kit) to visualize nuclei. Cells with 3 or more RNA Scope dots were considered to be positive for marker expression.

Microscopy: For RNA Scope experiments, sections were imaged with Zeiss LSM700 confocal microscope with photomultiplier tube (PMT) with 40X objective. High magnification images were taken with 5x digital zoom. Digital image acquisition was performed with Zen (Zeiss). Z-stacks of confocal images were taken with optical slice thickness 0.2-0.5 μm and stacked images are shown.

All primary cell culture experiments as well as *in vivo* experiments (except for RNA Scope) were captured with 20X or 40X objectives. Cerebellar slice images were captured by acquiring Z-stacks with optical thickness 1 μm using 10X objective. Images were captured using Zeiss Axio Imager M2 fluorescence microscope, ORCA-Flash LT sCMOS Camera and the Zen software (Zeiss). Cultured cells and *in vivo* images were imaged in single plane, except FKN-647 and BSA-647 brain sections were imaged using Z-stacks with 0.5 μm thickness and stacked images as well as orthogonal sections through 3D projections are shown. Cortical neuron-OPC co-cultures were imaged using 20X objective and Z-stacks with optical slice thickness 0.5 μm. Stacked images as well as optical (orthogonal) sections through 3D projections are shown.

QUANTIFICATION AND STATISTICAL ANALYSIS

Cell cultures were analyzed with a Zeiss Axioimager fluorescence microscope. Digital image acquisition was performed with ZEN (Zeiss) software. In all culture experiments, 5-10 fields of view were captured with a 20X objective. At least 500-2,000 cells from each treatment and biological experiment were counted. For neurosphere experiments, over 100 spheres were analyzed per condition per experiment. In OPC and NPC monolayer cultures, cells with condensed nuclei are presented as relative to total (sum of cells with condensed and healthy) nuclei. For all remaining cell culture experiments, results were presented as marker$^+$ cells relative to total healthy nuclei. Proliferation index is presented as %Ki67$^+$marker$^+$ cells over total marker$^+$ cells. OLs in co-culture were scored by their interaction with neurons. An "interacting MBP$^+$OLIG2$^+$ cell" was defined as either a "contacting MBP$^+$OLIG2$^+$ cell," in which an MBP$^+$ process was in contact with a βIII$^+$ extension (see Fig. 5L, inset III), or as a "myelinating MBP$^+$OLIG2$^+$ cell," in which an MBP$^+$ process was wrapping a βIII$^+$ extension (Fig. 5L, insets I-II). "Myelinating MBP$^+$OLIG2$^+$ cells" included MBP$^+$OLIG2$^+$ cells with thin or discontinuous βIII coverage (Fig. 5L, inset I) and MBP$^+$OLIG2$^+$ cells with heavy βIII coverage (Fig. 5L, inset II). Data was presented as either % myelinating MBP$^+$OLIG2$^+$ cells over total MBP$^+$OLIG2$^+$ cells (Fig. 5 M,O) or % interacting MBP$^+$OLIG2$^+$ cells over total MBP$^+$OLIG2$^+$ cells (Fig. 5 N,P).

In vitro and *ex vivo* data are presented from at least 3 independent biological experiments.

For cerebellar slices, myelin index was determined by manually counting the number of intersections of NF$^+$MBP$^-$ and NF$^+$MBP$^+$ fibres with 10 equidistant grids overlaid in Fiji software using same-area box (14) as described in (13, 15) (Fig. S3C,E and Fig. S4M,O). Proportion of myelinated axons indicates a sum of NF$^+$MBP$^+$ axons divided by a sum of total NF$^+$ axons in 10 grids. OLIG2$^+$ and CC1$^+$ cells were analyzed by applying same size box to cerebellar white matter. At least 2 slices per each biological replicate and 300 cells per replicate were analyzed. GFAP mean fluorescence intensity was measured in Fiji software by applying the same size box to cerebellar white matter.

For all *in vivo* experiments, dorsolateral, ventrolateral or dorsal SVZ and/or neighbouring corpus callosum was imaged using 20X objective as indicated in figures. Areas of interest were identified with Hoechst staining. For FKN-infusion experiments, the results are presented as percentage of marker$^+$YFP$^+$ cells relative to total YFP$^+$ cells. 5-8 anatomically matched sections per brain were analyzed from 6 mice across 3 independent litters. At least 250 cells per brain were counted. For FKN-647 experiments, results are presented as FKN-647$^+$marker$^+$ cells relative to total marker$^+$ cells. 3 anatomically matched sections were analyzed from 2 mice.

Images were counted in Zen or Fiji software (14). Representative images were processed in Photoshop CC 2015 and figures in Adobe Illustrator CC 2015. Biorender was used to generate cerebellar slice schematics and graphical abstract.

Sample sizes (n) indicated in the figure legends 2-7 correspond to the number of biological replicates analyzed; for RNA Scope in Fig. 1, Fig. S1 and FKN-647 infusion in Fig. 2A-G, Fig. S2A-E, data is presented from 2-4 mice per age or treatment. All data are presented as mean ± SEM.

For two group comparisons, two-tailed paired student's t-tests (*in vitro* and *ex vivo* datasets) or two-tailed unpaired student's t-tests (for *in vivo* datasets) or multiple t-tests were used to assess statistical significance between means, where a p-value <0.05 was considered significant. For three or more group comparisons one-way ANOVA was followed by Dunnett's multiple comparisons test. In all cases, Prism (version 8.0.2) was used. Number of experiments and statistical information are stated in the corresponding figure legends. In figures, asterisks denote statistical significance marked by *, $p < 0.05$; **, $p < 0.01$; ***, $p < 0.001$.

Supplemental References:

1. Mizrak D, Levitin HM, Delgado AC, Crotet V, Yuan J, Chaker Z, et al. Single-Cell Analysis of Regional Differences in Adult V-SVZ Neural Stem Cell Lineages. Cell Rep. 2019;26(2):394-406.e5.

2. Voronova A, Yuzwa SA, Wang BS, Zahr S, Syal C, Wang J, et al. Migrating Interneurons Secrete Fractalkine to Promote Oligodendrocyte Formation in the Developing Mammalian Brain. Neuron. 2017;94(3):500-16.e9.

3. Stolla M, Pelisek J, von Brühl M-L, Schäfer A, Barocke V, Heider P, et al. Fractalkine is expressed in early and advanced atherosclerotic lesions and supports monocyte recruitment via CX3CR1. PloS one. 2012;7(8):e43572-e.

4. Gu X, Xu J, Yang X-P, Peterson E, Harding P. Fractalkine neutralization improves cardiac function after myocardial infarction. Exp Physiol. 2015;100(7):805-17.

5. Cipriani R, Villa P, Chece G, Lauro C, Paladini A, Micotti E, et al. CX3CL1 is neuroprotective in permanent focal cerebral ischemia in rodents. J Neurosci. 2011;31(45):16327-35.

6. Imayoshi I, Ohtsuka T, Metzger D, Chambon P, Kageyama R. Temporal regulation of Cre recombinase activity in neural stem cells. Genesis. 2006;44(5):233-8.

7. Cardona SM, Kim SV, Church KA, Torres VO, Cleary IA, Mendiola AS, et al. Role of the Fractalkine Receptor in CNS Autoimmune Inflammation: New Approach Utilizing a Mouse Model Expressing the Human CX3CR1(I249/M280) Variant. Frontiers in cellular neuroscience. 2018;12:365-.

8. Storer MA, Gallagher D, Fatt MP, Simonetta JV, Kaplan DR, Miller FD. Interleukin-6 Regulates Adult Neural Stem Cell Numbers during Normal and Abnormal Post-natal Development. Stem Cell Reports. 2018;10(5):1464-80.

9. Coles-Takabe BL, Brain I, Purpura KA, Karpowicz P, Zandstra PW, Morshead CM, et al. Don't look: growing clonal versus nonclonal neural stem cell colonies. Stem Cells. 2008;26(11):2938-44.

10. Bhat NR, Sarlieve LL, Rao GS, Pieringer RA. Investigations on myelination in vitro. Regulation by thyroid hormone in cultures of dissociated brain cells from embryonic mice. J Biol Chem. 1979;254(19):9342-4.

11. Birgbauer E, Rao TS, Webb M. Lysolecithin induces demyelination in vitro in a cerebellar slice culture system. J Neurosci Res. 2004;78(2):157-66.

12. Stoppini L, Buchs PA, Muller D. A simple method for organotypic cultures of nervous tissue. J Neurosci Methods. 1991;37(2):173-82.

13. de Almeida MMA, Pieropan F, de Mattos Oliveira L, dos Santos Junior MC, David JM, David JP, et al. The flavonoid agathisflavone modulates the microglial neuroinflammatory response and enhances remyelination. Pharmacological Research. 2020;159:104997.

14. Schindelin J, Arganda-Carreras I, Frise E, Kaynig V, Longair M, Pietzsch T, et al. Fiji: an open-source platform for biological-image analysis. Nature Methods. 2012;9(7):676-82.

15. Al-Griw MA, Wood IC, Salter MG. Cerebellar Organotypic Slice Culture System: A Model of Developing Brain Ischaemia. Life Science Journal. 2017;14(4):89-98.
