# Supplementary material for: Fractalkine signaling regulates oligodendroglial cell genesis from SVZ precursor cells
Source: Stem Cell Reports. 2021 Jul 15;16(8):1968–84. doi: 10.1016/j.stemcr.2021.06.010 (PMC8365111; doi:10.1016/j.stemcr.2021.06.010)
Supplement: Document S2. Article plus supplemental information [file mmc2.pdf]

Stem Cell Reports
Article
OPEN ACCESS

Fractalkine signaling regulates oligodendroglial cell genesis from SVZ precursor cells

Adrianne E.S. Watson,[1,2] Monique M.A. de Almeida,[1,3] Nicole L. Dittmann,[1,3] Yutong Li,[1] Pouria Torabi,[1] Tim Footz,[1] Gisella Vetere,[4,5] Danny Galleguillos,[3,6] Simonetta Sipione,[3,6] Astrid E. Cardona,[7] and Anastassia Voronova[1,2,3,5,8,*]

[1]Department of Medical Genetics, Faculty of Medicine and Dentistry, University of Alberta, 8-39 Medical Sciences Building, Edmonton, AB T6G 2H7, Canada
[2]Women and Children's Health Research Institute, 5-083 Edmonton Clinic Health Academy, University of Alberta, 11405 87 Avenue NW Edmonton, AB T6G 1C9, Canada
[3]Neuroscience and Mental Health Institute, Faculty of Medicine and Dentistry, University of Alberta, Edmonton, AB T6G 2E1, Canada
[4]Team Cerebral Codes and Circuits Connectivity (C4), Plasticité du cerveau, ESPCI Paris, CNRS, PSL University, 75005 Paris, France
[5]Neurosciences and Mental Health Program, Hospital for Sick Children, Toronto, ON M5G 1L7, Canada
[6]Department of Pharmacology, Faculty of Medicine and Dentistry, University of Alberta, Edmonton, AB T6G 2H7, Canada
[7]Department of Biology, University of Texas at San Antonio, San Antonio, TX 78249, USA
[8]Multiple Sclerosis Centre and Department of Cell Biology, Faculty of Medicine and Dentistry, University of Alberta, Edmonton, AB T6G 2H7, Canada
*Correspondence: voronova@ualberta.ca
https://doi.org/10.1016/j.stemcr.2021.06.010

SUMMARY

Neural and oligodendrocyte precursor cells (NPCs and OPCs) in the subventricular zone (SVZ) of the brain contribute to oligodendrogenesis throughout life, in part due to direct regulation by chemokines. The role of the chemokine fractalkine is well established in microglia; however, the effect of fractalkine on SVZ precursor cells is unknown. We show that murine SVZ NPCs and OPCs express the fractalkine receptor (CX3CR1) and bind fractalkine. Exogenous fractalkine directly enhances OPC and oligodendrocyte genesis from SVZ NPCs *in vitro*. Infusion of fractalkine into the lateral ventricle of adult NPC lineage-tracing mice leads to increased newborn OPC and oligodendrocyte formation *in vivo*. We also show that OPCs secrete fractalkine and that inhibition of endogenous fractalkine signaling reduces oligodendrocyte formation *in vitro*. Finally, we show that fractalkine signaling regulates oligodendrogenesis in cerebellar slices *ex vivo*. In summary, we demonstrate a novel role for fractalkine signaling in regulating oligodendrocyte genesis from postnatal CNS precursor cells.

INTRODUCTION

Myelin, which is formed by oligodendrocytes in the central nervous system (CNS), plays an essential role in brain homeostasis by protecting nerve axons and propagating neuronal signal transduction. However, the role of oligodendrocytes and myelin extends beyond axonal support. A decrease in adult oligodendrocyte and/or new myelin genesis impairs motor skill learning, axon function, and memory formation (McKenzie et al., 2014; Steadman et al., 2019; Xiao et al., 2016). Increase in *de novo* oligodendrocyte formation and myelination can rescue motor dysfunctions (Schneider et al., 2016), spatial memory decline (Wang et al., 2020), fear memory consolidation (Pan et al., 2020), and abnormal social behaviors (Barak et al., 2019).

Resident neural precursor cells (NPCs) in the adult brain can be recruited for increased oligodendrogenesis and myelination (Murphy and Franklin, 2017). Notably, subventricular zone (SVZ) NPCs have the potential to generate oligodendrocytes, as well as astrocytes and neurons, throughout life (Maki et al., 2013; Obernier and Alvarez-Buylla, 2019). In normal homeostatic conditions, a subset of SVZ NPCs generates oligodendrocyte precursor cells (OPCs) and oligodendrocytes that populate the corpus callosum, striatum, and fimbria fornix (Menn et al., 2006). The recruitment of SVZ NPCs to form oligodendrocytes is drastically increased upon a demyelinating injury (Menn et al., 2006; Xing et al., 2014).

Intriguingly, NPCs and OPCs express chemokine receptors in the developing and adult CNS (reviewed in Watson et al., 2020). While the original defined role of chemokines is chemotaxis, they also directly participate in NPC and OPC proliferation, survival, migration, and differentiation. For example, CXCL1 and CXCL12 increase NPC/OPC proliferation and oligodendrocyte differentiation, whereas CXCL10 decreases OPC differentiation (Watson et al., 2020).

We have previously shown that developmental cortical oligodendrogenesis can be instructed by the chemokine fractalkine (CX3CL1, herein referred to as FKN) (Voronova et al., 2017). FKN secreted by cortical inhibitory neurons acts on CX3CR1 (FKN receptor) expressed in murine embryonic cortical progenitors and instructs them to differentiate into oligodendrocytes (Voronova et al., 2017). Whether FKN-CX3CR1 signaling can enhance recruitment of SVZ precursors for oligodendrocyte formation in the adult CNS remains to be addressed.

This is an open access article under the CC BY license (http://creativecommons.org/licenses/by/4.0/).

We demonstrate that postnatal and adult SVZ NPCs and OPCs express *Cx3cr1* and bind FKN. Exogenous FKN increases oligodendrogenesis from SVZ precursor cells *in vitro* and *in vivo*. We also show that OPCs secrete FKN and that inhibition of endogenous FKN signaling impairs oligodendrocyte genesis. Together, our data demonstrate FKN signaling as an important regulator of oligodendrocyte formation from precursor cells in the SVZ niche. Finally, we also show that the role of FKN signaling in oligodendrocyte genesis is preserved in the cerebellum.

RESULTS

SVZ NPCs and OPCs express *Cx3cr1* and bind FKN

In addition to microglia, which express high level of CX3CR1 (Limatola and Ransohoff, 2014), neurons and NPCs also express CX3CR1, albeit at a lower level (Ji et al., 2004; Meucci et al., 2000; Mizrak et al., 2019; Voronova et al., 2017; Wang et al., 2018). First, RNAscope analysis using probes against *Cx3cr1*, *Sox2*, and *Olig2* mRNA confirmed that *Cx3cr1* mRNA was expressed in $Sox2^{+}Olig2^{-}$ NPCs and $Sox2^{+}Olig2^{+}$ OPCs in the dorsal and dorsolateral SVZ of postnatal day (P) 2, P7, P15, and P65 mice (Figure 1) and the ventrolateral SVZ of P7, P15, P65, but not P2, mice (Figures S1A and S1B).

To find out whether FKN can bind SVZ precursors *in vivo*, we injected FKN conjugated to fluorophore Alexa Fluor 647 (FKN-647) into the lateral ventricle of adult wild-type (WT) mice, or mice with a constitutive knockout of CX3CR1 ($CX3CR1^{KO}$) (Cardona et al., 2018) (Figure 2A). As an additional control, WT mice were also injected with BSA-647. Analysis of fluorescent signal in the far-red channel demonstrated widespread diffusion of FKN-647 throughout the brain parenchyma in WT mice (Figure 2B), but not $CX3CR1^{KO}$ mice (Figure 2C), indicating that FKN-647 bound specifically to cells that express CX3CR1. Furthermore, there was no detectable signal in BSA-647-infused WT brain (Figure 2D). Immunostaining confirmed that a fluorescent signal in the far-red channel of FKN-647-infused brains was detected in $SOX2^{+}IBA1^{-}$ SVZ NPCs (Figures 2E and 2F) and was localized on the surface of SVZ NPCs (Figures S2A–S2D). Quantification revealed that ~40% of $SOX2^{+}GFAP^{+}$ and ~40% of total $SOX2^{+}$ NPCs were positive for FKN-647, with no difference between dorsal, dorsolateral, and ventrolateral regions of the SVZ (data not shown). This is in line with a previous report which showed that *Cx3cr1* is enriched in activated NPCs but not quiescent NPCs (SVZ astrocytes), ependymal cells, or neuroblasts (Mizrak et al., 2019) (Figure S2E). Fluorescent signal was also evident in $PDGFR\alpha^{+}OLIG2^{+}$ OPCs in the dorsal SVZ and adjacent corpus callosum (Figures 2E and 2G). Similar sections in BSA-647-infused WT and FKN-647-infused $CX3CR1^{KO}$ mice imaged under identical settings did not yield a positive signal (data not shown). FKN-647 also associated with $IBA1^{+}$ microglia (Figures 2F and S2F), validating this approach, as microglia are known to express high levels of CX3CR1.

To elucidate the role of FKN signaling specifically in SVZ precursor cells, we generated microglia-free NPC and OPC cultures (Figure 2H). Cells isolated from either WT or $CX3CR1^{KO}$ P7 SVZ were cultured as primary neurospheres, which were then dissociated and incubated as adherent cells either for 1 day *in vitro* (DIV) in NPC medium containing fibroblast growth factor (FGF) and epidermal growth factor (EGF), or for 2 DIV in OPC growth medium (GM) containing FGF and platelet-derived growth factor AA (PDGF-AA) (Figure 2H). WT NPC cultures were nearly 100% positive for NPC markers SOX2 and NESTIN (Figures 2I and 2Ii), but not OPC markers NG2 or PDGFRα (Figure 2Ii). WT OPC cultures were composed predominantly of OPC cells, as they were 100% $SOX2^{+}$, 94.32% ± 0.94% $OLIG2^{+}$, 62.03% ± 5.91% $NG2^{+}$, and 61.12% ± 5.14% $PDGFR\alpha^{+}$ (Figures 2J and 2Ji). Importantly, none of the cells were positive for the microglial marker IBA1 (Figures S2G–S2I). $CX3CR1^{KO}$ NPC cultures displayed high levels of $SOX2^{+}$ and $NESTIN^{+}$ cells (92.5% ± 1.5% and 91.5% ± 1.9%, respectively), whereas $CX3CR1^{KO}$ OPC cultures had reduced proportions of $OLIG2^{+}$ and $PDGFR\alpha^{+}$ cells (64.2% ± 12.7% and 13.9% ± 4.6%, respectively) (Figures 2Ki and 2Mi). This supports and expands our previous work, in which $CX3CR1^{KO}$ mice showed reduced OPCs in the neonatal cortex (Voronova et al., 2017).

In these cultures, both WT NPCs and OPCs exhibited robust binding of FKN-647 (Figures 2I and 2J) but not BSA-647 (Figures 2L and 2N). 87.40% ± 2.66% NPCs bound FKN-647, which could be further divided into two groups: $FKN\text{-}647^{BRIGHT}$ (56.03% ± 4.80%) and $FKN\text{-}647^{DIM}$ (31.37% ± 4.52%) cells (Figures 2I and 2Ii). OPC cultures showed 48.13% ± 7.84% $FKN\text{-}647^{BRIGHT}$ and 34.89% ± 5.42% $FKN\text{-}647^{DIM}$ OPCs yielding overall 83.02% ± 8.53% $FKN\text{-}647^{+}$ OPCs (Figures 2J and 2Ji). The distinct levels of FKN-647 fluorescence may represent varying levels of FKN receptor expression in these precursor cells, reflecting the heterogeneity of these cell groups (Marques et al., 2018; Mizrak et al., 2019). Importantly, $CX3CR1^{KO}$ NPCs and OPCs displayed greatly reduced FKN-647 binding (9.9% ± 3.0% and 21.8% ± 4.7%, respectively) (Figures 2K, 2Ki, 2M, and 2Mi). Residual FKN-647 binding in cultured cells may reflect expression of other receptors that can bind FKN, such as integrins (Fujita et al., 2012), non-specific binding, or binding via a yet unidentified receptor.

Together, these results demonstrate that postnatal SVZ NPCs and OPCs express *Cx3cr1* and bind FKN. These data support and extend previous reports showing that postnatal NPCs and OPCs express *Cx3cr1* as determined via

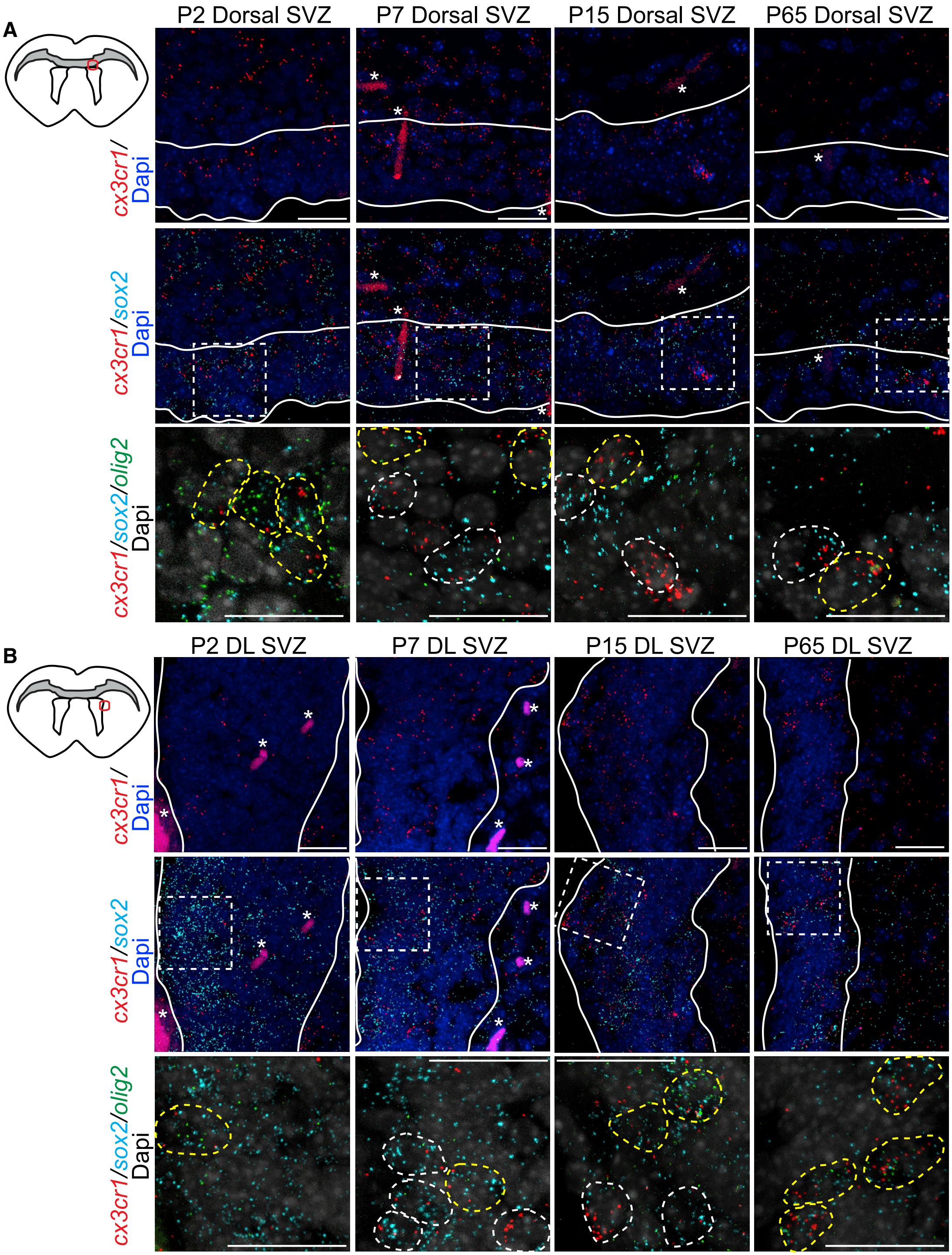

Figure 1. SVZ NPCs and OPCs express *Cx3cr1 in vivo*

(A and B) Top left: approximate location of dorsal (A) and dorsolateral (DL) (B) SVZ (red circle) images shown here. Top row: RNAscope analysis of dorsal (A) and DL (B) SVZ from P2, P7, P15, and P65 SVZ for *Cx3cr1* (red) mRNA. Middle row: double-label RNAscope analysis for *Cx3cr1* (red) and *Sox2* (cyan) mRNAs. Hatched boxes indicate section shown at higher magnification in bottom row. Bottom row: triple-label RNAscope analysis for *Cx3cr1* (red), *Sox2* (cyan), and *Olig2* (green). White dashed circles indicate *Cx3cr1*$^{+}$*Sox2*$^{+}$ and yellow dashed circles *Cx3cr1*$^{+}$*Sox2*$^{+}$*Olig2*$^{+}$ cells. Asterisks indicate blood vessels. n = 2–3 for each age. Scale bars, 20 μm. See also Figure S1.

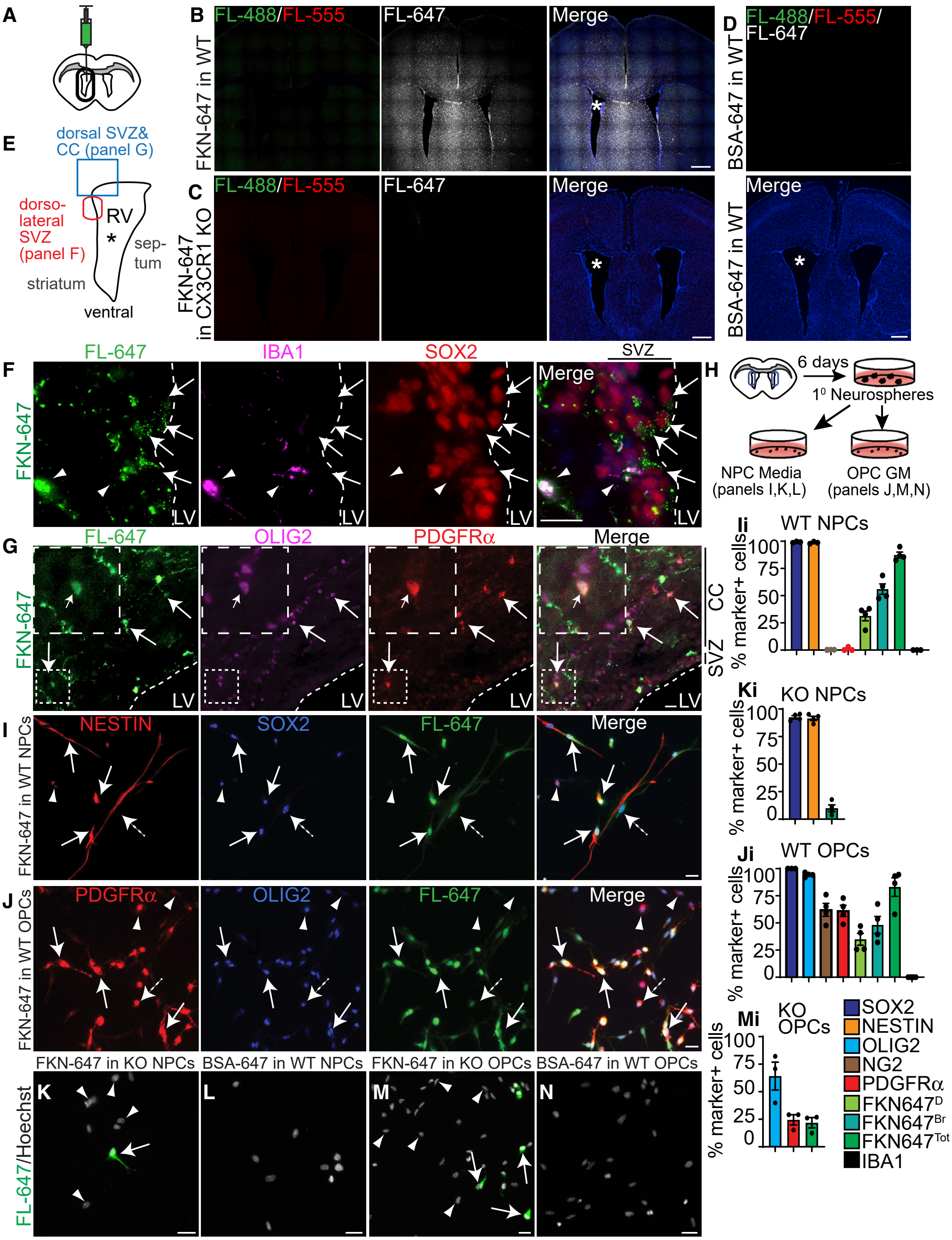

Figure 2. SVZ NPCs and OPCs bind FKN *in vivo* and *in vitro*

(A) WT or CX3CR1^{KO} mice were injected once with FKN-647 and/or BSA-647 into the right lateral ventricle (RV, asterisk).

(B–D) Coronal section of WT brain infused with FKN-647 (B) or BSA-647 (D), or CX3CR1^{KO} brain infused with FKN-647 (C). Fluorescence in all channels was captured using identical settings.

(E) Approximate location of images in (F) and (G).

(legend continued on next page)

RNA sequencing (Marques et al., 2018; Mizrak et al., 2019; reviewed in Watson et al., 2020).

FKN increases oligodendrocyte genesis from SVZ NPCs in culture

To test whether FKN can mediate oligodendrocyte genesis from NPCs, we cultured WT SVZ NPC monolayers for 3–5 days with varying concentrations of FKN (Figure 3A). Immunostaining at 3 DIV revealed an FKN-mediated significant ~38% increase in PDGFRα$^+$ OPCs (Figures 3B and 3C) in a concentration-dependent manner when compared with vehicle control (VC, PBS). By 4–5 days, there was a trending increase in PDGFRα$^+$ cells in the presence of FKN, though not significant (Figures 3B and 3D). However, there was a statistically significant ~40% increase in MBP$^+$ oligodendrocytes (Figures 3E and 3I) on 5DIV in a concentration-dependent manner in the presence of FKN. The greatest effect was seen with the intermediate FKN concentration (250 ng/mL).

As NPCs can produce astrocytes and neurons in addition to oligodendrocytes, we examined whether FKN may bias precursor cells to produce oligodendroglia at the expense of these cell types. Immunostaining revealed no change in GFAP$^+$ astrocytes (Figures 3F and 3J), DCX$^+$ neuroblasts (Figures 3H and 3L), or βIII$^+$ neurons (Figures 3G and 3K) in cultures incubated with the most effective concentration of FKN (250 ng/mL). Since FKN has been shown to reduce neuronal cell death in stroke and synucleinopathy mouse models (Cipriani et al., 2011; Nash et al., 2015), we then considered that FKN may have an effect on cell death. However, FKN did not affect the number of cells with condensed nuclei at either time point (Figures 3M and 3N). Together, this indicates that FKN enhances the production of OPCs and oligodendrocytes from SVZ NPCs *in vitro*.

FKN does not affect NPC or OPC proliferation

We then asked whether FKN achieves the observed increase in the proportion of oligodendroglial cells by modulating NPC and/or OPC proliferation. First, exogenous FKN did not affect the number of secondary neurospheres formed when compared with VC (PBS, Figures 4A and 4B). Adherent 1-DIV NPCs similarly did not show differences in the proportion of Ki67$^+$ (Figure 4E), bromodeoxyuridine (BrdU$^+$) (Figure 4F), or SOX2$^+$ cells (Figure 4D) in FKN versus VC.

To determine whether FKN could mediate OPC proliferation, we analyzed NPC monolayer cultures at 3 DIV, when we detected 10%–20% PDGFRα$^+$ OPCs (Figure 3C). FKN had no effect on the proportion of Ki67$^+$ cells (Figures 4G and 4H) or the proliferative index of PDGFRα$^+$ or OLIG2$^+$ cells when compared with VC (Figures 4I and 4J). To corroborate these results, we generated SVZ OPCs, which were then incubated with VC or 250 ng/mL FKN in OPC GM for 20–24 h (Figure 5A). There was no change in the proportion of Ki67$^+$ cells or the proliferative index of PDGFRα$^+$ cells (Figures 5B–5D). In addition, there was no effect on cells with condensed nuclei or CC3$^+$ cells (Figures 5E and 5F).

This suggests that the pro-oligodendrogenic effect of FKN is due to enhanced NPC commitment and/or OPC differentiation, rather than modulation of proliferation or cell death.

FKN accelerates oligodendrocyte differentiation from OPCs *in vitro*

To corroborate the effect of FKN on OPC differentiation using an independent platform, we subjected SVZ OPCs to medium supplemented with thyroid hormone T3 (differentiation medium [DM]) and FKN or VC. OPCs incubated with FKN showed a statistically significant 31.52% ± 11.77% increase in MBP$^+$ cells at 2 DIV but not 5 DIV (Figures 5G–5I). The proportion of cells with condensed nuclei was unaffected at either time point (Figure 5J). Thus, exogenous FKN is sufficient to accelerate OPC differentiation into oligodendrocytes.

To assess whether FKN can increase the number of myelinating oligodendrocytes, we co-cultured SVZ OPCs with

(F) Dorsolateral SVZ of FKN-647 infused WT brain immunostained for IBA1 (purple) and SOX2 (red). Arrows indicate FKN-647$^+$SOX2$^+$ and arrowheads FKN-647$^+$SOX2$^-$IBA1$^+$ cells.
(G) Dorsal SVZ and corpus callosum (CC) of FKN-647 infused WT brain immunostained for OLIG2 (purple) and PDGFRα (red). Arrows indicate FKN-647$^+$OLIG2$^+$PDGFRα$^+$ cells. Fluorescence (FL) in far-red channel (FL-647) in (F) and (G) is pseudo-colored in green. Dashed lines indicate SVZ boundary. LV, lateral ventricle.
(H) Schematic of microglia-free SVZ NPC and OPC cultures.
(I) 1-DIV WT NPCs incubated with FKN-647 (green) and immunostained for NESTIN (red) and SOX2 (blue). Arrows indicate NESTIN$^+$SOX2$^+$FKN-647$^{Bright\ (Br)+}$, dashed arrow NESTIN$^+$SOX2$^+$FKN-647$^{Dim\ (D)+}$, and arrowhead NESTIN$^+$SOX2$^+$FKN-647$^-$ cells.
(J) 2-DIV WT OPCs incubated with FKN-647 (green) and immunostained for PDGFRα (red) and OLIG2 (blue). Arrows indicate PDGFRα$^+$OLIG2$^+$FKN-647^{Br+}, dashed arrow PDGFRα$^+$OLIG2$^+$FKN-647^{D+}, and arrowhead PDGFRα$^+$OLIG2$^+$FKN-647$^-$ cells.
(K and M) 1-DIV NPCs (K) or 2-DIV OPCs (M) from CX3CR1^{KO} SVZ incubated with FKN-647 (green). Arrows indicate FKN-647$^+$ cells, arrowheads FKN-647$^-$ cells.
(L and N) 1-DIV NPCs (L) or 2-DIV OPCs (N) from WT SVZ incubated with BSA-647 (green). Fluorescence in far-red channel (FL-647) was captured using identical settings in (I) to (N).
(Ii–Mi) Quantification of (I), (K), (J), and (M). Error bars represent SEM, n = 3–4 experiments. Scale bars, 20 μm (A, F, G, I–N) and 500 μm (B–D). See also Figure S2.

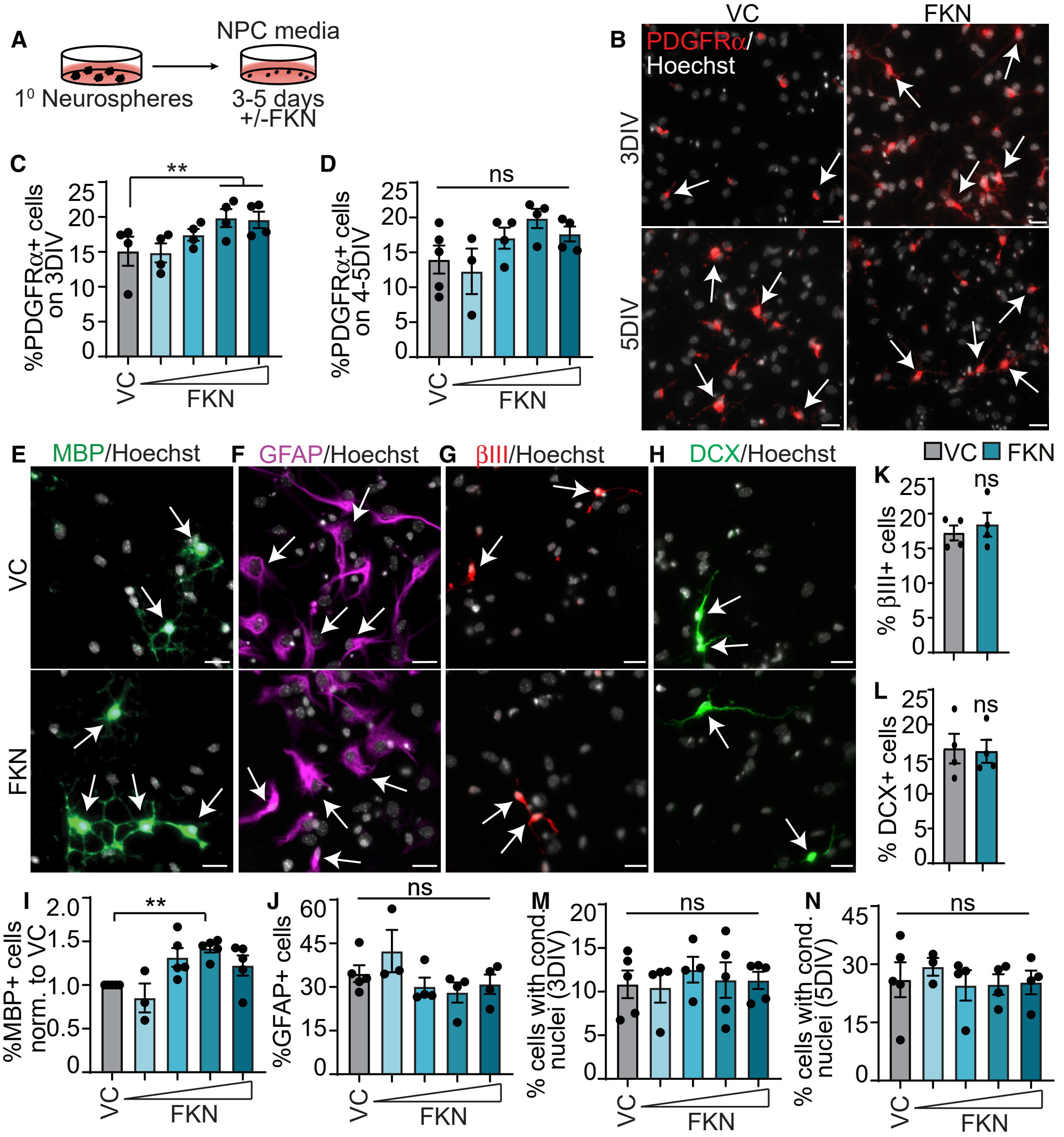

Figure 3. FKN increases oligodendrogenesis from SVZ NPCs *in vitro*

(A) Schematic: P7 SVZ primary neurosphere cells were cultured as adherent NPCs for 3–5 DIV with 25–500 ng/mL FKN or VC. Representative images show cells incubated with 250 ng/mL FKN.

(B) 3-DIV or 5-DIV NPCs cultured with VC (left) or FKN (right) and immunostained for PDGFRα (red).

(C and D) Quantification of (B).

(E–H) 4- to 5-DIV NPCs cultured with VC (top) or FKN (bottom) and immunostained for MBP (green, E), GFAP (purple, F), βIII (red, G), and DCX (green, H).

(I–N) Quantification of (E) to (H). Results were normalized to VC in (I). In all images arrows indicate marker-positive cells.

Cells were counterstained with Hoechst (gray). Graphs were analyzed with one-way ANOVA followed by Dunnett's multiple comparisons test, except (K) and (L), analyzed with paired t test. Error bars represent SEM, n = 3–5 experiments. ns, not significant. Scale bars, 20 μm.

cortical neurons in DM with FKN or VC (Figure 5K). MBP^+O-$LIG2^+$ oligodendrocytes were scored based on their interaction with βIII^+ neurons (Figure 5L). Interacting oligodendrocytes were defined as a sum of "contacting" (Figure 5L, inset III) and "myelinating" cells (Figure 5L, insets I–II). By 2–3 DIV, there was no difference in either myelinating or interacting oligodendrocytes (Figures 5M and 5N). However, after 5–6 DIV, FKN-treated cultures showed a trending increase in myelinating oligodendrocytes that did not reach statistical significance and a statistically significant

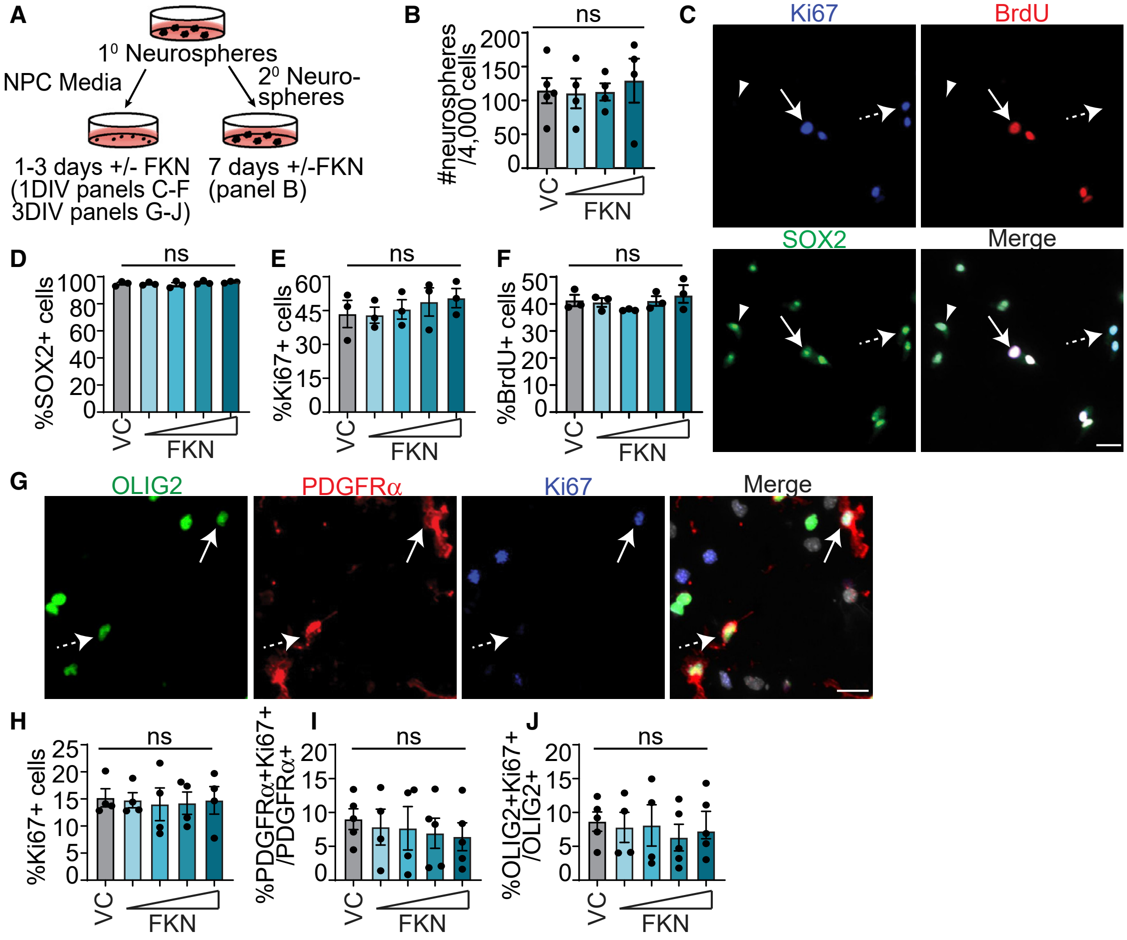

Figure 4. FKN does not affect NPC or OPC proliferation

(A) Schematic: P7 SVZ primary neurosphere cells were cultured as adherent NPCs for 1 DIV (C–F) or 3 DIV (G–J), or as secondary neurospheres for 7 DIV (B) with 25–500 ng/mL FKN or VC.

(B) Number of secondary neurospheres generated from 4,000 primary neurosphere cells.

(C) 1-DIV NPCs in VC and immunostained for Ki67 (blue), BrdU (red), and SOX2 (green). Arrow indicates a $Ki67^+SOX2^+BrdU^+$ cell, dashed arrow $Ki67^+SOX2^+BrdU^-$ cell, and arrowhead $SOX2^+Ki67^-BrdU^-$ cell.

(D–F) Quantification of (C).

(G) 3-DIV NPCs in VC and immunostained for OLIG2 (green), PDGFRα (red), and SOX2 (blue). Arrow indicates an $OLIG2^+PDGFR\alpha^+Ki67^+$ cell and dashed arrow $OLIG2^+PDGFR\alpha^+Ki67^-$ cell.

(H–J) Quantification of (G).

In all images cells were counterstained with Hoechst (gray). Error bars represent SEM, n = 4–5 experiments. All graphs were analyzed with one-way ANOVA followed by Dunnett's multiple comparisons test. ns, not significant. Scale bars, 20 μm.

16.65% ± 4.40% increase in the proportion of oligodendrocytes interacting with axons (Figures 5O and 5P). Therefore, FKN increases the proportion of oligodendrocytes that interact with cortical neurons, which may have implications for myelination.

FKN enhances oligodendroglial lineage cell formation from SVZ NPCs *in vivo*

To find out whether FKN promotes oligodendrogenesis *in vivo*, we infused FKN into the lateral ventricle of 3-month-old $NestinCre^{ERT2}$;$RosaYFP^{STOP/+}$ NPC lineage-tracing mice. NPCs and their progeny were labeled with YFP expression via tamoxifen injections, followed by intracerebral-ventricular (ICV) infusion of VC (BSA) or FKN (Figure 6A). We analyzed the corpus callosum area above the ventricle, which was further divided into the SVZ and white matter (WM) regions (Xing et al., 2014). FKN treatment caused a slight, but not significant, shift of YFP^+ cells from the dorsal SVZ to the WM (Figure 6C).

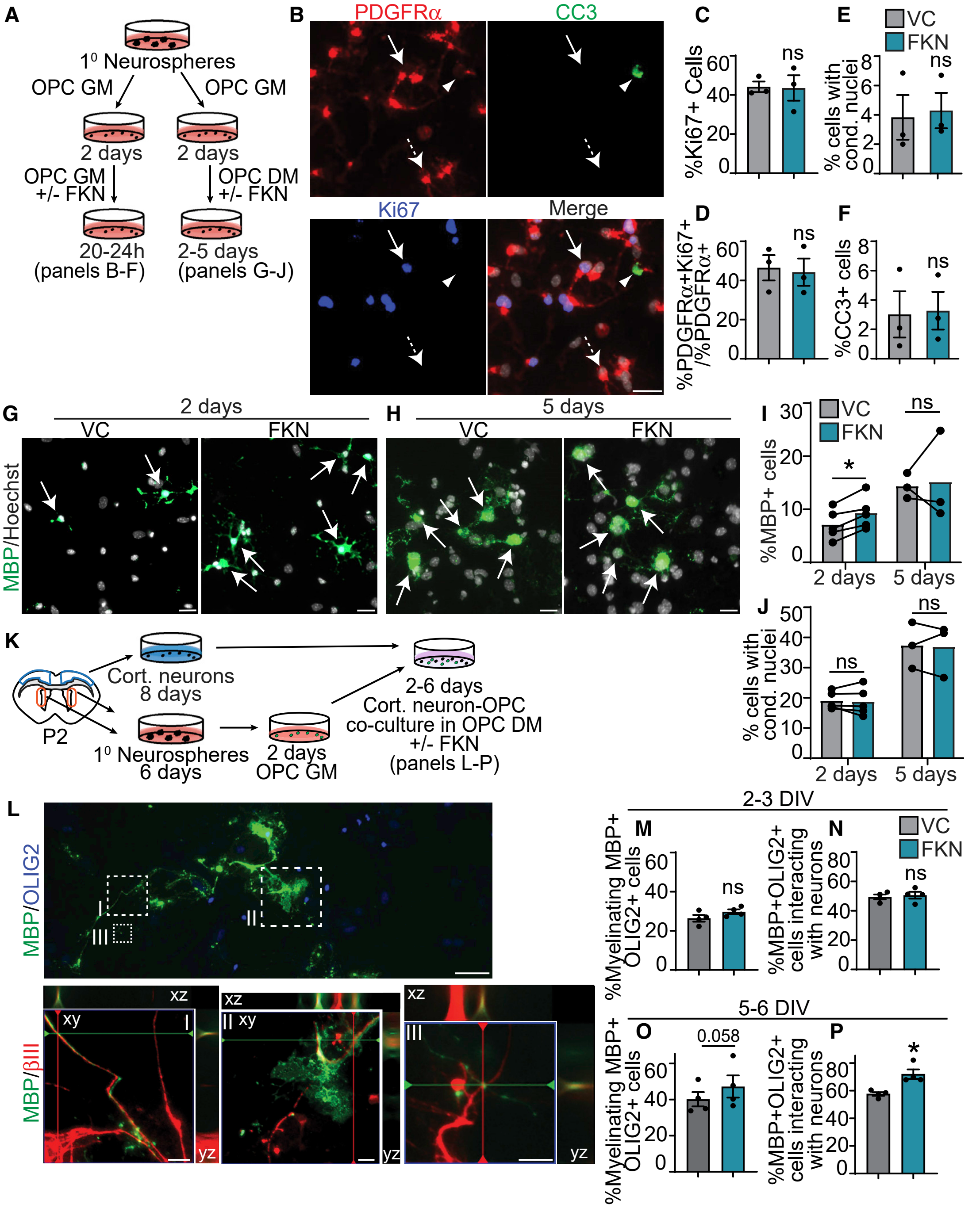

Figure 5. FKN accelerates SVZ OPC differentiation

(A) Schematic: P7 SVZ primary neurosphere cells were cultured in OPC GM for 2 DIV, followed by either treatment with 250 ng/mL FKN or VC in OPC GM for 20–24 h (left, B–F) or in OPC differentiation medium (DM) for 2–5 days (right, G–J).

(B) Representative images from OPCs in GM supplemented with VC, immunostained for PDGFRα (red), cleaved caspase-3 (CC3, green), and Ki67 (blue). Arrow indicates PDGFRα^{+}Ki67^{+}CC3^{-} cell, dashed arrow PDGFRα^{+}Ki67^{-}CC3^{-} cell, and arrowhead PDGFRα^{+}Ki67^{-}CC3^{+} cell.

(C–F) Quantification of (B).

(G and H) OPCs cultured in DM with VC (left) or FKN (right) for 2 DIV (G) or 5 DIV (H), immunostained for MBP (green) and counterstained with Hoechst (gray). Arrows indicate MBP^{+} cells.

(I and J) Quantification of (G) and (H).

(legend continued on next page)

To assess whether infusion of FKN caused a shift in SVZ NPC differentiation, we quantified the proportion of YFP$^+$ cells that co-expressed OPC marker PDGFRα, early oligodendrocyte marker BCAS1 (Fard et al., 2017; Ishimoto et al., 2017), or mature oligodendrocyte marker CC1 (Bhat et al., 1996). FKN infusion led to a ~2-fold increase in the proportion of YFP$^+$PDGFRα$^+$ OPCs in the SVZ and a ~1.4-fold increase in the WM (Figures 6D–6F) when compared with VC. FKN had no effect on the proportion of newly formed YFP$^+$BCAS1$^+$ oligodendrocytes in the SVZ, but caused a significant ~1.4-fold increase in *de novo* early oligodendrocytes in the WM (Figures 6G–6I). Similarly, FKN-infused animals showed a slight but non-significant increase in YFP$^+$CC1$^+$OLIG2$^+$ cells in the SVZ and a statistically significant ~1.4-fold increase in YFP$^+$CC1$^+$OLIG2$^+$ oligodendrocytes in the WM (Figures 6J–6L). These data validate our *in vitro* findings and provide further evidence that FKN enhances the production of oligodendroglial lineage cells from SVZ NPCs.

FKN increases *ex vivo* cerebellar OPC differentiation

To determine whether FKN can modulate oligodendrocyte formation in other brain areas, we incubated cerebellar slices with FKN or VC (PBS) (Figure 6M). While the postnatal cerebellum does not host NPCs, cerebellar slice cultures provide an *ex vivo* assay to assess developmental OPC differentiation and myelination (Shen and Yuen, 2020). To assess OPC differentiation, we analyzed deep cerebellar WM (Figure S3B, yellow box) immunostained with anti-OLIG2 and anti-CC1 (Figure 6N). FKN did not lead to changes in the total number of OLIG2$^+$ cells (Figure 6O). However, there was a 28.55% ± 9.74% increase in CC1$^+$OLIG2$^+$ oligodendrocytes and a 26.62% ± 8.66% decrease in CC1$^-$OLIG2$^+$ OPCs and immature oligodendrocytes (Figures 6P and 6Q). To assess changes in OPC fates, we normalized data to the total number of OLIG2$^+$ cells. This analysis showed that FKN increased the proportion of CC1$^+$OLIG2$^+$ cells, and decreased the proportion of CC1$^-$OLIG2$^+$ cells (Figure 6R). FKN treatment did not affect the relative proportion of GFAP signal (Figures S3B, S3D, and S3F), in line with our SVZ precursor *in vitro* data.

Next, we assessed the proportion of myelinated axons by analyzing the intersections of MBP$^+$NF$^+$ (neurofilament) to total NF$^+$ axons with ten equidistant grids, as previously described (de Almeida et al., 2021) (Figure S3). In FKN-treated slices, there was a significant ~20% increase in the proportion of myelinated axons compared with VC (Figures S3B, S3C, and S3E).

Inhibition of FKN signaling reduces oligodendrocyte differentiation

RNA-seq data from purified OPCs (Marques et al., 2018) show that P7 brain and spinal cord OPCs robustly express *Fkn* mRNA when compared with embryonic day 13.5 (E13.5) OPCs (Figure 7A). Enzyme-linked immunosorbent assay (ELISA) revealed that SVZ OPC-conditioned medium contained 994 ± 110 pg/mL FKN (Figure 7B).

To discover whether endogenous FKN regulates OPC fates, we cultured OPCs with a function-blocking antibodies specific to FKN (anti-FKN) or CX3CR1 (anti-CX3CR1), which are known to block FKN signaling *in vivo* and *in vitro* (Cipriani et al., 2011; Voronova et al., 2017). SVZ OPCs cultured in DM with anti-FKN or anti-CX3CR1 (Figure 7C) showed a statistically significant ~25%–30% decrease in MBP$^+$ oligodendrocytes (Figures 7D and 7E) and a concomitant 35%–40% increase in PDGFRα$^+$ OPCs (Figures 7F and 7G) compared with isotype-matched immunoglobulin G (IgG). OPCs are known to differentiate into astrocytes (Suzuki et al., 2017). Our data indicated that anti-FKN and anti-CX3CR1 caused a 20%–37% increase in GFAP$^+$ astrocytes when compared with IgG (Figures 7H and 7I).

We then asked whether the differences in OPC differentiation were due to changes in OPC proliferation. Analysis of OPCs in GM containing anti-FKN or anti-CX3CR1 (Figure S4A) did not support changes in the proportion of PDGFRα$^+$ or Ki67$^+$ cells or in the proliferative index of PDGFRα$^+$ cells (Figures S4B–S4G). The observed effect on MBP$^+$ and GFAP$^+$ cell formation also appeared to not be due to changes in cell death, as the proportion of cells with condensed nuclei or CC3$^+$ cells was unchanged (Figures 7J–7K and S4H–S4K).

To confirm the effect of FKN signaling inhibition on oligodendrocyte differentiation, we cultured P7 SVZ NPCs in NPC medium supplemented with anti-FKN or IgG for 4–5 days (Figure 7L). Anti-FKN treatment showed a statistically significant 48.57% ± 8.43% decrease in BCAS1$^+$ oligodendrocytes (Figures 7M and 7O), 19.56% ± 4.38% increase

(K) Schematic: cortical neurons and SVZ OPCs derived from P1-2 CD1 pups were co-cultured for 2–6 days in OPC DM with FKN/VC.
(L) Cortical neuron-OPC co-culture, immunostained for MBP (green), OLIG2 (blue), and βIII (red). Top: overlay of MBP and OLIG2. Hatched boxes are shown at higher magnification in bottom row. Bottom: magnified image of hatched boxes represent an orthogonal slice through a z-stack.
(M–P) Quantification of myelinating (M and O) or interacting (N and P) MBP$^+$OLIG2$^+$ expressed as percentage of total number of MBP$^+$OLIG2$^+$ cells after 2–3 DIV (M and N) and 5–6 DIV (O and P) of co-culture.
Error bars represent SEM, n = 3–5 experiments. All graphs were analyzed with paired t test. ns, not significant. Scale bars, 20 μm (B and G), 50 μm (L) and 10 μm (L, insets).

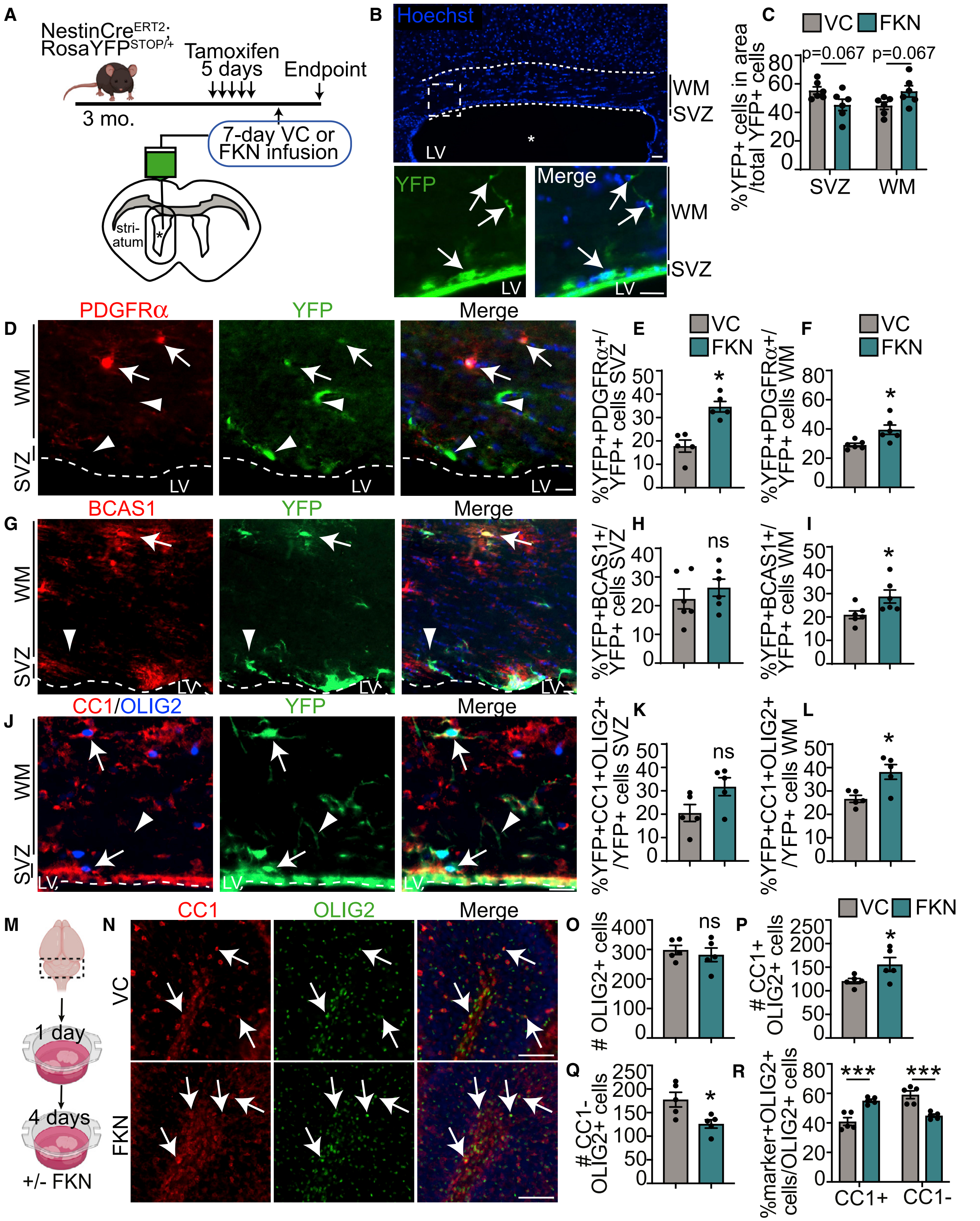

(legend on next page)

in GFAP$^+$ astrocytes (Figures 7M and 7P), 37.33% ± 5.13% decrease in βIII$^+$ neurons (Figures 7M and 7Q), and no change in PDGFRα$^+$ OPCs (Figure 7N) or cells with condensed nuclei (Figure 7R) when compared with IgG. Taken together, these results suggest that (1) OPCs are a source of FKN, (2) FKN signaling is an important regulator of SVZ precursor cell fate, and (3) inhibition of FKN signaling reduces oligodendrocyte and neuronal differentiation and possibly increases astrocyte formation.

FKN signaling inhibition reduces *ex vivo* cerebellar oligodendrocyte genesis

Given that anti-FKN resulted in decreased oligodendrocyte formation from SVZ precursors *in vitro*, we then asked whether inhibition of FKN signaling could also impede oligodendrocyte genesis from precursors in other brain areas, such as the cerebellum. We treated cerebellar slices with anti-FKN or IgG for 4 days (Figures 7S and S4L). Anti-FKN treatment led to a statistically significant 58.53% ± 4.32% decrease in total OLIG2$^+$ cells, a 64.04% ± 3.22% decrease in CC1$^+$OLIG2$^+$ oligodendrocytes, and a 49.99% ± 7.02% decrease in CC1$^-$OLIG2$^+$ cells (Figures 7T–7X). To find out whether anti-FKN changed the fate of OPCs, we presented the data as a proportion of total OLIG2$^+$ cells. Similarly to our *in vitro* data, anti-FKN treatment resulted in a modest, but statistically significant 12.87% ± 3.76% decrease in the proportion of CC1$^+$OLIG2$^+$ cells and a 20.04% ± 6.81% increase in CC1$^-$OLIG2$^+$ cells (Figure 7Y). Additionally, there was a slight but non-significant increase in GFAP signal (Figures S4N and S4P). Finally, anti-FKN led to a ~1.6-fold decrease in the proportion of myelinated MBP$^+$NF$^+$ axons (Figures S4M and S4O). Together, these data indicate that FKN signaling is important for appropriate cerebellar oligodendrocyte formation and potentially, myelination.

DISCUSSION

Our data demonstrate a novel role for FKN signaling in postnatal oligodendrogenesis from SVZ precursor cells, and suggest that FKN is a candidate molecule for engagement of CNS precursor cells for oligodendrocyte production and, possibly, remyelination.

The adult mammalian brain contains two stem cell niches, the SVZ and subgranular zone (SGZ). NPCs in both niches produce neurons and astrocytes throughout life. However, typically only SVZ NPCs are capable of producing oligodendrocytes (Obernier and Alvarez-Buylla, 2019). Intriguingly, infusion of exogenous FKN into the hippocampus increases SGZ neurogenesis in aged rats (Bachstetter et al., 2011) and rescues neurogenesis and hippocampal-dependent memory deficits in the BDNF$^{Met/Met}$ mouse model of schizophrenia (Wang et al., 2014), implicating FKN's role in the activation and/or differentiation of NPCs. Within the SVZ, overexpression of soluble FKN in cells lining the lateral ventricle rescues cognitive function in an rTg450 tauopathy mouse model (Finneran et al., 2019). Our results extend these studies and show that infusion of FKN into the lateral ventricle increases SVZ oligodendrogenesis.

Previous reports suggest FKN can activate and enhance the survival of SVZ NPCs. Adult rodent SVZ NPCs respond to FKN *in vitro* via increased production of primary neurospheres (Silva-Vargas et al., 2016). Our results show that FKN does not alter the formation of secondary neurospheres from SVZ NPCs isolated from young rodents. This discrepancy may be attributed to analysis of secondary versus primary neurospheres or the age of the animals. Furthermore, human fetal NPCs express CX3CR1 and show increased cell survival in response to FKN when growth factors are depleted (Krathwohl and Kaiser, 2004).

Figure 6. FKN enhances oligodendrogenesis from SVZ NPCs *in vivo* and from cerebellar OPCs *ex vivo*
(A) Schematic: NestinCre^{ERT2};RosaYFP$^{STOP/+}$ were injected with tamoxifen and infused with FKN or VC into the lateral ventricle (asterisk) for 7 days via osmotic mini-pump. Images from corpus callosum lining dorsally to the infused ventricles were analyzed in (B) to (L).
(B) Representative image of VC-infused ventricle. Dashed borders indicate an area analyzed in (C) to (L) and encompass SVZ and white matter (WM). Bottom: hatched box from top shown at higher magnification. Arrows indicate YFP$^+$ cells.
(C) Analysis of YFP$^+$ cell distribution in (B).
(D, G, and J) Dorsal SVZ and WM in FKN-infused ventricle and immunostained for YFP (green) and PDGFRα (red, D), BCAS1 (red, G), or CC1 (red, J) and OLIG2 (blue, J). Arrows indicate marker-positive YFP$^+$ cells, and arrowheads marker-negative YFP$^+$ cells. Dashed line indicates SVZ boundary. LV, lateral ventricle.
(E and F) Quantification of (D).
(H and I). Quantification of (G).
(K and L) Quantification of (J). n = 5–6 mice from three litters.
(M) Schematic: cerebellar slices from P10–P11 CD1 mice were cultured with FKN or VC.
(N) Cerebellar slices incubated with VC (top) or FKN (bottom) and immunostained with CC1 (red) and OLIG2 (green). Arrows indicate CC1$^+$OLIG2$^+$ cells.
(O–R) Quantification of (N). n = 5–6 biological replicates.
Data were analyzed using unpaired t test in (D) to (L), paired t test in (O) to (Q), and multiple t test in (C) and (R). Error bars represent SEM. ns, not significant. In all images tissue was counterstained with Hoechst (blue) to visualize nuclei. Scale bars, 50 μm (B), 20 μm (D, G, and J), and 100 μm (N). See also Figure S3.

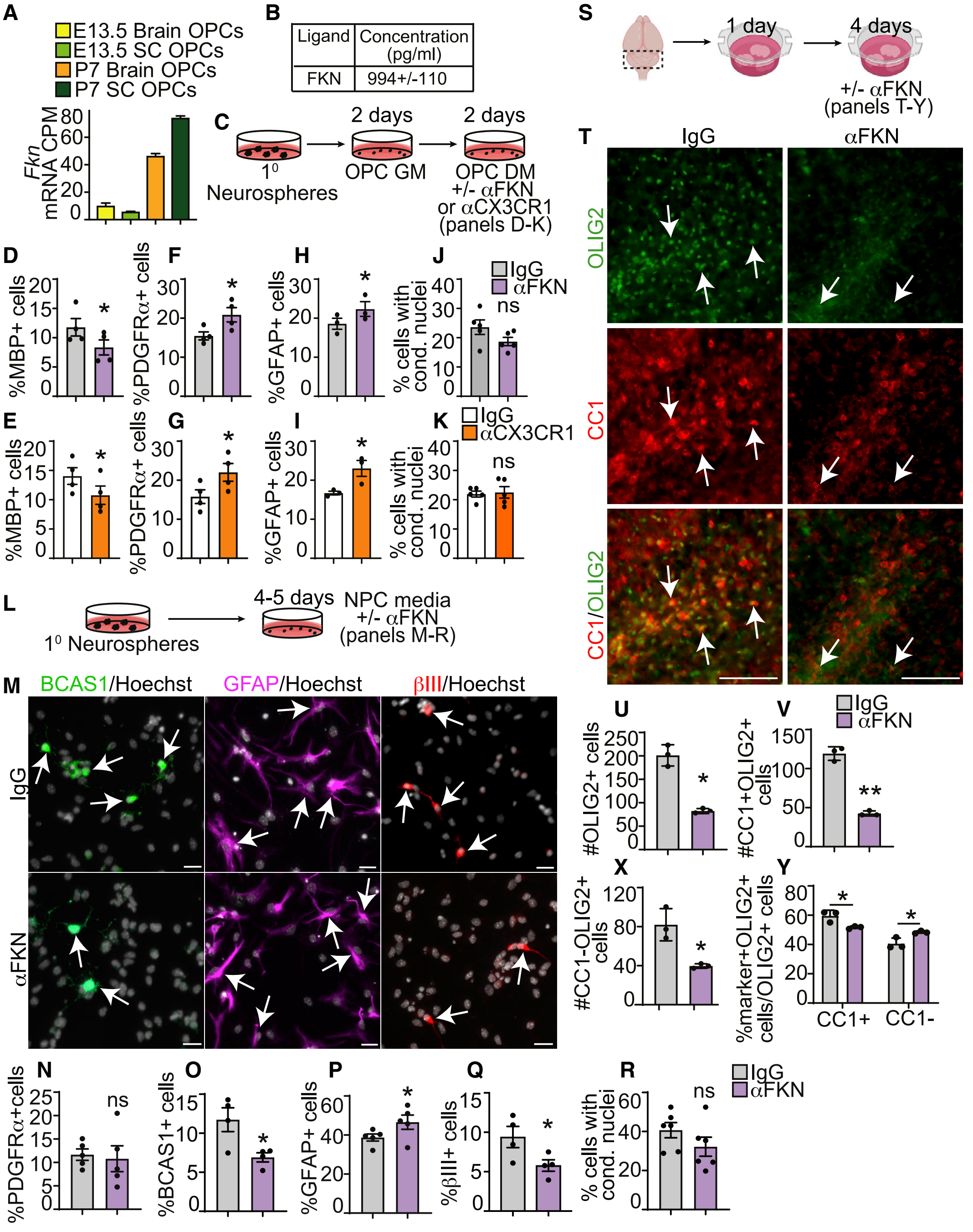

Figure 7. Inhibition of FKN signaling reduces oligodendrogenesis *in vitro* and *ex vivo*

(A) *Fkn* mRNA expression in purified E13.5 and P7 murine OPCs extracted from RNA-seq analysis using Ki.se/en/mbb/oligointernode (Marques et al., 2018, GEO: GSE95093).

(B) ELISA analysis of FKN in medium conditioned for 2–3 days by P7 SVZ OPCs, in pg/mL. n = 3 independent preparations.

(C) Schematic: P7 SVZ primary neurosphere cells were cultured as OPCs in OPC GM for 2 DIV, followed by 2 DIV in OPC DM with function-blocking antibodies αFKN, αCX3CR1, or non-specific IgG.

(D–K) Quantification of MBP^+ (D and E), $PDGFR\alpha^+$ (F and G), or $GFAP^+$ (H and I) cells, or cells with condensed nuclei (J and K) and cultured with αFKN (D, F, H, and J) or αCX3CR1 (E, G, I, and K).

(L) Schematic: P7 SVZ primary neurosphere cells were cultured as adherent NPCs for 4–5 DIV with αFKN or IgG.

(legend continued on next page)

Our results support and extend these studies by demonstrating that SVZ NPCs and OPCs express CX3CR1 as well as bind and respond to FKN by increasing oligodendroglial cell formation without changes in proliferation or survival.

Our FKN-647 data suggest that CX3CR1 protein may be differentially expressed and localized to the surface of various CNS cells. For example, FKN-647 outlines the soma and processes in IBA1$^+$ microglia but appears as puncta in SOX2$^+$ NPCs. The pattern of FKN-647 binding to adult SVZ NPCs *in vivo* resembles EGF receptor immunostaining (Belenguer et al., 2021). Our *in vitro* data, however, show that FKN-647 outlines P7 SVZ NPC and OPC cell soma and processes. It is possible that cultured cells versus cells within tissue architecture, or young versus adult SVZ precursor cells, display different levels and patterns of CX3CR1 expression. Moreover, Mizrak et al. (2019) showed that activated (proliferating) SVZ NPCs have enriched *Cx3cr1* mRNA expression. Cultured NPCs are activated and highly proliferating, so they may display increased CX3CR1 expression. Together, our FKN-647 results align with the RNAscope data and previous reports (Ji et al., 2004; Meucci et al., 2000; Mizrak et al., 2019; Voronova et al., 2017; Wang et al., 2018) and confirm that SVZ NPCs and OPCs express *Cx3cr1* and bind FKN ligand.

Surprisingly, our results indicate that OPCs express and secrete FKN at a level comparable with neurons (Voronova et al., 2017), which were previously thought to be the primary source of FKN in the brain (Luo et al., 2019). This is similar to neuroligin-3, which is secreted by both neurons and OPCs (Venkatesh et al., 2017). When endogenous FKN is sequestered, SVZ OPCs and NPCs demonstrate decreased oligodendrogenesis and increased astrogliogenesis. These results support and extend our previous report demonstrating that inhibition of FKN signaling in embryonic cortical precursors results in decreased formation of oligodendrocytes (Voronova et al., 2017). Moreover, our data indicate that the CX3CR1 signaling pathway plays a direct role in postnatal SVZ NPC and OPC fate decisions. Intriguingly, CX3CR1^{KO} mice show hippocampal-dependent behavioral deficits and decreased neurogenesis (Xiao et al., 2015; Zhan et al., 2014). Our results similarly suggest that inhibition of FKN signaling reduces SVZ NPC neurogenesis, at least *in vitro*. Whether FKN-CX3CR1 signaling plays a direct role in SGZ NPC neurogenesis and/or hippocampal OPC oligodendrogenesis remains to be addressed.

While our *in vitro* experiments indicate that FKN directly regulates SVZ NPCs and OPCs for enhanced oligodendrocyte production, our *ex vivo* and *in vivo* results do not rule out the contribution of indirect actions via FKN signaling in microglia, which express high levels of CX3CR1 (Limatola and Ransohoff, 2014). Microglia play an important role during de- and remyelination; for example, suppression of microglial activation by minocycline does not affect SVZ oligodendrogenesis in a healthy SVZ niche, but drastically decreases OLIG2$^+$ cells in a focally demyelinated SVZ (Naruse et al., 2018). Intriguingly, mice with a constitutive knockout of CX3CR1 exhibit more severe demyelination and neurological defects when subjected to cuprizone or experimental autoimmune encephalomyelitis (Garcia et al., 2013; Lampron et al., 2015). This was attributed to deficient phagocytosis of myelin debris by microglia and reduced OPC recruitment and proliferation in demyelinated lesions (Garcia et al., 2013; Lampron et al., 2015). It is tempting to speculate that at least some of these effects could be attributed to the effect of FKN on precursor cells.

Our work raises several questions in the field. Previous reports showed that pretreatment with soluble FKN prior to injury leads to neuroprotection in rodent models of stroke and Parkinson's disease as well as in an *ex vivo* model of demyelination (Cipriani et al., 2011; O'Sullivan and Dev, 2017; Pabon et al., 2011). It will be important to determine whether applying FKN after a demyelinating injury can elicit enhanced oligodendrogenesis and remyelination. A second key question involves the importance of OPC-secreted FKN in microglial function. It was recently shown that OPCs contribute to neuroinflammation and microglial activation in lipopolysaccharide-injected mice via CX3CR1 expressed on microglia (Zhang et al., 2019). Whether OPC-secreted FKN can modulate the CX3CR1 signaling pathway in microglia remains to be addressed. The third key question is whether CX3CR1 pathogenic variants detected in autism spectrum disorder, schizophrenia, and multiple sclerosis (reviewed in Watson et al., 2020)

(M). NPCs cultured with IgG (top) or αFKN (bottom) and immunostained with BCAS1 (green), GFAP (purple), or βIII (red), and counterstained with Hoechst (gray). Arrows indicate marker-positive cells.
(N) Quantification of PDGFRα$^+$ cells.
(O–R) Quantification of (M).
(S) Schematic: cerebellar slices from P10–P11 CD1 mice were cultured with αFKN or IgG.
(T) Cerebellar slices cultured with IgG (left) and αFKN (right) and immunostained for OLIG2 (green) and CC1 (red). Arrows indicate CC1$^+$OLIG2$^+$ oligodendrocytes.
(U–Y) Quantification of (T).
All graphs were analyzed with paired t test except for (Y), which was analyzed with multiple t test. Data are mean ± SEM, n = 4–6. Scale bars, 20 μm (M) and 100 μm (T). See also Figure S4.

contribute to disease pathogenesis via aberrant FKN-CX3CR1 signaling in precursor cells.

In summary, we have discovered a new role for FKN signaling in regulating postnatal and adult SVZ oligodendrogenesis. Our findings support future investigations to test the ability of FKN to recruit CNS precursor cells for enhanced oligodendrocyte genesis in injured or diseased CNS, as well as the contribution of CX3CR1 variants to the development and progression of myelin-related deficits in neurodegenerative and neurodevelopmental disorders.

EXPERIMENTAL PROCEDURES

Contact for reagent and resource sharing

Further information and requests for resources and reagents should be directed to and fulfilled by the lead contact, Anastassia Voronova (voronova@ualberta.ca).

Growth factors and function-blocking antibodies

Murine soluble FKN (R&D Systems) was added at 100, 250, and 500 ng/mL to secondary neurosphere assay; at 25, 100, 250, and 500 ng/mL to NPC monolayer cultures; and at 250 ng/mL to OPCs or cerebellar slice cultures. *In vivo*, 200 ng/day of FKN for multi-day infusion or 0.25–0.5 μg of FKN-647 (Almac) or BSA-647 (Thermo Fisher) for one-time delivery was infused into right lateral ventricle via ICV surgery. Anti-FKN (Torrey Pines Biolabs) and IgG (Jackson ImmunoResearch) controls were used at 20 μg/mL, and anti-CX3CR1 (Torrey Pines Biolabs) and IgG controls were used at 40 μg/mL.

Experimental model and subject details

Mice

Animal use protocols were approved by the Research Ethics Office at the University of Alberta in accordance with the Canadian Council of Animal Care Policies. NestinCre^{ERT2} was obtained from Imayoshi et al. (2006), RosaYFP^{STOP} and C57BL/6J from Jackson Laboratories, CD1 from Charles River, and CX3CR1^{KO} from Cardona et al. (2018). In CX3CR1^{KO} mice, human CX3CR1$^{I249/M280}$ RNA is expressed in murine Cx3cr1 locus, but is not translated until bred with Cre-recombinase-expressing mice (Cardona et al., 2018). Mice from both sexes were used for all experiments.

FKN infusion in vivo *experiments*

Two- to three-month-old WT or CX3CR1^{KO} mice were ICV-injected with FKN-647 or BSA-647 using coordinates listed below and euthanized 3 h post injection. Three-month-old NestinCre^{ERT2}; RosaYFP$^{STOP/+}$ mice were injected with tamoxifen (Sigma) for 5 days. Seventy-two hours later, ICV surgery was performed using coordinates −1.000 mediolateral, −0.300 anterior-posterior, −2.500 dorsoventral relative to bregma. FKN was infused for 7 days using osmotic mini-pumps (Alzet, 1007D).

Primary cultures

Neurospheres

SVZ was microdissected from P1–P2 or P7 CD1 pups and cultured at clonal density as primary neurospheres for 6DIV and secondary neurospheres for 7 DIV as described in Storer et al. (2018).

NPC monolayer cultures

Dissociated primary neurosphere cells were cultured for 1–5 DIV on coverslips coated with poly-D-lysine (Sigma) and laminin (VWR) in medium supplemented with 2% B27 (Invitrogen), 10 ng/mL FGF, and 20 ng/mL EGF (Peprotech). BrdU (3 μg/mL; Sigma) was added for 2 h before fixation.

OPC cultures

Dissociated primary neurosphere cells were seeded as described above in medium supplemented with 2% B27, 10 ng/mL FGF, and 10 ng/mL PDGF-AA (R&D) and cultured for 2–3 DIV. Medium conditioned by OPCs was collected and subjected to CX3CL1 ELISA (RayBiotech) (n = 3 preparations). Differentiation was induced by replacing with medium supplemented with 2% B27 and 40 ng/mL 3,3′,5-triiodo-L-thyronine (T3; Sigma).

OPC-cortical neuron co-cultures

Cells isolated from P1–P2 cortices were seeded in Neurobasal Plus medium supplemented with 2% B27 plus (Invitrogen). Cortical neurons were enriched by wiping out proliferating cells with arabinocytoside C (AraC; Sigma) incubation for 48 h. Neurons were cultured without AraC for an additional 4 days, at which point SVZ OPCs were seeded onto neurons and cultured for 2–6 DIV in the presence of 40 ng/mL T3 and FKN or VC.

Organotypic cerebellar cultures

Cerebellar slices (300 μm) isolated from P10–P11 CD1 pups were cultured using an interface method (de Almeida et al., 2021) whereby FKN/VC or anti-FKN/IgG were added at 1 DIV for 4 days.

Reagents and immunostaining

Immunocytochemistry and immunohistochemistry

Cell cultures were fixed with 4% paraformaldehyde (PFA) and subjected to FKN-647 binding assay without cell permeabilization and/or immunocytochemistry (ICC) with cell permeabilization. Mice over 21 days of age were transcardially perfused with Hank's balanced salt solution followed by 4% PFA. Mice under 21 days of age were euthanized with CO_2, and dissected brains were fixed in 4% PFA for 16–24 h. Cryosections (18 μm) were rehydrated and subjected to immunohistochemistry (IHC). Cultured cerebellar slices were fixed with 4% PFA for 1 h, followed by permeabilization and IHC. Detailed ICC and IHC protocols as well as primary and secondary antibodies are listed in supplemental information.

RNAscope

RNAscope was performed using cryosections from P2, P7, P15, and P65 CD1 brains as described in Voronova et al. (2017) with probes targeting murine *Cx3cr1*, *Olig2*, *Sox2* mRNA, or negative control probe purchased from ACD according to the manufacturer's instructions. Cells with three or more RNAscope dots were considered to be positive for marker expression.

Microscopy

RNAscope sections were imaged with a Zeiss LSM700 confocal microscope with z-stacks and an optical slice thickness of 0.2–0.5 μm. All other images were captured using a Zeiss Axio Imager M2 fluorescence microscope. Image acquisition was performed with Zen software (Zeiss). Cultured cells and *in vivo* images were imaged in single plane or with z-stacks and optical slice thickness of 0.5–1 μm.

Quantification and statistical analysis

In vitro and *ex vivo* data are from at least three independent biological experiments. At least 500–2,000 cells per treatment and biological experiment were counted. For *in vivo* experiments, 5–8 anatomically matched sections per brain were analyzed from six mice across three independent litters unless indicated otherwise. All data are presented as mean ± SEM. For two-group comparisons, two-tailed paired (*in vitro* and *ex vivo* datasets) or unpaired Student's or multiple t tests (*in vivo* datasets) were used to assess statistical significance between means. For three or more group comparisons, one-way ANOVA was followed by Dunnett's multiple comparisons test. A p value of <0.05 was considered significant (*p < 0.05, **p < 0.01, ***p < 0.001 in figures).

SUPPLEMENTAL INFORMATION

Supplemental information can be found online at https://doi.org/10.1016/j.stemcr.2021.06.010.

AUTHOR CONTRIBUTIONS

Conceptualization, A.E.S.W. and A.V.; Methodology, A.E.S.W., M.M.d.A., T.F., A.V., G.V., D.G., and A.E.C.; Formal analysis, A.E.S.W., Y.L., M.M.d.A., N.L.D., P.T., and A.V.; Investigation, A.E.S.W., Y.L., M.M.d.A., N.L.D., P.T., T.F., D.G., and A.V.; Resources, A.V. and S.S.; Writing – original draft, A.E.S.W. and A.V.; Writing – review & editing, A.E.S.W. and A.V.; Supervision, S.S. and A.V.; Funding acquisition, A.V.

DECLARATION OF INTERESTS

The authors declare no competing interests.

ACKNOWLEDGMENTS

This work was funded by NSERC (RGPIN-2018-04669), CIHR team grant (161466 under the frame of NEURON Cofund), MS Society of Canada operating grant (3573), and CRC Tier II Award awarded to A.V. A.E.S.W. was supported by the University of Alberta, MS Society of Canada (3821, 829320), and WCHRI Graduate Scholarships; Y.L. by NSERC USRAs; N.L.D. by University of Alberta awards; D.G. by research grants from Brain Canada and GlycoNet awarded to S.S. We thank Dr. Macauley for providing WT C57BL/6J mice, Kara Goodkey and Beatrix Wang for technical assistance, and Drs. Allison and Hughes for reading the manuscript.

Received: December 2, 2020
Revised: June 14, 2021
Accepted: June 15, 2021
Published: July 15, 2021

REFERENCES

Bachstetter, A.D., Morganti, J.M., Jernberg, J., Schlunk, A., Mitchell, S.H., Brewster, K.W., Hudson, C.E., Cole, M.J., Harrison, J.K., Bickford, P.C., et al. (2011). Fractalkine and CX 3 CR1 regulate hippocampal neurogenesis in adult and aged rats. Neurobiol. Aging *32*, 2030–2044. https://doi.org/10.1016/j.neurobiolaging.2009.11.022.

Barak, B., Zhang, Z., Liu, Y., Nir, A., Trangle, S.S., Ennis, M., Levandowski, K.M., Wang, D., Quast, K., Boulting, G.L., et al. (2019). Neuronal deletion of Gtf2i, associated with Williams syndrome, causes behavioral and myelin alterations rescuable by a remyelinating drug. Nat. Neurosci. *22*, 700–708. https://doi.org/10.1038/s41593-019-0380-9.

Belenguer, G., Duart-Abadia, P., Jordán-Pla, A., Domingo-Muelas, A., Blasco-Chamarro, L., Ferrón, S.R., Morante-Redolat, J.M., and Fariñas, I. (2021). Adult neural stem cells are alerted by systemic inflammation through TNF-α receptor signaling. Cell Stem Cell *28*, 285–299.e289. https://doi.org/10.1016/j.stem.2020.10.016.

Bhat, R.V., Axt, K.J., Fosnaugh, J.S., Smith, K.J., Johnson, K.A., Hill, D.E., Kinzler, K.W., and Baraban, J.M. (1996). Expression of the APC tumor suppressor protein in oligodendroglia. Glia *17*, 169–174. https://doi.org/10.1002/(sici)1098-1136.

Cardona, S.M., Kim, S.V., Church, K.A., Torres, V.O., Cleary, I.A., Mendiola, A.S., Saville, S.P., Watowich, S.S., Parker-Thornburg, J., Soto-Ospina, A., et al. (2018). Role of the fractalkine receptor in CNS autoimmune inflammation: new approach utilizing a mouse model expressing the human CX3CR1(I249/m280) variant. Front. Cell. Neurosci. *12*, 365. https://doi.org/10.3389/fncel.2018.00365.

Cipriani, R., Villa, P., Chece, G., Lauro, C., Paladini, A., Micotti, E., Perego, C., De Simoni, M.G., Fredholm, B.B., Eusebi, F., et al. (2011). CX3CL1 is neuroprotective in permanent focal cerebral ischemia in rodents. J. Neurosci. *31*, 16327–16335. https://doi.org/10.1523/jneurosci.3611-11.2011.

de Almeida, M.M.A., Pieropan, F., Footz, T., David, J.M., David, J.P., da Silva, V.D.A., Dos Santos Souza, C., Voronova, A., Butt, A.M., and Costa, S.L. (2021). Agathisflavone modifies microglial activation state and myelination in organotypic cerebellar slices culture. J. Neuroimmune Pharmacol. https://doi.org/10.1007/s11481-021-09991-6.

Fard, M.K., van der Meer, F., Sánchez, P., Cantuti-Castelvetri, L., Mandad, S., Jäkel, S., Fornasiero, E.F., Schmitt, S., Ehrlich, M., Starost, L., et al. (2017). BCAS1 expression defines a population of early myelinating oligodendrocytes in multiple sclerosis lesions. Sci. Transl. Med. *9*. https://doi.org/10.1126/scitranslmed.aam7816.

Finneran, D.J., Morgan, D., Gordon, M.N., and Nash, K.R. (2019). CNS-wide over expression of fractalkine improves cognitive functioning in a tauopathy model. J. Neuroimmune Pharmacol. *14*, 312–325. https://doi.org/10.1007/s11481-018-9822-5.

Fujita, M., Takada, Y.K., and Takada, Y. (2012). Integrins αvβ3 and α4β1 act as coreceptors for fractalkine, and the integrin-binding defective mutant of fractalkine is an antagonist of CX3CR1. J. Immunol. *189*, 5809–5819. https://doi.org/10.4049/jimmunol.1200889.

Garcia, J.A., Pino, P.A., Mizutani, M., Cardona, S.M., Charo, I.F., Ransohoff, R.M., Forsthuber, T.G., and Cardona, A.E. (2013). Regulation of adaptive immunity by the fractalkine receptor during autoimmune inflammation. J. Immunol. *191*, 1063–1072. https://doi.org/10.4049/jimmunol.1300040.

Imayoshi, I., Ohtsuka, T., Metzger, D., Chambon, P., and Kageyama, R. (2006). Temporal regulation of Cre recombinase activity in neural stem cells. Genesis *44*, 233–238. https://doi.org/10.1002/dvg.20212.

Ishimoto, T., Ninomiya, K., Inoue, R., Koike, M., Uchiyama, Y., and Mori, H. (2017). Mice lacking BCAS1, a novel myelin-associated protein, display hypomyelination, schizophrenia-like abnormal behaviors, and upregulation of inflammatory genes in the brain. Glia *65*, 727–739. https://doi.org/10.1002/glia.23129.

Ji, J.F., He, B.P., Dheen, S.T., and Tay, S.S. (2004). Expression of chemokine receptors CXCR4, CCR2, CCR5 and CX3CR1 in neural progenitor cells isolated from the subventricular zone of the adult rat brain. Neurosci. Lett. *355*, 236–240. https://doi.org/10.1016/j.neulet.2003.11.024.

Krathwohl, M.D., and Kaiser, J.L. (2004). Chemokines promote quiescence and survival of human neural progenitor cells. Stem Cells *22*, 109–118. https://doi.org/10.1634/stemcells.22-1-109.

Lampron, A., Larochelle, A., Laflamme, N., Prefontaine, P., Plante, M.M., Sanchez, M.G., Yong, V.W., Stys, P.K., Tremblay, M.E., and Rivest, S. (2015). Inefficient clearance of myelin debris by microglia impairs remyelinating processes. J. Exp. Med. *212*, 481–495. https://doi.org/10.1084/jem.20141656.

Limatola, C., and Ransohoff, R.M. (2014). Modulating neurotoxicity through CX3CL1/CX3CR1 signaling. Front. Cell. Neurosci. *8*, 229. https://doi.org/10.3389/fncel.2014.00229.

Luo, P., Chu, S.F., Zhang, Z., Xia, C.Y., and Chen, N.H. (2019). Fractalkine/CX3CR1 is involved in the cross-talk between neuron and glia in neurological diseases. Brain Res. Bull. *146*, 12–21. https://doi.org/10.1016/j.brainresbull.2018.11.017.

Maki, T., Liang, A.C., Miyamoto, N., Lo, E.H., and Arai, K. (2013). Mechanisms of oligodendrocyte regeneration from ventricular-subventricular zone-derived progenitor cells in white matter diseases. Front. Cell. Neurosci. *7*, 275. https://doi.org/10.3389/fncel.2013.00275.

Marques, S., van Bruggen, D., Vanichkina, D.P., Floriddia, E.M., Munguba, H., Väremo, L., Giacomello, S., Falcão, A.M., Meijer, M., Björklund, Å.K., et al. (2018). Transcriptional convergence of oligodendrocyte lineage progenitors during development. Dev. Cell *46*, 504–517.e507. https://doi.org/10.1016/j.devcel.2018.07.005.

McKenzie, I.A., Ohayon, D., Li, H., de Faria, J.P., Emery, B., Tohyama, K., and Richardson, W.D. (2014). Motor skill learning requires active central myelination. Science *346*, 318–322. https://doi.org/10.1126/science.1254960.

Menn, B., Garcia-Verdugo, J.M., Yaschine, C., Gonzalez-Perez, O., Rowitch, D., and Alvarez-Buylla, A. (2006). Origin of oligodendrocytes in the subventricular zone of the adult brain. J. Neurosci. *26*, 7907–7918. https://doi.org/10.1523/jneurosci.1299-06.2006.

Meucci, O., Fatatis, A., Simen, A.A., and Miller, R.J. (2000). Expression of CX3CR1 chemokine receptors on neurons and their role in neuronal survival. Proc. Natl. Acad. Sci. U S A *97*, 8075–8080. https://doi.org/10.1073/pnas.090017497.

Mizrak, D., Levitin, H.M., Delgado, A.C., Crotet, V., Yuan, J., Chaker, Z., Silva-Vargas, V., Sims, P.A., and Doetsch, F. (2019). Single-cell analysis of regional differences in adult V-SVZ neural stem cell lineages. Cell Rep. *26*, 394–406.e395. https://doi.org/10.1016/j.celrep.2018.12.044.

Murphy, N.A., and Franklin, R.J.M. (2017). Recruitment of endogenous CNS stem cells for regeneration in demyelinating disease. Prog. Brain Res. *231*, 135–163. https://doi.org/10.1016/bs.pbr.2016.12.013.

Naruse, M., Shibasaki, K., Shimauchi-Ohtaki, H., and Ishizaki, Y. (2018). Microglial activation induces generation of oligodendrocyte progenitor cells from the subventricular zone after focal demyelination in the corpus callosum. Dev. Neurosci. *40*, 54–63. https://doi.org/10.1159/000486332.

Nash, K.R., Moran, P., Finneran, D.J., Hudson, C., Robinson, J., Morgan, D., and Bickford, P.C. (2015). Fractalkine over expression suppresses α-Synuclein-mediated neurodegeneration. Mol. Ther. *23*, 17–23. https://doi.org/10.1038/mt.2014.175.

O'Sullivan, S.A., and Dev, K.K. (2017). The chemokine fractalkine (CX3CL1) attenuates H_2O_2-induced demyelination in cerebellar slices. J. Neuroinflammation *14*, 159. https://doi.org/10.1186/s12974-017-0932-4.

Obernier, K., and Alvarez-Buylla, A. (2019). Neural stem cells: origin, heterogeneity and regulation in the adult mammalian brain. Development *146*. https://doi.org/10.1242/dev.156059.

Pabon, M.M., Bachstetter, A.D., Hudson, C.E., Gemma, C., and Bickford, P.C. (2011). CX3CL1 reduces neurotoxicity and microglial activation in a rat model of Parkinson's disease. J. Neuroinflammation *8*, 9. https://doi.org/10.1186/1742-2094-8-9.

Pan, S., Mayoral, S.R., Choi, H.S., Chan, J.R., and Kheirbek, M.A. (2020). Preservation of a remote fear memory requires new myelin formation. Nat. Neurosci. *23*, 487–499. https://doi.org/10.1038/s41593-019-0582-1.

Schneider, S., Gruart, A., Grade, S., Zhang, Y., Kroger, S., Kirchhoff, F., Eichele, G., Delgado Garcia, J.M., and Dimou, L. (2016). Decrease in newly generated oligodendrocytes leads to motor dysfunctions and changed myelin structures that can be rescued by transplanted cells. Glia *64*, 2201–2218. https://doi.org/10.1002/glia.23055.

Shen, K., and Yuen, T.J. (2020). Ex vivo myelination and remyelination in cerebellar slice cultures as a quantitative model for developmental and disease-relevant manipulations. J. Vis. Exp. https://doi.org/10.3791/61044.

Silva-Vargas, V., Maldonado-Soto, A.R., Mizrak, D., Codega, P., and Doetsch, F. (2016). Age-dependent niche signals from the choroid plexus regulate adult neural stem cells. Cell Stem Cell *19*, 643–652. https://doi.org/10.1016/j.stem.2016.06.013.

Steadman, P.E., Xia, F., Ahmed, M., Mocle, A.J., Penning, A.R.A., Geraghty, A.C., Steenland, H.W., Monje, M., Josselyn, S.A., and Frankland, P.W. (2019). Disruption of oligodendrogenesis impairs memory consolidation in adult mice. Neuron https://doi.org/10.1016/j.neuron.2019.10.013.

Storer, M.A., Gallagher, D., Fatt, M.P., Simonetta, J.V., Kaplan, D.R., and Miller, F.D. (2018). Interleukin-6 regulates adult neural stem cell numbers during normal and abnormal post-natal development. Stem Cell Rep. *10*, 1464–1480. https://doi.org/10.1016/j.stemcr.2018.03.008.

Suzuki, N., Sekimoto, K., Hayashi, C., Mabuchi, Y., Nakamura, T., and Akazawa, C. (2017). Differentiation of oligodendrocyte precursor cells from sox10-venus mice to oligodendrocytes and

astrocytes. Sci. Rep. *7*, 14133. https://doi.org/10.1038/s41598-017-14207-0.

Venkatesh, H.S., Tam, L.T., Woo, P.J., Lennon, J., Nagaraja, S., Gillespie, S.M., Ni, J., Duveau, D.Y., Morris, P.J., Zhao, J.J., et al. (2017). Targeting neuronal activity-regulated neuroligin-3 dependency in high-grade glioma. Nature *549*, 533–537. https://doi.org/10.1038/nature24014.

Voronova, A., Yuzwa, S.A., Wang, B.S., Zahr, S., Syal, C., Wang, J., Kaplan, D.R., and Miller, F.D. (2017). Migrating interneurons secrete fractalkine to promote oligodendrocyte formation in the developing mammalian brain. Neuron *94*, 500–516.e509. https://doi.org/10.1016/j.neuron.2017.04.018.

Wang, D.D., Tian, T., Dong, Q., Xu, X.F., Yu, H., Wang, Y., and Chen, Z.Y. (2014). Transcriptome profiling analysis of the mechanisms underlying the BDNF Val66Met polymorphism induced dysfunctions of the central nervous system. Hippocampus *24*, 65–78. https://doi.org/10.1002/hipo.22204.

Wang, F., Ren, S.-Y., Chen, J.-F., Liu, K., Li, R.-X., Li, Z.-F., Hu, B., Niu, J.-Q., Xiao, L., Chan, J.R., et al. (2020). Myelin degeneration and diminished myelin renewal contribute to age-related deficits in memory. Nat. Neurosci. *23*, 481–486. https://doi.org/10.1038/s41593-020-0588-8.

Wang, J., Gan, Y., Han, P., Yin, J., Liu, Q., Ghanian, S., Gao, F., Gong, G., and Tang, Z. (2018). Ischemia-induced neuronal cell death is mediated by chemokine receptor CX3CR1. Sci. Rep. *8*, 556. https://doi.org/10.1038/s41598-017-18774-0.

Watson, A.E.S., Goodkey, K., Footz, T., and Voronova, A. (2020). Regulation of CNS precursor function by neuronal chemokines. Neurosci. Lett. *715*, 134533. https://doi.org/10.1016/j.neulet.2019.134533.

Xiao, F., Xu, J.M., and Jiang, X.H. (2015). CX3 chemokine receptor 1 deficiency leads to reduced dendritic complexity and delayed maturation of newborn neurons in the adult mouse hippocampus. Neural Regen. Res. *10*, 772–777. https://doi.org/10.4103/1673-5374.156979.

Xiao, L., Ohayon, D., McKenzie, I.A., Sinclair-Wilson, A., Wright, J.L., Fudge, A.D., Emery, B., Li, H., and Richardson, W.D. (2016). Rapid production of new oligodendrocytes is required in the earliest stages of motor-skill learning. Nat. Neurosci. *19*, 1210–1217. https://doi.org/10.1038/nn.4351.

Xing, Y.L., Röth, P.T., Stratton, J.A., Chuang, B.H., Danne, J., Ellis, S.L., Ng, S.W., Kilpatrick, T.J., and Merson, T.D. (2014). Adult neural precursor cells from the subventricular zone contribute significantly to oligodendrocyte regeneration and remyelination. J. Neurosci. *34*, 14128–14146. https://doi.org/10.1523/jneurosci.3491-13.2014.

Zhan, Y., Paolicelli, R.C., Sforazzini, F., Weinhard, L., Bolasco, G., Pagani, F., Vyssotski, A.L., Bifone, A., Gozzi, A., Ragozzino, D., et al. (2014). Deficient neuron-microglia signaling results in impaired functional brain connectivity and social behavior. Nat. Neurosci. *17*, 400–406. https://doi.org/10.1038/nn.3641.

Zhang, S.Z., Wang, Q.Q., Yang, Q.Q., Gu, H.Y., Yin, Y.Q., Li, Y.D., Hou, J.C., Chen, R., Sun, Q.Q., Sun, Y.F., et al. (2019). NG2 glia regulate brain innate immunity via TGF-beta2/TGFBR2 axis. BMC Med. *17*, 204. https://doi.org/10.1186/s12916-019-1439-x.

Stem Cell Reports, Volume *16*

Supplemental Information

Fractalkine signaling regulates oligodendroglial cell genesis from SVZ precursor cells

Adrianne E.S. Watson, Monique M.A. de Almeida, Nicole L. Dittmann, Yutong Li, Pouria Torabi, Tim Footz, Gisella Vetere, Danny Galleguillos, Simonetta Sipione, Astrid E. Cardona, and Anastassia Voronova

Fig. S1, related to Fig. 1

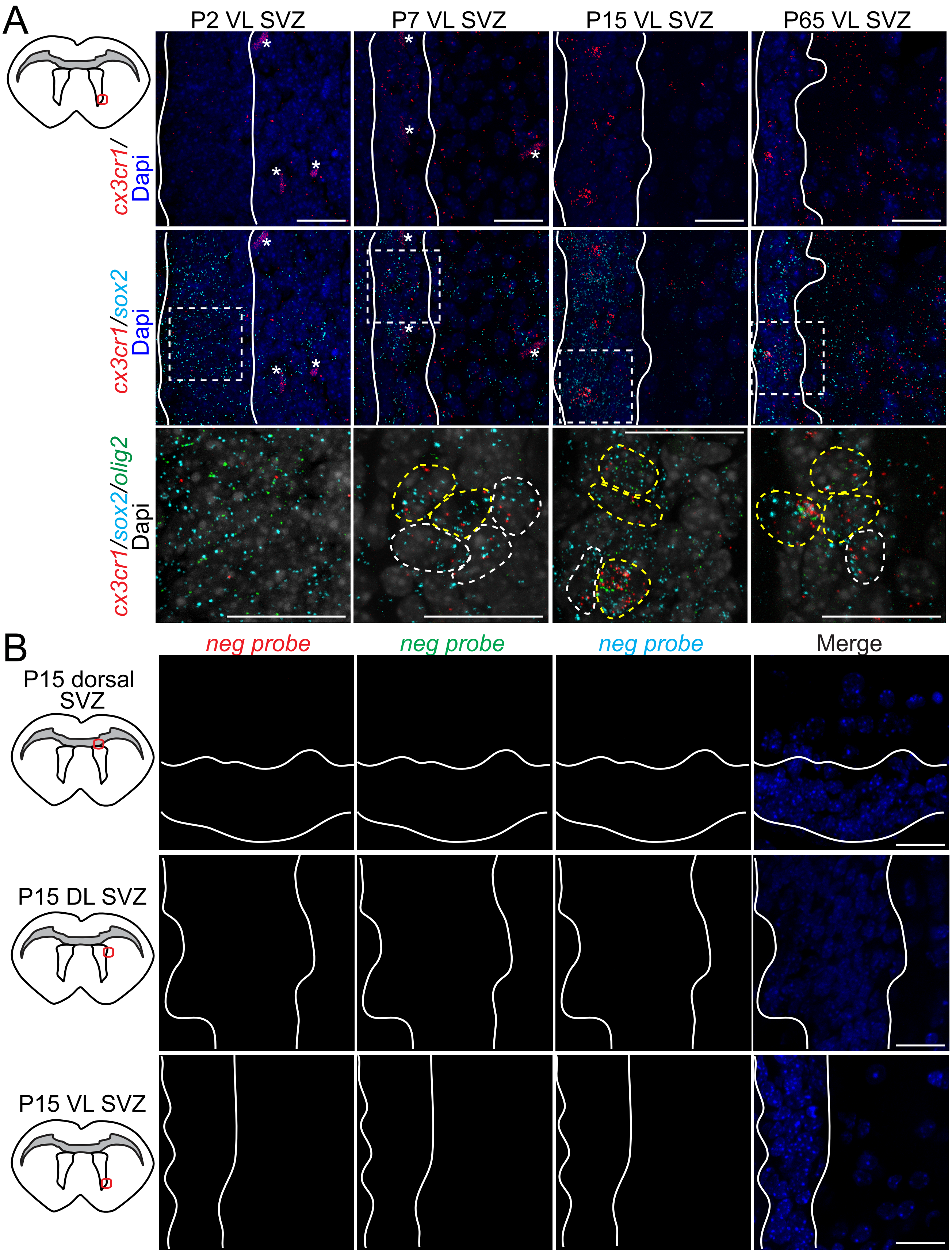

Figure S1. NPCs and OPCs express *Cx3cr1* mRNA in the ventrolateral SVZ. Related to Fig. 1. A. Top left: Schematic of coronal brain section, approximate location of ventrolateral (VL) SVZ indicated by red circle. Top row: RNA Scope analysis of VL SVZ from P2, P7, P15, and P65 for *Cx3cr1* (red) mRNA. Middle row: RNA Scope analysis of VL SVZ from P2, P7, P15, and P65 (left-right) for *Cx3cr1* (red) and *Sox2* (cyan) mRNAs. Hatched boxes indicate section shown in higher magnification in bottom row. Bottom row: Higher magnification of hatched boxes in middle rows. RNA scope analysis of VL SVZ from P2, P7, P15, and P65 for *Cx3cr1* (red), *Sox2* (cyan), and *Olig2* (green) mRNAs. White dashed circles indicate *Cx3cr1*$^{+}$*SOX2*$^{+}$ cells, and yellow dashed circles *Cx3cr1*$^{+}$*Sox2*$^{+}$*Olig2*$^{+}$ cells. **B**. RNA Scope analysis of P15 dorsal (top), dorsolateral (DL, middle), and ventrolateral (VL, bottom) SVZ from P15 mice with negative probe. Left: schematic of coronal brain section with approximate location captured, indicated by red circle. For all images cells were counterstained with Dapi (blue or grey). Solid lines indicate SVZ boundaries. Asterisks denote blood vessels. Scale bars are 20μm. A cell was considered positive for *Cx3cr1*, *Sox2* and/or *Olig2* if it contained at least 3 RNA Scope signal dots for respective mRNA. n=2-3 for each age.

Fig. S2, related to Fig. 2

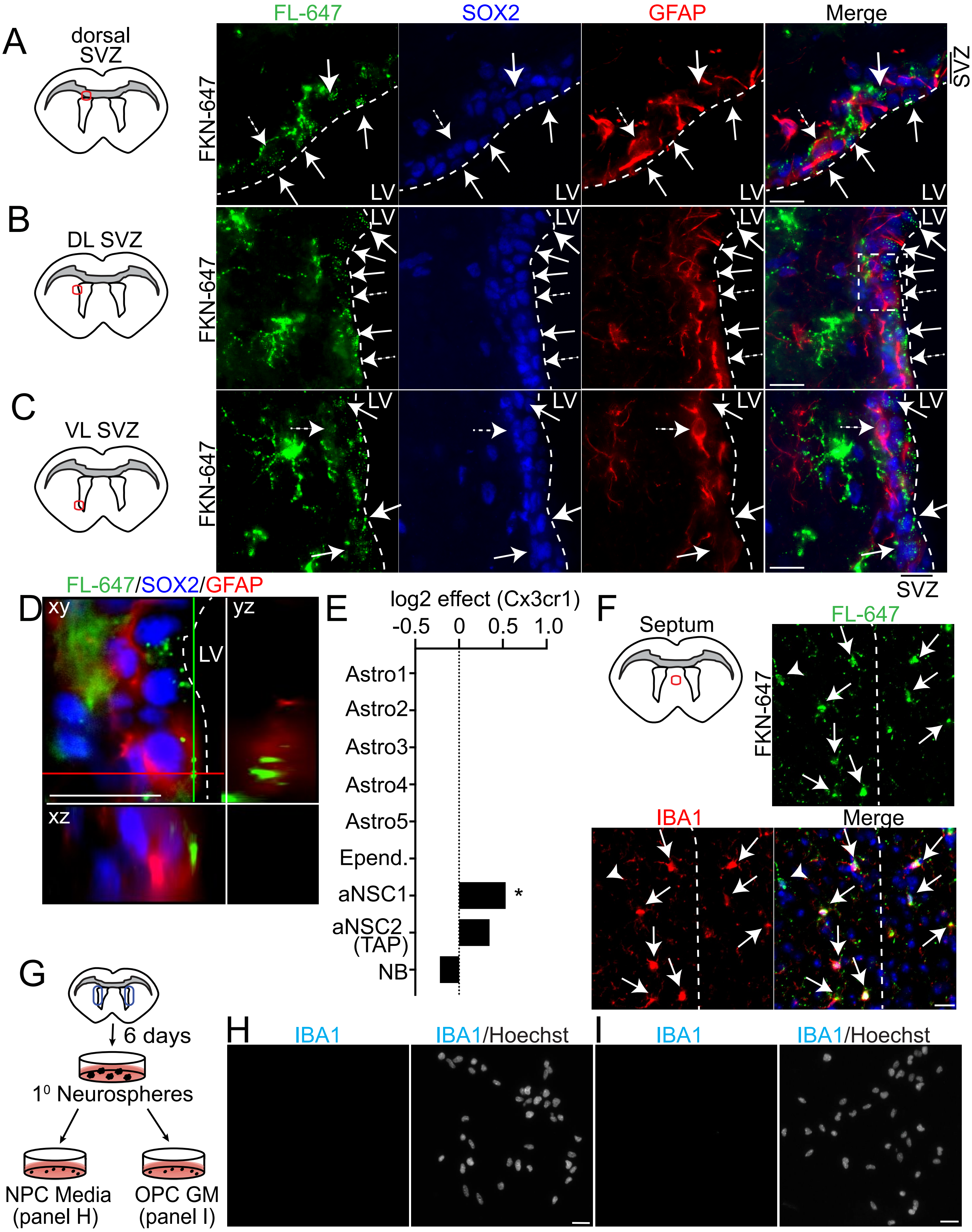

Figure S2. FKN binds SVZ NPCs *in vivo*. Related to Fig. 2. A-C. 2-3-month old WT mice were injected once with FKN-647 into the right lateral ventricle via ICV surgery and euthanized 3-hours post injection. **A-C.** Left: schematic of coronal section showing approximate location of dorsal **(A)**, dorsolateral (DL) **(B)** and ventrolateral (VL) **(C)** SVZ is indicated by red circle. Sections were immunostained with NPC markers SOX2 (blue, second column) and GFAP (red, third column). Fluorescence in far-red channel (FL-647) is pseudo-colored in green (left column). Solid arrows indicate FKN-647^+SOX2^+GFAP$^+$ cells. Dashed arrows indicate FKN-647^+ SOX2^+GFAP$^-$ cells. Dashed line indicates SVZ boundary and LV indicates lateral ventricle. Hatched box in “merge” panel in **B** is shown at higher magnification in **D**. **D.** Orthogonal slice through Z-stack of FKN-647^+SOX2^+GFAP$^+$ cells in DL SVZ demonstrating FKN-647 signal is detected on the surface of NPCs. On xy projection (left), green line indicates location of yz plane projection (right). Red line indicates location of xz projection (bottom). Dashed line indicates SVZ boundary and LV indicates lateral ventricle. **E.** *Cx3cr1* mRNA expression enrichment in SVZ cell subclusters. Data was extracted from single cell RNA sequencing binomial specific analysis ([1] and Table S2 within). * FDR <0.05. Astro = non-proliferating quiescent SVZ neural stem cell (NSC), Epend. = ependymal, aNSC = proliferating activated NSC, TAP = transit amplifying progenitor, NB = neuroblast. **F.** Top left corner: schematic of coronal section showing approximate location of septum is indicated by red circle. Top right corner and bottom row: representative image of FKN-647 infused WT mouse immunostained with IBA1 (red). Fluorescence in far-red channel (FL-647) is pseudo-colored in green. Solid arrows indicate FKN-647^+IBA1^+ cells. Arrowhead indicates FKN-647^+IBA1^- cell. Sections were counterstained with Hoechst 33258 (blue). **G.** Schematic illustration of SVZ NPC and OPC cultures. **H-I.** Neurosphere cells were cultured in NPC media for 1 DIV **(H)** or OPC GM for 2 DIV **(I)**, immunostained for IBA1 (cyan), and counterstained with Hoechst 33258 (grey). Scale bars are 20 μm.

Fig. S3
related to Fig. 6

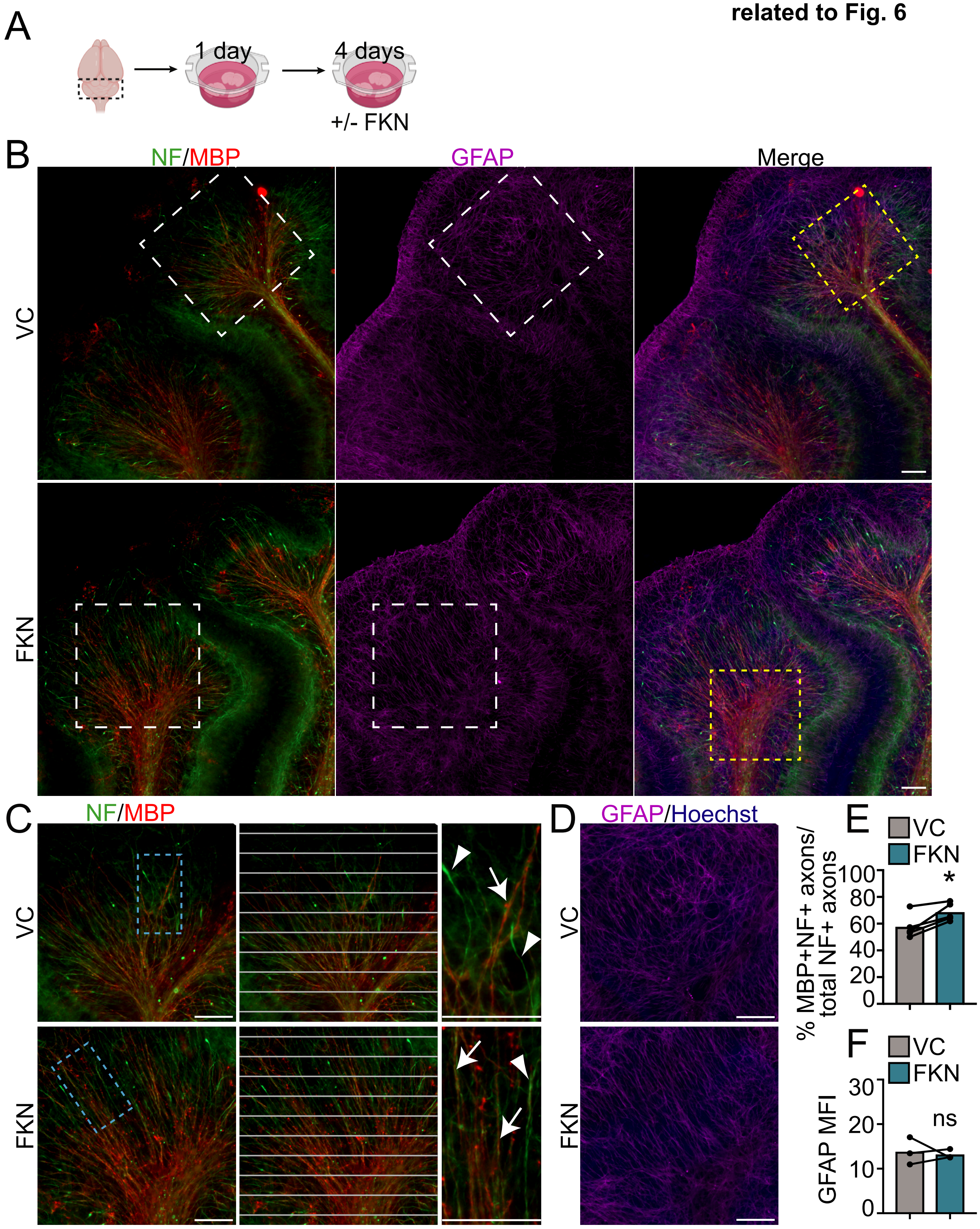

Figure S3: FKN increases proportion of myelinated axons in cerebellar *ex vivo* slices. Related to Fig. 6. A. Schematic: cerebellar slices from P10-11 CD1 mice were allowed to adhere in culture for 1 day, followed by 4-day incubation with 250 ng/ml FKN or VC (PBS). **B.** Representative image of cerebellar slice cultured with VC (top) or FKN (bottom) and immunostained for neurofilament (NF, green), MBP (red), and GFAP (purple). Nuclei were visualized with Hoechst (blue in Merge panels). Hatched white boxes are shown in higher magnification in **C.** Hatched yellow boxes in Merge panels are representative of deep white cerebellar white matter analyzed in Fig. 6M-R. **C**. Higher magnification of cerebellar slice cultured with VC (top) or FKN (bottom) and immunostained for NF (green) and MBP (red). Hatched blue boxes in left column are shown at higher magnification on right. Middle column: lines indicate grid used to quantify $MBP^{+}NF^{+}$ and total NF^{+} fibre intersections with each grid (used to calculate proportion of myelinated axons relative to total axons). Right column: higher magnification of left column. Arrows indicate $MBP^{+}NF^{+}$ axons, and arrowheads indicate $MBP^{-}NF^{+}$ axons. **D.** Higher magnification of cerebellar slice cultured with VC (top) or FKN (bottom) and immunostained for GFAP (purple) and counterstained with Hoechst 33258 (blue). **E.** Quantification of **C**. *$p<0.05$. n=5 biological replicates with at least 2 slices analyzed per replicate. At least 600 NF intersections were counted for each replicate. **F.** Quantification of mean fluorescent intensity (MFI) in **D.** ns=not significant. n=3 biological replicates with at least 3 slices analyzed per replicate. All scale bars: 100μm. Graphs were analyzed with student's paired t-test.

Fig. S4
related to Fig. 7

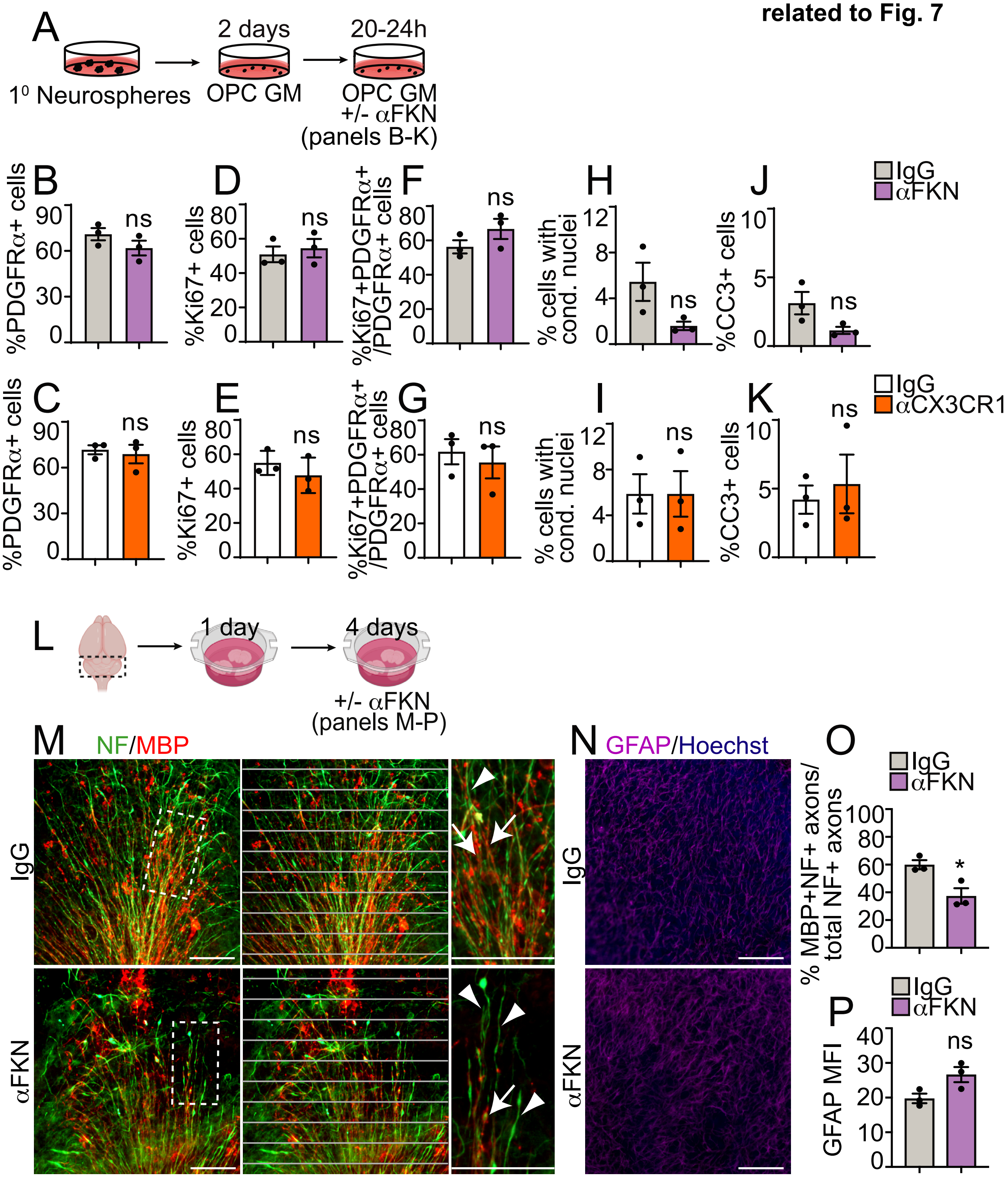

Figure S4. Inhibition of FKN signaling does not affect OPC proliferation, but reduces proportion of myelinated axons in cerebellar *ex vivo* slices. Related to Fig. 7. A. Schematic: P7 SVZ primary neurosphere cells were cultured as monolayers in OPC GM for 2DIV, followed by 20-24 hours in OPC GM with αFKN (20 μg/ml), αCX3CR1 (40 μg/ml) or IgG (20 or 40 μg/ml). **B-K.** Quantification of PDGFRα^{+} cells **(B-C)**, Ki67^{+} cells **(D-E)**, proliferation index of PDGFRα^{+} cells **(F-G)**, cells with condensed nuclei **(H-I)**, and CC3^{+} cells **(J-K)** cultured with αFKN (**D, D, F, H, J**) or αCX3CR1 (**C, E, G, I, K**). Marker^{+} cells were expressed as % of healthy Hoechst^{+} cells. Proliferation index is expressed as %Ki67^{+}marker^{+} cells relative to total marker^{+} cells. Cells with condensed nuclei expressed as % of total Hoechst^{+} cells **L.** Schematic: cerebellar slices from P10-11 CD1 mice were allowed to adhere in culture for 1 day, followed by a 4-day incubation with αFKN or IgG (20 μg/ml). **M.** Representative image of cerebellar slice cultured with IgG (top) or αFKN (bottom) and immunostained for NF (green) and MBP (red). Hatched boxes in left column are shown in higher magnification on right. Middle column: lines indicate grid used to quantify MBP^{+}NF^{+} and total NF^{+} fibre intersections with each grid (used to calculate proportion of myelinated axons relative to total axons). Right column: higher magnification of left column. Arrows indicate MBP^{+}NF^{+} axons, and arrowheads indicate MBP^{-}NF^{+} axons. **N.** Representative image of cerebellar slice cultured with VC (top) or FKN (bottom) immunostained for GFAP (purple) and counterstained with Hoechst 33258 (blue). **O.** Quantification of **M**. **P.** Quantification of mean fluorescent intensity (MFI) in **N.** All scale bars: 100 μm. Graphs were analyzed with student's paired t-test. * $p<0.05$, ns=not significant. n=3 biological replicates.

Supplemental Experimental Procedures

MATERIALS AND METHODS

Growth factors and function-blocking antibodies: Murine soluble FKN (CX3CL1) was obtained from R&D Systems (cat #458-MF), FKN-647 from Almac (cat #CAF-51-A-01, #CAF-14-A-03). BSA-647 was obtained from ThermoFisher (cat# A34785). Cy5-Streptavidin was obtained from Jackson ImmunoResearch (cat# 016-170-084). For cell and slice culture experiments, FKN was reconstituted in sterile 1X PBS at a concentration of 100 µg/mL. FKN was added at 100, 250 and 500 ng/ml to secondary neurosphere assay; at 25, 100, 250 and 500 ng/ml to NPC monolayer cultures (please see "Primary cell and *ex vivo* cerebellar slice cultures" section below). FKN was added at 250 ng/ml to OPCs cultured in OPC growth (GM) or differentiation (DM) media or to cerebellar slice cultures. For FKN intracerebral ventricular (ICV) surgery, FKN was reconstituted at 16.7 ng/mL in sterile 0.2% bovine serum albumin (BSA, Jackson ImmunoResearch) in 1x PBS. FKN-647 was reconstituted at 0.5 mg/mL in 0.1% BSA in 1X PBS. Function-blocking antibody specific for FKN (cat #TP233) and CX3CR1 (cat #TP501) was obtained from Torrey Pines Biolabs. Non-specific rabbit IgG was obtained from Jackson ImmunoResearch. Anti-FKN and IgG control were used at 20 μg/ml and anti-CX3CR1 and IgG control were used at 40 μg/ml as per (2). Anti-FKN and anti-CX3CR1 are validated to block FKN signalling as per (2-5).

Experimental Model And Subject Details

Mice: Animal use protocols were approved by the Research Ethics Office at the University of Alberta in accordance with the Canadian Council of Animal Care Policies. Mice from both sexes were used for all cell culture and *in vivo* experiments. NestinCre^{ERT2} (C57BL/6-Tg(Nes-cre/ERT2)) was obtained from (6). RosaYFP^{STOP} (B6.129X1-Gt(ROSA)26Sortm1(EYFP)Cos/J; RRID:IMSR_JAX:006148) and wild-type (WT) C57BL/6J mice were obtained from Jackson Laboratories. CX3CR1 knockout (KO) mice, in which human CX3CR1 with I249/M280 mutation was knocked into mouse CX3CR1 locus (hCX3CR1$^{I249/M280fl/fl}$) were obtained from (7). In these mice, hCX3CR1$^{I249/M280}$ RNA is expressed in murine Cx3cr1 locus, but is not translated until bred with Cre-recombinase mice (7). For primary cell culture experiments, CX3CR1^{KO} (7) or CD1 mice purchased from Charles River Laboratory were used for all primary cell culture experiments (developmental age: postnatal day [P] 1-2, 7, slice culture [P10-11]). For RNA scope, P2, P7, P15. and P65 mice were used. For NPC lineage tracing experiments and FKN-647 infusion, 2-3-month old animals were used.

For genotyping, the following primers were used: NestinCre^{ERT2}: TTCCGCTGGGTCACTGTCGCCGCTAC (5'-3', forward), TAATCGCGAACATCTTCAGGTTCTGC (5'-3', reverse), RosaYFP^{STOP}: AAAGTCGCTCTGAGTTGTTAT (5'-3', common), GGAGCGGGAGAAATGGATATG (5'-3', wildtype), GGAGCGGGAGAAATGGATATG, (5'-3', mutant), CX3CR1^{KO}: TACCTGGCCATCGTCCTGGCCGCCA (5'-3', forward), ACGAGACTAGTGAGACGTGCTACTT (5'-3', reverse); CX3CR1^{WT}: GTCTTCACGTTCGGTCTGGT (5'-3', forward), CCCAGACACTCGTTGTCCTT (5'-3', reverse).

FKN infusion *in vivo* experiments: For FKN-647 infusion experiments (Fig. 2A-G, Fig. S2A-D, F), 2-3-month old wild-type C57/BL6J or CX3CR1^{KO} mice were used. For FKN mini-pump infusion experiments (Fig. 6A-L), NestinCre^{ERT2}-positive males were bred with RosaYFP$^{STOP/STOP}$-positive females. Resulting Cre-positive, RosaYFP$^{STOP/+}$ 3-month old progeny were injected with 3 mg tamoxifen dissolved in 10% ethanol (Commercial Alcohols) and 90% sunflower seed oil (Sigma) daily for 5 days. 72h after the last tamoxifen injection, ICV surgery was performed. For ICV surgery, mice were anesthetized via inhalation of isoflurane and placed in a stereotaxic frame. Syringe needle (for one-time injection) or cannulas (Alzet, for multi-day infusion) were positioned after craniotomy using the following coordinates relative to bregma: -1.000 medio-lateral, -0.300 anterior-posterior, -2.500 dorso-ventral for infusion into right ventricle. For one-time injection, 0.5-1 µl of FKN-647 or matched volume and equimolar amount of BSA-647 or Cy5-Streptavidin were injected once over a 10-20 min period. For multi-day infusion, 7-day osmotic mini-pumps (Alzet, 1007D) were connected containing VC or 16.7 ng/ml FKN. Overall, 200 ng of FKN was delivered over 24h for 7 days. Cannula was secured to the skull with Loctite 454. After 3h (with one-time injection) or 7 days (with mini-pump infusions), mice were anesthetized with 102 mg/kg of body weight Euthansol (Western Drug Distribution Center Limited, WDDC). Mice were then perfused transcardially with Hank's Balanced Salt Solution (HBSS, Gibco), followed by 4% paraformaldehyde in 1X PBS (PFA, Acros Organics). Brains were post-fixed in 4% PFA for additional 24 hours, after which brains

were cryopreserved in 30% sucrose (Fisher) in 1X PBS for 72 hours. Brains were embedded in optimal cutting temperature (O.C.T) compound (Thermo Scientific™ Shandon™ Cryomatrix™) and flash frozen for downstream assays.

Primary cell and ex vivo cerebellar slice cultures:

Primary Neurospheres: Subventricular zone (SVZ) tissue was microdissected from P1-2 or P7 CD1 pups of either sex. Primary neurospheres were cultured as described in (8) at 10 cells/µL. Briefly, cells were cultured in Serum-Free Media (SFM; Dulbecco's Modified Eagle Medium (low glucose) (DMEM, Gibco), Ham's F-12 Nutrient Mixture (F12, Gibco), 0.6% glucose (Sigma), 0.1125% Sodium Bicarbonate ($NaHCO_3$, Gibco), 5 mM N-2-hydroxyethylpiperazine-N-2-ethane sulfonic acid (HEPES, Gibco), 100 µg/mL L-glutamine (Lonza), 1% Penicillin-Streptomycin (Pen/Strep, Lonza) supplemented with 2% B27 supplement (Life Technologies), 10 ng/ml Fibroblast Growth Factor (FGF, Peprotech), 20 ng/ml Epidermal Growth Factor (EGF, Peprotech), and 2 µg/ml heparin sodium salt (Sigma) (herein referred to as **Neurosphere Media**). After 6 days, neurospheres were collected, dissociated, and plated for downstream assays as outlined below.

Secondary Neurospheres: Dissociated primary neurosphere cells cultured from P7 SVZ were seeded at clonal density of 2 cells/µL in Neurosphere Media for 7 days. Each sphere containing at least 50 cells was counted on 7DIV (days *in vitro*) as per (9).

NPC Monolayer Cultures: Dissociated primary neurosphere cells were seeded at 39,500 cells/cm^2 on plates coated with 40 µg/ml Poly-D-Lysine (Sigma) and 4 µg/ml laminin (Corning) in SFM supplemented with 2% B27, 10 ng/ml FGF and 20 ng/ml EGF and cultured for 1-5DIV. For proliferation assays, BrdU (Bromodeoxyuridine [5-bromo-2'-deoxyuridine], Sigma) was added at 3 µg/ml for 2h before fixing the cultures.

OPC Cultures: Dissociated primary neurosphere cells were seeded at 39,500-47,000 cells/cm^2 on plates coated with Poly-D-Lysine and laminin in SFM supplemented with 2% B27, 10 ng/ml FGF and 10 ng/ml Platelet Derived Growth Factor AA (PDGF-AA, R&D) (herein referred to **OPC growth media [GM]**) and cultured for 2-3DIV. To induce differentiation, media was changed on 2-3 DIV to SFM supplemented with 2% B27 and 40 ng/ml 3,3',5-Triiodo-L-thyronine (T3, Sigma) (herein referred to **OPC differentiation media [DM]**) and cells were cultured for additional 2-5 days (10).

OPC-Cortical Neuron Co-Cultures:

Cortical Neuron Cultures: Pia layer was removed from P1-P2 brains and cortical tissue from P1-P2 pups was microdissected in Earle's Balanced Salt Solution (EBSS, Gibco) and digested with papain (Worthington) in EBSS. Reaction was stopped with fetal bovine serum (FBS, Invitrogen). Tissue was washed and mechanically dissociated in 10% FBS in DMEM (high glucose, Gibco) with 1% Pen/Strep. Dissociated neurons were seeded at 105,000 cells/cm^2 on plates coated with 40 µg/ml Poly-D-Lysine and 4 µg/ml laminin. 2-4h after plating, media was exchanged to Neurobasal Plus (Invitrogen) with 100 µg/mL L-glutamine, 1% Pen/Strep supplemented with 2% B27 plus supplement (Life Technologies, **Neuron Media I**). After 2 days, wipeout of proliferating, non-neuronal cells was performed by adding fresh **Neuron Media I** with 3 µM Arabinocytoside C (AraC, Sigma) for 48h. AraC and dead cells were washed out with 3 changes of Neurobasal (Invitrogen) with 100 µg/mL L-glutamine, 1% Pen/Strep supplemented with 2% B27 supplement (**Neuron Media II**). Neurons were cultured for additional 4 days, with **Neuron Media II** exchanged every 48h. At this timepoint, cultures contained neurons (50%) and astrocytes (50%) with no microglia or oligodendrocytes (data not shown).

Co-Cultures: OPC cultures were generated from SVZ primary neurospheres as described above from the same P1-P2 pups that were used to generate cortical neuron cultures. On day of co-culture, SVZ OPCs were lifted with 0.025% trypsin (Invitrogen), washed, and resuspended in **Neuron Media II** with 40 ng/ml T3. OPCs were added to neurons at 105,000 cells/cm^2 in the presence of 250 ng/ml FKN or VC (PBS). OPCs were co-cultured with neurons for 2-6 days, where 50% media was replaced every 48h.

Conditioned medium preparation/ELISA: OPCs were cultured in GM as above for 2-3DIV. Conditioned medium was collected and centrifuged at 500g for 7 min to remove dead cells and debris. FKN protein detection was performed via enzyme-linked immunosorbent assay (ELISA) kit (RayBiotech) following

manufacturer's directions. ELISA measurements were performed via technical duplicates from 3 independent preparations of OPC conditioned medium.

Organotypic cerebellar cultures: Developmental myelination was analyzed by using an *ex vivo* organotypic cerebellar slice culture model as previously described (11-13). Briefly, cerebellum from P10-11 CD1 mice was dissected into HBSS ice-cold solution containing 10 mM glucose, 10 mM $MgCl_2$ (Fisher Scientific), 2 mM $CaCl_2$ (Sigma) and 0.1% Pen/Strep. Cerebellar parasagittal slices were cut at 300 µm thickness by using an oscillating tissue slicer (OTS-5000, EMS). Slices were then transferred to a membrane insert (Millipore, 30 mm diameter, pore size 0.4 µm) and cultured using an interface method, with 1 ml of serum-based medium composed of 50% Dulbecco's Modified Eagle Medium with Glutamax-1 (DMEM+Glutamax, Gibco), 24% Earle's Balanced Salt Solution containing calcium, magnesium and phenol red (EBSS, Gibco), D-glucose (0.13 mg/mL, Sigma), 1% Pen/Strep, and 25% horse serum (Gibco). Slices were maintained at 37°C, and kept under 95% O_2 and 5% CO_2 for one day. At this timepoint, medium was removed and replenished with medium containing PBS or IgG (control medium) and 250 ng/mL FKN or 20 µg/mL function blocking antibody specific for murine FKN (please see "Growth factors and function-blocking antibodies" section). ½ media re-supplemented with PBS, IgG, FKN or anti-FKN was exchanged every 2 days. Slices were fixed in 4% PFA after 96h of treatment.

Reagents and Immunostaining:

Immunocytochemistry (ICC): With the exception of cell cultures used for FKN-647 binding assay, cell cultures were fixed with 4% PFA (ACROS Organics) for 10 minutes at room temperature, permeabilized with 0.2% NP-40 (Sigma) for 5 min, and blocked with 6% donkey serum (Jackson ImmunoResearch) in 0.5% BSA in 1X PBS (ICC blocking buffer). Primary antibodies listed in "Antibodies" section were added in ½ ICC blocking buffer and ½ PBS for 2 hours at room temperature or overnight at 4°C. Appropriate secondary antibodies listed in "Antibodies" section were added in 1X PBS for 1 hour at room temperature. For BrdU immunostaining, after incubation with the fluorescently labelled secondary antibodies, cells were washed 3 times with 1X PBS, post-fixed for 10 min with 4% PFA for 10 min at room temperature, washed with 1X PBS, incubated with 1M hydrochloric acid (HCl, Fisher) for 10 min at 4°C and 2M HCl for 20 min at room temperature. After extensive washes with 1X PBS, slides were blocked with 5% donkey serum, 1M glycine (Sigma), and 1% Triton X-100 (Bio Basic) in 1X PBS for 30 minutes at room temperature, then incubated with antibodies specific for BrdU (please see "Antibodies" section) in 1X PBS for 1h at room temperature or overnight at 4°C followed by appropriate secondary antibody for 1h at room temperature. To visualize nuclei, cultures were counter-stained with Hoechst 33258 (Riodel-De Haen Ag) for 2 min. Slides were mounted with Fluoromount G (ThermoFisher).

For FKN-647 *in vitro* binding assay, cell cultures were fixed with 4% PFA for 10 min, and blocked with ICC blocking buffer without permeabilization. FKN-647 or Streptavidin-Cy5 (Jackson ImmunoResearch) at equimolar amounts in ½ ICC block and ½ 1X PBS were added to cell cultures for 2 hours at room temperature or 4°C overnight. Cell cultures were mounted with Fluoromount G or processed for additional antibody staining. In this case, FKN-647 or Cy5-streptavidin incubated cultures were washed with 1X PBS, post-fixed for 10 min with 4% PFA for 10 min at room temperature, washed with 1X PBS, permeabilized and blocked as described above, and incubated with primary antibodies listed in "Antibodies" section Appropriate secondary antibodies conjugated to Alexa555/Cy3 or Alexa488/FITC were used to visualize the signal.

Immunohistochemistry (IHC): Mice over 21 days of age were transcardially perfused with HBSS (Gibco) followed by 4% PFA (Acros Organics) and post-fixed in 4% PFA for additional 24h at 4°C. Mice under 21 days of age were euthanized with CO_2 and dissected brains were fixed in 4% PFA for 16-24h. After 3 changes of 30% sucrose (Fisher) in 1X PBS each lasting 24h, brains were flash-frozen and sectioned at 18 µm. Sections were rehydrated in 1X PBS for 10 min, then permeabilized and blocked with 5% BSA and 0.3% Triton-X100 in 1X PBS for 1 hour at room temperature. Tissue sections were incubated overnight at 4°C with appropriate primary antibodies listed in "Antibodies" section diluted in 5% BSA in 1X PBS. For primary antibodies raised in mouse, a mouse on mouse (MOM) kit was used according to manufacturer's instructions (VectorLabs). Appropriate secondary antibodies diluted in 1X PBS listed in "Antibodies" section were added to tissue sections for 1h at room temperature. For nuclei visualization, tissue sections were incubated with Hoechst 33258 diluted in 1X PBS for 2 minutes at room temperature. Sections were mounted with Fluoromount G.

Immunohistochemistry of organotypic cerebellar slice cultures: Cultured cerebellar slices were quickly washed once with 1X PBS and then fixed with 4% PFA for 1h, followed by 3x washes in 1X PBS and incubation overnight in 1% Triton X-100 in 1X PBS (PBS-T) at 4°C. Next, slices were incubated in a blocking buffer containing 10% BSA, 0.1% Triton X-100 in 1X PBS for 3 h. Primary antibodies listed in "Antibodies section" were added in 6% normal donkey serum, 0.5% BSA and 1% triton X-100 in 1X PBS overnight at 4°C. After that, slices were washed three times in 1X PBS, and then blocking solution consisting of 6% normal donkey serum, 0.5% BSA and 0.1% triton X-100 was added for 1h, followed by addition of secondary antibodies and Hoechst diluted in PBS-T 1% for 3h, at room temperature. Slices were then washed 3 times for 10 min each and mounted with Fluoromount G.

Antibodies: Mouse anti-APC/CC1 (1:300, Calbiochem), mouse anti-βIII (BioLegend, 1:1000, ICC, RRID:AB_10063408), rabbit anti-βIII (BioLegend, 1:2000, RRID: AB_2564645), mouse anti-BCAS1 (Santa Cruz, 1:500, ICC and IHC, RRID: AB_10839529), sheep anti-BrdU (Abcam, 1:1000, ICC, RRID:AB_302659), rabbit anti-CC3, active form (Millipore, 1:250, ICC, RRID: AB_91556), rabbit anti-CX3CL1 (Torrey Pines Biolabs, 20μg/mL, function-blocking, RRID: AB_10891146), rabbit anti-CX3CR1 (Torrey Pines Biolabs, 40 μg/ml, function-blocking, RRID: AB_10892355), rabbit anti-DCX (Abcam, 1:400, ICC, RRID:AB_732011), rabbit anti-GFAP (Dako, 1:1000, ICC and IHC, RRID: AB_10013382), rat anti-GFAP (Thermo Fisher, 1;1000, ICC, RRID:AB_2532994), chicken anti-eGFP (Abcam, 1:1000, IHC, RRID: AB_300798), goat anti-IBA1 (Novus Bio, 1:300 for IHC, 1:1000 for ICC, RRID:AB_521594), rabbit anti-IBA1 (Wako, 1:1000, RRID: AB_839504); rabbit anti-IgG (Jackson, 20 – 40 μg/mL, function-blocking, RRID:AB_97852), mouse anti-Ki67 (BD Pharmingen, 1:300, ICC, RRID: AB_396287), rat anti-MBP (a. a. 82-87) (Millipore, 1:500, ICC and IHC, RRID: AB_94975), mouse anti-Nestin (Abcam, 1:300, ICC, RRID: AB_11211837), chicken anti-NF (light chain, BioTechne, 1:1000, IHC, RRID:AB_1556331), rabbit anti-NG2 (Chondoritin Sulfate Proteoglycan) (Millipore, 1:250, ICC, RRID: AB_91789), rabbit anti-OLIG2 (Millipore, 1:1000 for IHC, 1:2000 for ICC, immunostaining, RRID: AB_570666), mouse anti-OLIG2 (Millipore, 1:1000 for IHC, 1:500 for ICC, RRID: AB_10807410), goat anti-PDGFRα (R&D Systems, 1:400 for IHC, 1:300 for ICC, RRID: AB_2236897), fungus phalloidin-Alexa488 (ThermoFisher, 1:1000, ICC), rabbit anti-SOX2 (Cell Signalling, 1:2000, ICC, RRID: AB_2194037), goat anti-SOX2 (R&D Systems, 1:1000, IHC, RRID: AB_355110). Fluorescently labeled highly cross-absorbed secondary antibodies were purchased from Jackson ImmunoResearch and used at 1:1000 dilution. If MOM kit was used, Cy3-, DTAF-, or Cy5 conjugated streptavidin (Jackson ImmunoResearch) were used at 1:1000 dilution.

RNA Scope: RNA Scope was performed using brain cryosections from P2, P7, P15 and P65 CD1 mice as described in (2) with probes targeting murine *Cx3cr1*, *Olig22*, *Sox2* mRNA or negative control probe purchased from Advanced Cell Diagnostics according to the manufacturer's instructions. Briefly, brains were cryopreserved and sectioned at 18 μm as described in the IHC section. Sections were dehydrated with ethanol and rehydrated in 1X PBS with protease provided by RNA Scope kit and diluted 1:5 in 1xPBS for 10 min at 37°C. Sections were then washed with wash buffer provided by the RNA Scope kit and incubated with probes for 2h at 37°C. Sections were then washed and incubated with signal amplification solutions as per the manufacturer's protocol. Sections were counter-stained with DAPI (provided in the RNA scope kit) to visualize nuclei. Cells with 3 or more RNA Scope dots were considered to be positive for marker expression.

Microscopy: For RNA Scope experiments, sections were imaged with Zeiss LSM700 confocal microscope with photomultiplier tube (PMT) with 40X objective. High magnification images were taken with 5x digital zoom. Digital image acquisition was performed with Zen (Zeiss). Z-stacks of confocal images were taken with optical slice thickness 0.2-0.5 μm and stacked images are shown.

All primary cell culture experiments as well as *in vivo* experiments (except for RNA Scope) were captured with 20X or 40X objectives. Cerebellar slice images were captured by acquiring Z-stacks with optical thickness 1 μm using 10X objective. Images were captured using Zeiss Axio Imager M2 fluorescence microscope, ORCA-Flash LT sCMOS Camera and the Zen software (Zeiss). Cultured cells and *in vivo* images were imaged in single plane, except FKN-647 and BSA-647 brain sections were imaged using Z-stacks with 0.5 μm thickness and stacked images as well as orthogonal sections through 3D projections are shown. Cortical neuron-OPC co-cultures were imaged using 20X objective and Z-stacks with optical slice thickness 0.5 μm. Stacked images as well as optical (orthogonal) sections through 3D projections are shown.

QUANTIFICATION AND STATISTICAL ANALYSIS

Cell cultures were analyzed with a Zeiss Axioimager fluorescence microscope. Digital image acquisition was performed with ZEN (Zeiss) software. In all culture experiments, 5-10 fields of view were captured with a 20X objective. At least 500-2,000 cells from each treatment and biological experiment were counted. For neurosphere experiments, over 100 spheres were analyzed per condition per experiment. In OPC and NPC monolayer cultures, cells with condensed nuclei are presented as relative to total (sum of cells with condensed and healthy) nuclei. For all remaining cell culture experiments, results were presented as marker$^+$ cells relative to total healthy nuclei. Proliferation index is presented as %Ki67$^+$marker$^+$ cells over total marker$^+$ cells. OLs in co-culture were scored by their interaction with neurons. An “interacting MBP$^+$OLIG2$^+$ cell” was defined as either a “contacting MBP$^+$OLIG2$^+$ cell,” in which an MBP$^+$ process was in contact with a βIII$^+$ extension (see Fig. 5L, inset III), or as a “myelinating MBP$^+$OLIG2$^+$ cell,” in which an MBP$^+$ process was wrapping a βIII$^+$ extension (Fig. 5L, insets I-II). “Myelinating MBP$^+$OLIG2$^+$ cells” included MBP$^+$OLIG2$^+$ cells with thin or discontinuous βIII coverage (Fig. 5L, inset I) and MBP$^+$OLIG2$^+$ cells with heavy βIII coverage (Fig. 5L, inset II). Data was presented as either % myelinating MBP$^+$OLIG2$^+$ cells over total MBP$^+$OLIG2$^+$ cells (Fig. 5 M,O) or % interacting MBP$^+$OLIG2$^+$ cells over total MBP$^+$OLIG2$^+$ cells (Fig. 5 N,P).

In vitro and *ex vivo* data are presented from at least 3 independent biological experiments.

For cerebellar slices, myelin index was determined by manually counting the number of intersections of NF$^+$MBP$^-$ and NF$^+$MBP$^+$ fibres with 10 equidistant grids overlaid in Fiji software using same-area box (14) as described in (13, 15) (Fig. S3C,E and Fig. S4M,O). Proportion of myelinated axons indicates a sum of NF$^+$MBP$^+$ axons divided by a sum of total NF$^+$ axons in 10 grids. OLIG2$^+$ and CC1$^+$ cells were analyzed by applying same size box to cerebellar white matter. At least 2 slices per each biological replicate and 300 cells per replicate were analyzed. GFAP mean fluorescence intensity was measured in Fiji software by applying the same size box to cerebellar white matter.

For all *in vivo* experiments, dorsolateral, ventrolateral or dorsal SVZ and/or neighbouring corpus callosum was imaged using 20X objective as indicated in figures. Areas of interest were identified with Hoechst staining. For FKN-infusion experiments, the results are presented as percentage of marker$^+$YFP$^+$ cells relative to total YFP$^+$ cells. 5-8 anatomically matched sections per brain were analyzed from 6 mice across 3 independent litters. At least 250 cells per brain were counted. For FKN-647 experiments, results are presented as FKN-647$^+$marker$^+$ cells relative to total marker$^+$ cells. 3 anatomically matched sections were analyzed from 2 mice.

Images were counted in Zen or Fiji software (14). Representative images were processed in Photoshop CC 2015 and figures in Adobe Illustrator CC 2015. Biorender was used to generate cerebellar slice schematics and graphical abstract.

Sample sizes (n) indicated in the figure legends 2-7 correspond to the number of biological replicates analyzed; for RNA Scope in Fig. 1, Fig. S1 and FKN-647 infusion in Fig. 2A-G, Fig. S2A-E, data is presented from 2-4 mice per age or treatment. All data are presented as mean ± SEM.

For two group comparisons, two-tailed paired student’s t-tests (*in vitro* and *ex vivo* datasets) or two-tailed unpaired student’s t-tests (for *in vivo* datasets) or multiple t-tests were used to assess statistical significance between means, where a p-value <0.05 was considered significant. For three or more group comparisons one-way ANOVA was followed by Dunnett’s multiple comparisons test. In all cases, Prism (version 8.0.2) was used. Number of experiments and statistical information are stated in the corresponding figure legends. In figures, asterisks denote statistical significance marked by *, $p < 0.05$; **, $p < 0.01$; ***, $p < 0.001$.

Supplemental References:

1. Mizrak D, Levitin HM, Delgado AC, Crotet V, Yuan J, Chaker Z, et al. Single-Cell Analysis of Regional Differences in Adult V-SVZ Neural Stem Cell Lineages. Cell Rep. 2019;26(2):394-406.e5.

2. Voronova A, Yuzwa SA, Wang BS, Zahr S, Syal C, Wang J, et al. Migrating Interneurons Secrete Fractalkine to Promote Oligodendrocyte Formation in the Developing Mammalian Brain. Neuron. 2017;94(3):500-16.e9.

3. Stolla M, Pelisek J, von Brühl M-L, Schäfer A, Barocke V, Heider P, et al. Fractalkine is expressed in early and advanced atherosclerotic lesions and supports monocyte recruitment via CX3CR1. PloS one. 2012;7(8):e43572-e.

4. Gu X, Xu J, Yang X-P, Peterson E, Harding P. Fractalkine neutralization improves cardiac function after myocardial infarction. Exp Physiol. 2015;100(7):805-17.

5. Cipriani R, Villa P, Chece G, Lauro C, Paladini A, Micotti E, et al. CX3CL1 is neuroprotective in permanent focal cerebral ischemia in rodents. J Neurosci. 2011;31(45):16327-35.

6. Imayoshi I, Ohtsuka T, Metzger D, Chambon P, Kageyama R. Temporal regulation of Cre recombinase activity in neural stem cells. Genesis. 2006;44(5):233-8.

7. Cardona SM, Kim SV, Church KA, Torres VO, Cleary IA, Mendiola AS, et al. Role of the Fractalkine Receptor in CNS Autoimmune Inflammation: New Approach Utilizing a Mouse Model Expressing the Human CX3CR1(I249/M280) Variant. Frontiers in cellular neuroscience. 2018;12:365-.

8. Storer MA, Gallagher D, Fatt MP, Simonetta JV, Kaplan DR, Miller FD. Interleukin-6 Regulates Adult Neural Stem Cell Numbers during Normal and Abnormal Post-natal Development. Stem Cell Reports. 2018;10(5):1464-80.

9. Coles-Takabe BL, Brain I, Purpura KA, Karpowicz P, Zandstra PW, Morshead CM, et al. Don't look: growing clonal versus nonclonal neural stem cell colonies. Stem Cells. 2008;26(11):2938-44.

10. Bhat NR, Sarlieve LL, Rao GS, Pieringer RA. Investigations on myelination in vitro. Regulation by thyroid hormone in cultures of dissociated brain cells from embryonic mice. J Biol Chem. 1979;254(19):9342-4.

11. Birgbauer E, Rao TS, Webb M. Lysolecithin induces demyelination in vitro in a cerebellar slice culture system. J Neurosci Res. 2004;78(2):157-66.

12. Stoppini L, Buchs PA, Muller D. A simple method for organotypic cultures of nervous tissue. J Neurosci Methods. 1991;37(2):173-82.

13. de Almeida MMA, Pieropan F, de Mattos Oliveira L, dos Santos Junior MC, David JM, David JP, et al. The flavonoid agathisflavone modulates the microglial neuroinflammatory response and enhances remyelination. Pharmacological Research. 2020;159:104997.

14. Schindelin J, Arganda-Carreras I, Frise E, Kaynig V, Longair M, Pietzsch T, et al. Fiji: an open-source platform for biological-image analysis. Nature Methods. 2012;9(7):676-82.

15. Al-Griw MA, Wood IC, Salter MG. Cerebellar Organotypic Slice Culture System: A Model of Developing Brain Ischaemia. Life Science Journal. 2017;14(4):89-98.
